# Eosinophil‐derived IL‐4 is necessary to establish the inflammatory structure in innate inflammation

**DOI:** 10.15252/emmm.202216796

**Published:** 2022-12-21

**Authors:** Anja Kolbinger, Tim J Schäufele, Hanna Steigerwald, Joschua Friedel, Sandra Pierre, Gerd Geisslinger, Klaus Scholich

**Affiliations:** ^1^ Institute of Clinical Pharmacology Goethe‐University Frankfurt Frankfurt Germany; ^2^ Fraunhofer Institute for Translational Medicine and Pharmacology ITMP Frankfurt Germany; ^3^ Fraunhofer Cluster of Excellence for Immune‐Mediated Diseases CIMD Frankfurt Germany

**Keywords:** eosinophil, innate inflammation, interleukin‐4, macrophages, microenvironments, Immunology

## Abstract

Pathogen‐induced inflammation comprises pro‐ and anti‐inflammatory processes, which ensure pathogen removal and containment of the proinflammatory activities. Here, we aimed to identify the development of inflammatory microenvironments and their maintenance throughout the course of a toll‐like receptor 2‐mediated paw inflammation. Within 24 h after pathogen‐injection, the immune cells were organized in three zones, which comprised a pathogen‐containing “core‐region”, a bordering proinflammatory (PI)‐region and an outer anti‐inflammatory (AI)‐region. Eosinophils were present in all three inflammatory regions and adapted their cytokine profile according to their localization. Eosinophil depletion reduced IL‐4 levels and increased edema formation as well as mechanical and thermal hypersensitivities during resolution of inflammation. Also, in the absence of eosinophils PI‐ and AI‐regions could not be determined anymore, neutrophil numbers increased, and efferocytosis as well as M2‐macrophage polarization were reduced. IL‐4 administration restored in eosinophil‐depleted mice PI‐ and AI‐regions, normalized neutrophil numbers, efferocytosis, M2‐macrophage polarization as well as resolution of zymosan‐induced hypersensitivity. In conclusion, IL‐4‐expressing eosinophils support the resolution of inflammation by enabling the development of an anti‐inflammatory framework, which encloses proinflammatory regions.

## Graphical abstract

Multiplex immunohistochemistry identified a regional architecture in TLR‐type 2 inflammation, which comprised the pathogen‐containing core region, a surrounding pro‐inflammatory region, and an outer anti‐inflammatory region. Once established, the regions were stable throughout the course of inflammation.

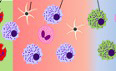

## Introduction

Pathogen‐induced inflammation involves pro‐ and anti‐inflammatory processes to ensure pathogen removal as well as containment and resolution of the proinflammatory activities. After tissue intrusion by pathogens, a complex innate immune response is triggered by local cells following the recognition by toll‐like receptors (TLR), which initiate the recruitment of local and blood‐derived innate immune cells. Neutrophilic and eosinophilic granulocytes are among the first cell types recruited to the tissue followed, with some delay, by monocytes and dendritic cells (DC) (Kolaczkowska & Kubes, 2013; Lastrucci *et al*, 2015). Once having crossed the endothelium and entered the inflamed tissue the immune cells react according to the signals they receive from their microenvironment and initiate cell type‐specific responses such as the release of cytotoxic compounds, phagocytosis or a further release of proinflammatory mediators. The entity of all cells interacting with an immune cell are forming its microenvironment.

The best understood example for the influence of microenvironments on immune cells is the polarization of macrophages toward proinflammatory M1‐like or anti‐inflammatory M2‐like phenotypes depending on their microenvironment (Imhof & Aurrand‐Lions, 2004; Shi & Pamer, 2011). Macrophages exhibit various reversible phenotypic states within the M1/M2 spectrum whereby changes in the microenvironment induce a corresponding transcriptional reprogramming (Murray & Wynn, 2011; Okabe & Medzhitov, 2014). As a consequence, the specific positioning of macrophages within the inflamed tissue decides their polarization fate as demonstrated by the fatty acid/lactate receptor G2A (GPR132), which indirectly regulates macrophage polarization towards proinflammatory M1‐like phenotypes by positioning the macrophages in a proinflammatory microenvironment (Kern *et al*, 2018). Similar environment‐induced changes might also occur in other cells types. For example, eosinophils have various pro‐ and anti‐inflammatory mediators, which can be rapidly released from specific granules and lipid bodies according to the situation (Weller & Spencer, 2017; Rigoni *et al*, 2018). However, tissue‐specific subsets of pro‐resolution eosinophils in the synovium and proinflammatory eosinophils in lungs have been described (Andreev *et al*, 2020). Moreover, it was shown that eosinophils can polarize toward distinct Type 1 or Type 2 inflammatory response phenotypes (Dolitzky *et al*, 2021).

To better comprehend the interaction between cellular microenvironments and immune cells during an ongoing inflammation, it is crucial to understand how these cellular microenvironments are formed and maintained. Important information about the microenvironment of immune cells are normally rudimentary due to the limited numbers of markers, which can be visualized by classical immunohistochemical approaches. Therefore several methods have been developed in the last years to visualize dozens of cellular marker on the same tissue, allowing investigation of formation and regulation of cellular networks under physiological or pathophysiological conditions (Hoch *et al*, 2022; Moldoveanu *et al*, 2022). Here, we used the multiepitope‐ligand‐carthography (MELC) technology for multiple sequential immunohistochemistry to visualize 40 antibodies on the same tissue (Schubert *et al*, 2006; Friedenberger *et al*, 2007). Established bioinformatics approaches (Schapiro *et al*, 2017; Kornstädt *et al*, 2020; Kolbinger *et al*, 2022) were applied to analyze the formation of the basic inflammatory structure based on the immune cell distribution. Using eosinophils as an example, we determined the impact of distinct microenvironments on immune cells and investigated the role of a specific immune cell type, using eosinophils as example, on the formation of microenvironments.

## Results

### Macrophages define distinct subregions of a local zymosan‐induced inflammation

**Figure 1:**
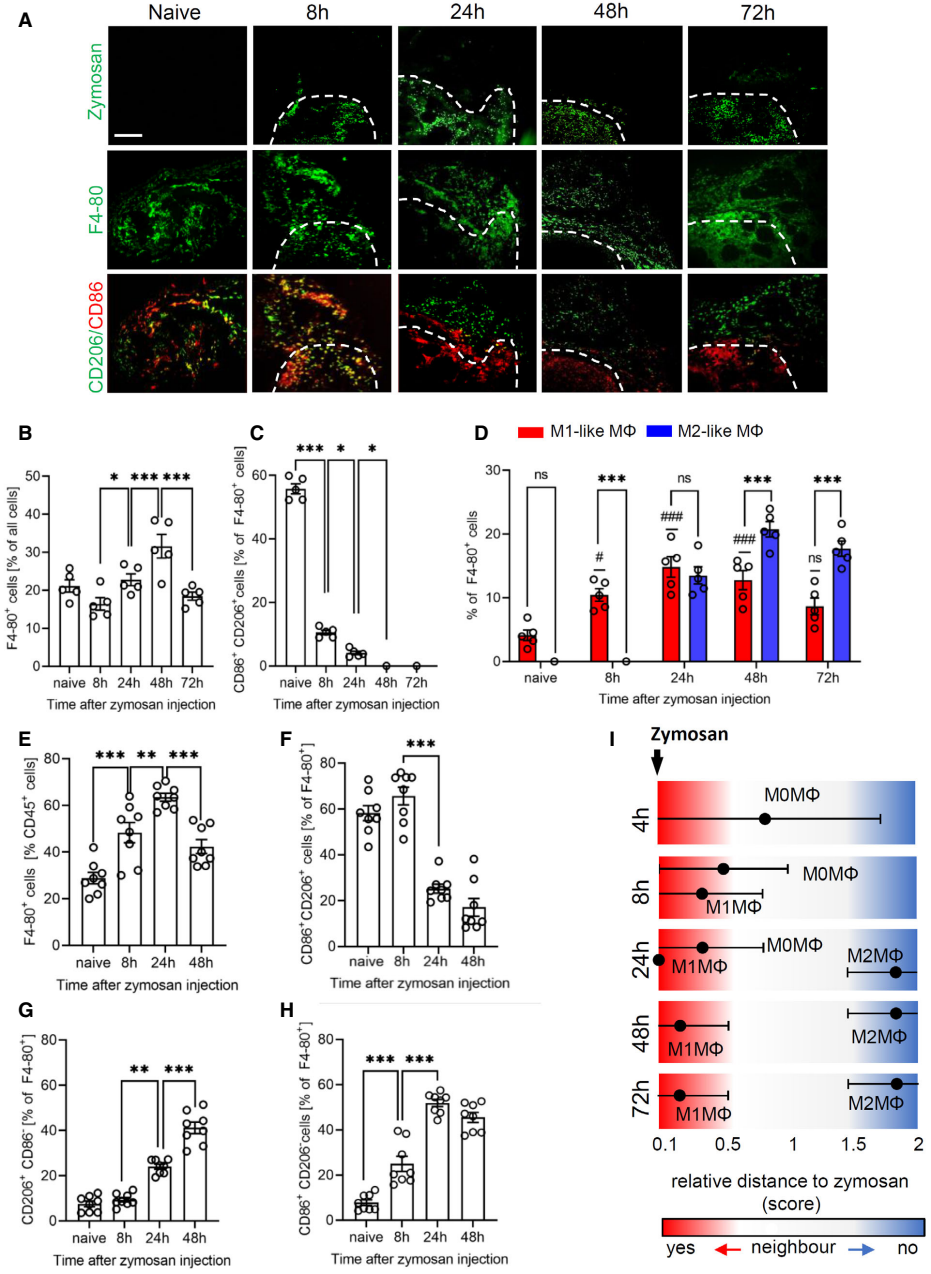
M1‐ and M2‐like macrophages arrange at distinct distances around zymosan ARepresentative MELC images showing the distribution of macrophage polarization markers at the indicated time points after injection of FITC‐labeled zymosan (3 mg/ml, 10 μl) in one hind paw. White dotted lines show the position of zymosan. Size bar represents 100 μm.B–DQuantification of the number of macrophages and their subtypes 8, 24, 48, and 72 h after zymosan injection based on the MELC images. Data are shown as mean (*n* = 5 mice) ± SEM, one‐way ANOVA/Bonferroni *P < 0.05, ***P < 0.001. ^#^ *P* < 0.05, ^###^ *P* < 0.001 as compared to naïve mice.E–HFACS analysis of macrophages and their subtypes 8, 24 and 48 h after zymosan injection. Data are shown as mean (*n* = 8 mice) ± SEM, one‐way ANOVA/Bonferroni **P < 0.01, ***P < 0.001.IRelative distances from the zymosan‐containing area based on the probability for an immediate neighborhood of macrophage subtypes at the indicated time points after zymosan injection. Data are shown as mean (*n* = 5 mice) ± SEM. Source data are available online for this figure.

**Figure EV1:**
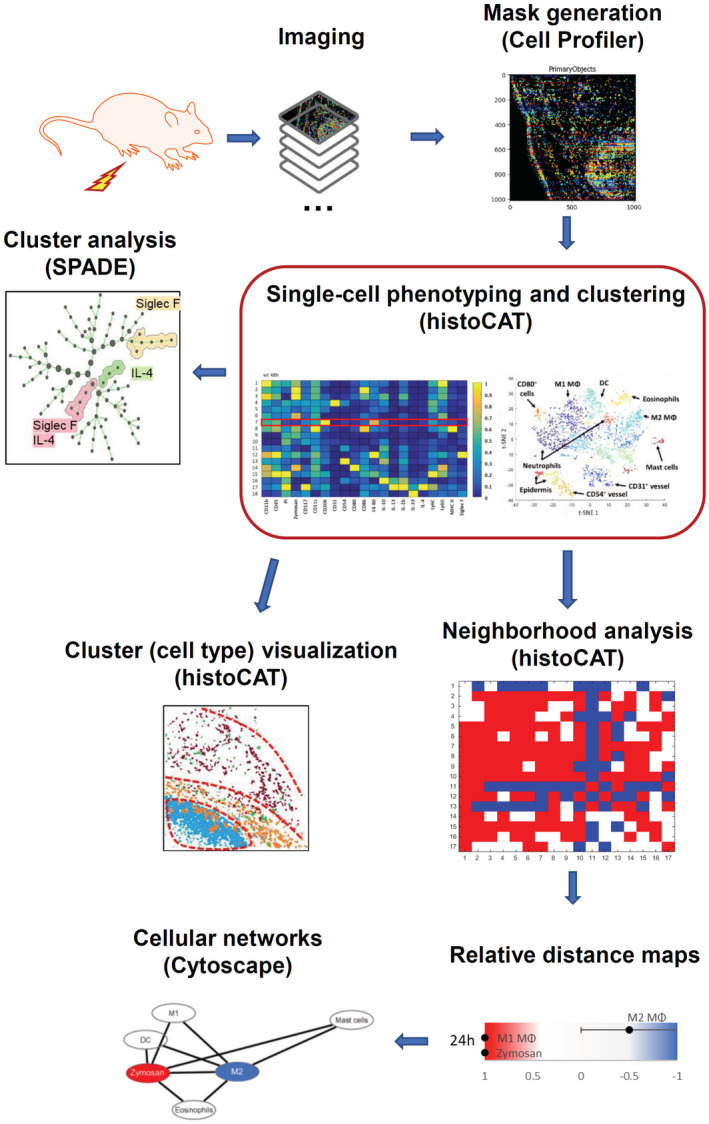
Workflow of the bioinformatic analysis for the MELC images

Zymosan‐induced paw inflammation is a common model to study TLR‐2‐mediated innate immune responses. It is especially suited to investigate the distribution of immune cells in relation to the pathogen, since the particulate structure of zymosan immobilizes it at the site of injection and usage of FITC‐labeled zymosan allows to determine its exact localization in the tissue (Pierre *et al*, 2017; Kern *et al*, 2018). To detect immune cells, the MELC technology (Schubert *et al*, 2006; Schuh *et al*, 2014) was used, 40 antibodies were used to identify and localize immune and nonimmune cells in the tissue (Appendix Table S1). The field of visions (600 × 600 μm) were chosen to cover a part of the zymosan‐containing region together with its adjacent areas, whereby the zymosan covered area represented around 30% of the field of visions. For quantitative assessment of cells, a segmentation mask was generated based on the staining for CD45 and nuclei (Propidiumiodide), which was then used to extract single‐cell expression data from images of all measured markers (Fig EV1). Single‐cell phenotyping was performed using PhenoGraph analysis and was followed by the identification of cell clusters representing the different immune cell types. It should be noted that for better clarity only the markers are explicitly mentioned in the text, which were used to define a specific cell type. In the first step, we used this bioinformatic pipeline to track macrophage polarization over the course of the zymosan‐induced inflammation. We found that within and adjacent to the zymosan‐containing area 8 h after zymosan injection cell clusters representing macrophages started to diverge from an undifferentiated phenotype (M0 macrophages, defined by expression of Siglec F^−^/F4‐80^+^/CD86^+^/CD206^−^) to M1‐like macrophages (Siglec F^−^/F4‐80^+^/CD86^+^/CD206^−^) and after 24 h also to M2‐like phenotypes (Siglec F^−^/F4‐80^+^/CD86^−^/CD206^+^; Fig 1A–D; Appendix Fig S1). Markers for dendritic cells such as CD11c and MHC II were absent in clusters considered as representing macrophages. The time course of macrophage polarization seen in MELC analyses was confirmed by FACS analysis of the paw tissue demonstrating that the macrophage populations in the observed areas are representative for the macrophages in the inflamed paw (Fig 1E–H). Macrophage specific expression of CD86 and CD206 was ensured by applying the gating strategy shown in Appendix Fig S3. The immunohistochemical staining suggested that starting at 24 h after zymosan injection M1‐like macrophages were located within the zymosan‐containing area or in its close proximity, whereas M2‐like macrophages were localized in greater distance to zymosan (Fig 1A). To quantify this observation a bioinformatic assessment of the relative distance of zymosan to clusters representing M1‐ and M2‐like macrophages was performed using a neighborhood analysis. Here, the likelihood of cells neighboring each other is determined in comparison to a randomized version of the same tissue (Schapiro *et al*, 2017). The analysis confirmed a spatial pattern with a direct neighborhood of M1‐like macrophages to zymosan, while M2‐like macrophages were excluded as direct neighbors of zymosan (Fig 1I; Appendix Fig S3).

**Figure 2:**
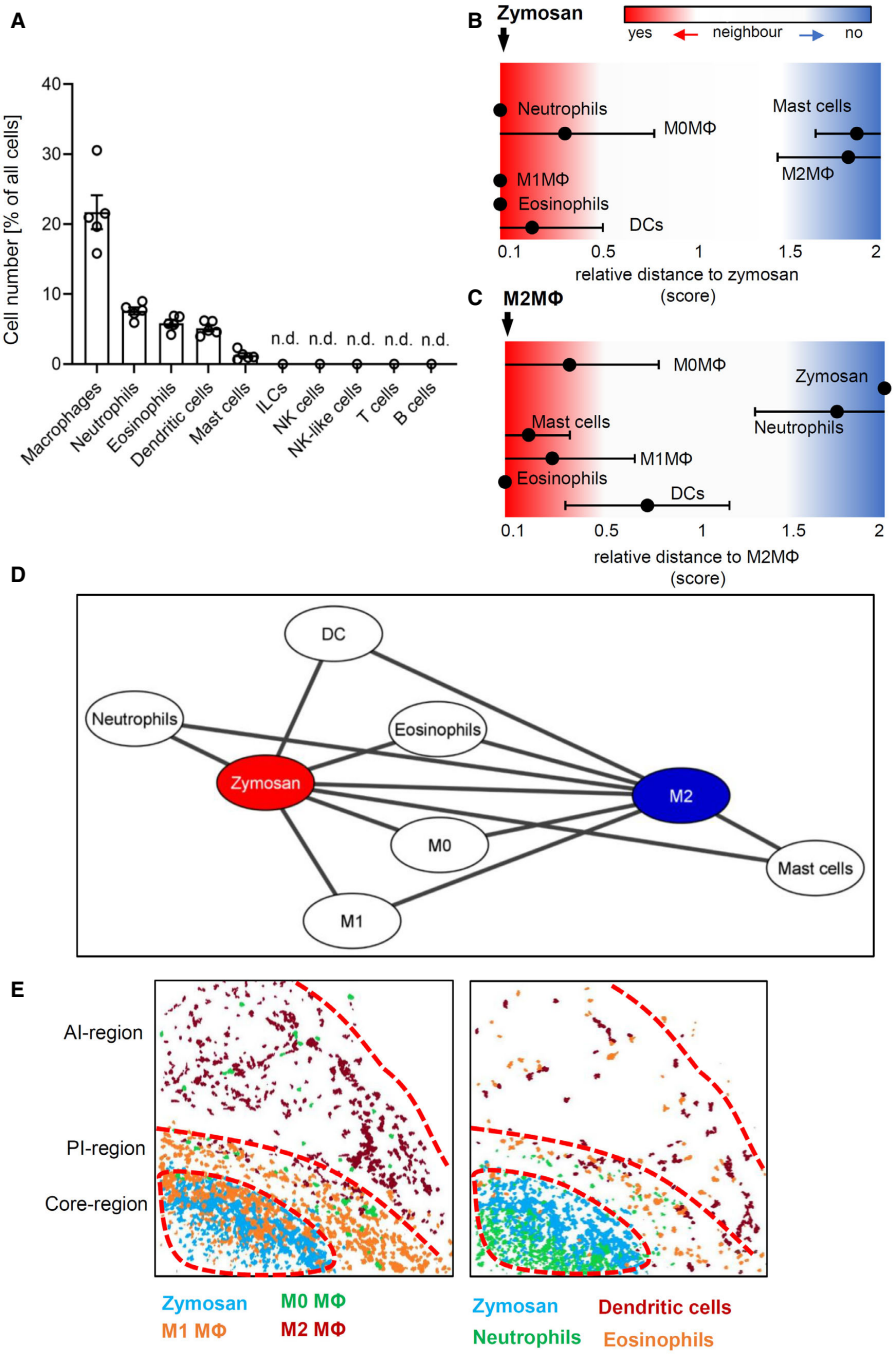
Based on immune cell distribution three inflammatory regions can be defined AFrequency of different immune cell types in the MELC images 24 h after zymosan injection. Data are shown as mean (*n* = 5 mice) ± SEM (n.d., not determinable).B, CRelative distance of various immune cell types based on the likelihood for an direct neighborhood of macrophage subtypes in regard to zymosan (panel B) or M2‐like macrophages (panel C).DNetwork visualization using Cytoscape of the cellular neighborhoods of zymosan and M2‐like macrophages. The distances between the cells in the visualization represents the statistical likelihood of being direct neighbors.EComposite MELC images showing the position of the core‐, the proinflammatory (PI) and the anti‐inflammatory (AI) regions in the tissue in regard to the localization of M1‐ and M2‐like macrophages (MΦ) and zymosan (left panel) or eosinophils, neutrophils and DCs (right panel). The red dotted lines depict the area where the transition between the neighboring regions occurs. Source data are available online for this figure.

**Figure EV2:**
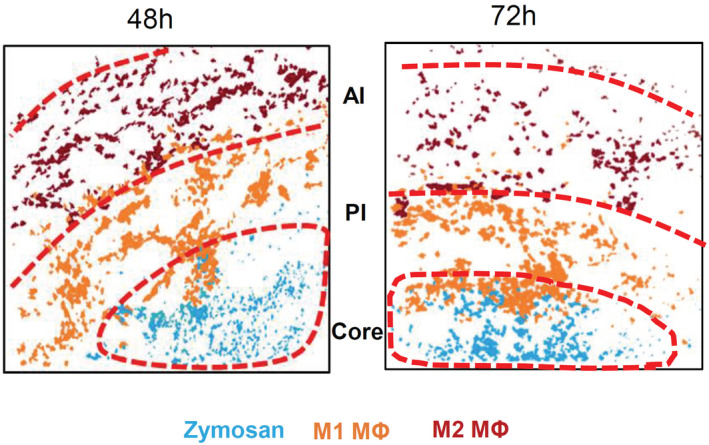
Distribution of macrophage subpopulation 48 and 72 h after zymosan injection Composite MELC images 24 and 72 h after zymosan injection showing the position of the core‐, PI‐ and the AI‐regions in the tissue in regard to the localization of M1‐ and M2‐like macrophages (MΦ) and zymosan. The red dotted lines depict the area where the transition between the neighboring regions occurs.

To determine which immune cells were localized in the inflamed tissue, cell clusters representing immune cells were quantified 24 h after zymosan injection. The most abundant immune cell clusters were macrophages (defined by CD45^+^/Ly6C^−/+^/F4‐80^+^/CD11b^+^), neutrophils (CD45^+^/Ly6G^+^), eosinophils (CD45^+^/Siglec F^+^/F4‐80^+^), DCs (CD45^+^/F4‐80^−^/CD11c^+^) and mast cells (CD117^+^/cytokeratin^−^) (Fig 2A). Clusters representing B‐cells (CD45^+^/CD19^+^ or CD22^+^), T‐cells (CD45^+^/CD3^+^/CD4^−/+^/CD8^−/+^), NK‐ and NK‐like cells (CD45^+^/CD3^−/+^/NK1.1^−/+^), and ILCs (CD45^+^/CD3^−^/GATA3^−/+^/T‐Bet^−/+^/RORγT^−/+^) were not found in the observed areas of the inflamed tissue (Fig 2A). Next, we created a linear map of the immune cell distribution using either zymosan or M2‐like macrophages as reference points, since they represent the contrasting pro‐ and anti‐inflammatory extremes. Direct neighbors of zymosan were cell clusters representing M1‐like macrophages (defined by F4‐80^+^/CD86^+^/CD206^−^), neutrophils (Ly6G^+^/F4 80^−^), DCs (CD11c^+^/MHC II^+^/CD86^+^), and eosinophils (Ly6C^+^/F4‐80^+^/SiglecF^+^) (Fig 2B). In the direct neighborhood of M2‐like macrophages were, in accordance to a recent report, mast cells (CD117^+^) (Kornstädt *et al*, 2020) as well as M1‐like macrophages, DCs, and eosinophils (Fig 2C). The scores received by the neighborhood analysis were plotted to visualize the data as a combined dual‐centered network based on zymosan and M2‐like macrophages (Fig 2D). The network analyses in combination with the visualization of the major immune cell clusters in the tissue (Fig 2E) marked a proinflammatory “core‐region” defined by the presence of zymosan. The core‐region is bordered by a proinflammatory region (PI) comprising the area containing M1‐like macrophages outside the core region. The PI‐region is then adjoined by an anti‐inflammatory region (AI), which is defined as the M2‐like macrophage‐containing area (Fig 2E). Neutrophils were mainly found in the core region and to a lesser degree in the PI region. Mast cells, as direct neighbors of M2‐like macrophages, were localized in the AI‐region. DCs and eosinophils were evenly distributed throughout the three inflammatory regions (Fig 2E, Appendix Fig S4A,B). Notably, the inflammatory architecture, consisting of core‐, PI and AI‐regions, was also observed at later time points during the inflammation demonstrating that this structure provides a stable framework for the inflammatory response until the pathogen is removed (Fig EV2).

**Figure 3:**
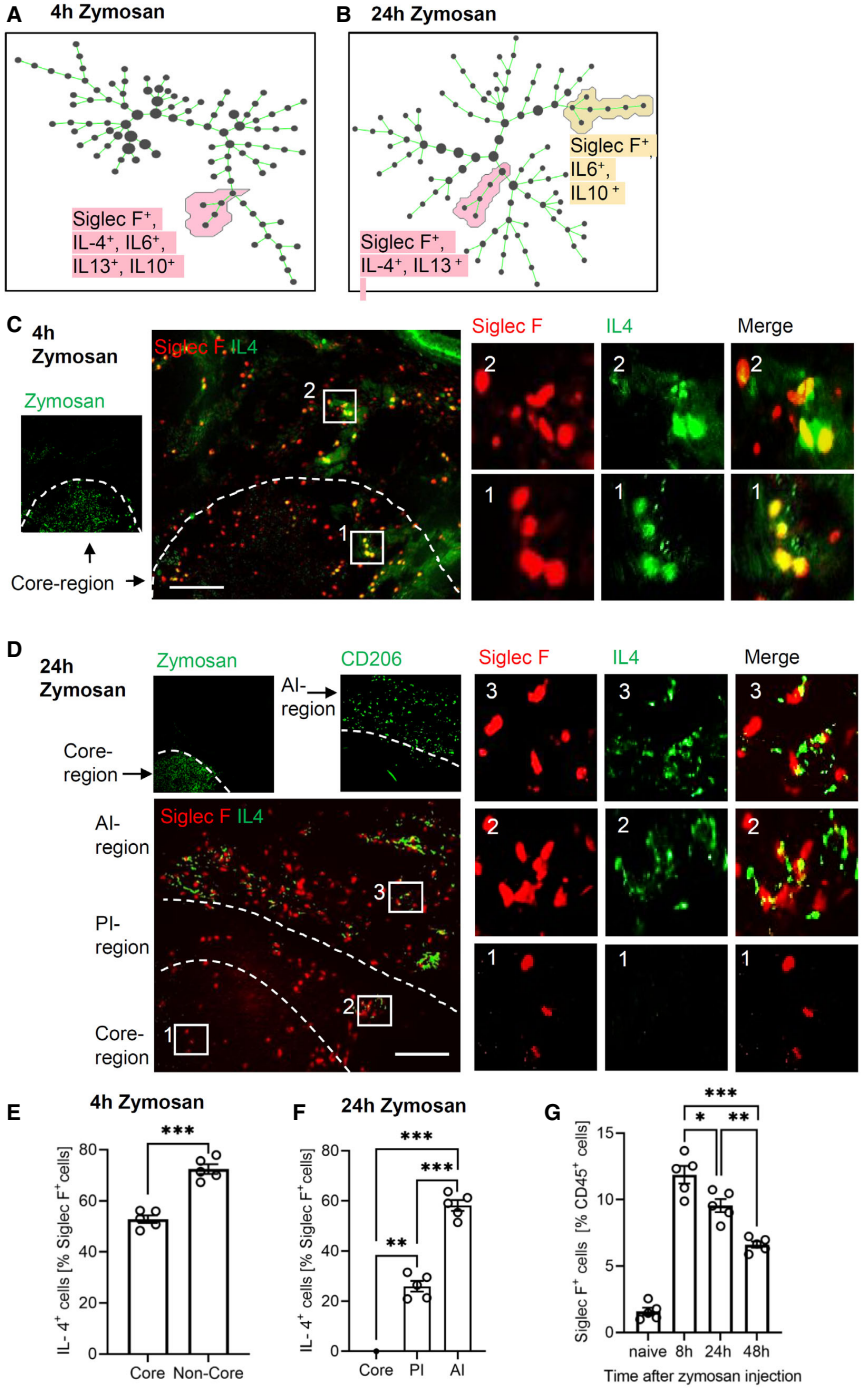
Expression of IL‐4 in eosinophils depends on their localization A, BIdentification of eosinophil populations using SPADE analysis 4 (panel A) and 24 h (panel B) after zymosan injection.C, DRepresentative MELC images showing the IL‐4 and Siglec F expression 4 h (panel C) and 24 h (panel D) after zymosan injection. The white dotted lines depict the area where the transition between the neighboring regions occurs. Size bar represents 100 μm.ENumber of IL‐4‐expressing eosinophils in the core region and the surrounding area 4 h after zymosan injection. Data are shown as mean (*n* = 5 mice) ± SEM, two‐tailed Students *t*‐test ***P < 0.001.FNumber of IL‐4‐expressing eosinophils in the three regions 24 h after zymosan injection. Data are shown as mean (*n* = 5 mice) ± SEM, one‐way ANOVA/Bonferroni **P < 0.01, ***P < 0.001.GFACS analysis of the number of eosinophils at the indicated time points after zymosan injection in the inflamed paw. Data are shown as mean (*n* = 5 mice) ± SEM, one‐way ANOVA/Bonferroni *P < 0.05, **P < 0.01, ***P < 0.001. Source data are available online for this figure.

### Expression of IL‐4 in eosinophils depends on their localization

Since cell clusters representing eosinophils and DCs were found in all three inflammatory regions, we determined whether region‐dependent subtypes can be identified 24 h after zymosan injection, the time point when inflammatory regions and macrophage polarization were established. SPADE cluster analysis did not reveal distinct major DC subtypes based on the antibodies used (Appendix Fig S4C). In contrast, 4 h after zymosan injection only one major eosinophil population was seen (Fig 3A) while after 24 h two discrete major eosinophil subpopulations appeared (Fig 3B). Four hours after zymosan injection eosinophils expressed IL‐4, IL‐6, IL‐13, and IL‐10 while after 24 h eosinophils expressed either IL‐4 and IL‐13 or IL‐6 and IL‐10. Accordingly, IL‐4‐expressing eosinophils were evenly distributed throughout the monitored area including the core region 4 h after zymosan injection (Fig 3C) whereas after 24 h the IL‐4‐positive eosinophil subpopulation was not found in the core‐region (Fig 3D). Since in early inflammation (4 h), all eosinophils express IL‐4, the downregulation of IL‐4 expression within the core region is likely an adaption to this microenvironment. Quantification of the percent of IL‐4‐expressing eosinophils 4 h after zymosan injection showed approximately 20% more IL‐4 expressing eosinophils outside the core region (Fig 3E). Twenty‐four hours after zymosan injection no IL‐4 expressing eosinophils were detected in the core region, while in the AI region and the PI region, the percentage of IL‐4‐expressing cells increased to 25 and 60%, respectively (Fig 3F). Notably, eosinophil numbers in the paw were initially high and declined after 8 h (Fig 3G) suggesting that the recruitment of eosinophils slows or stops after 8 h and that the remaining eosinophils adapt to their specific microenvironment by downregulation of IL‐4 expression.

### Eosinophil depletion disrupts the inflammatory structure and resolution of inflammation

**Figure 4:**
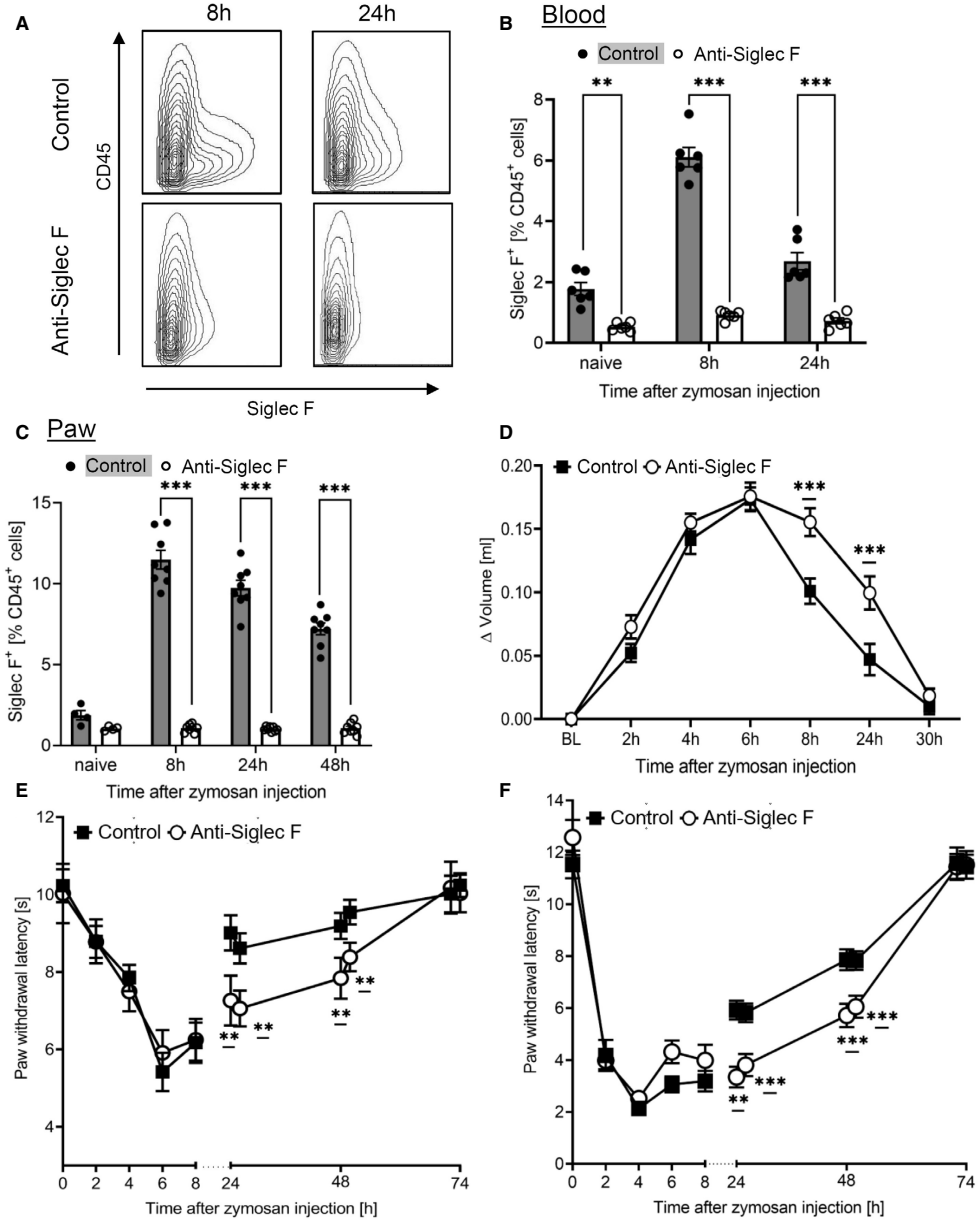
Eosinophil depletion delays resolution of zymosan‐induced inflammation A–CFACS analysis of eosinophils in the blood (panel A,B; *n* = 6 mice) or paws (panel C; *n* = 8 mice) at the indicated time points after zymosan injection. Anti‐Siglec F antibody or the IgG‐control (control) was administered with 0.883 mg/kg 24 h prior zymosan injection. Data are shown as mean ± SEM, student's *t*‐test *P < 0.05, **P < 0.01, ***P < 0.001 vs. control mice.DPaw volume after injection of zymosan. Data are presented as mean ± SEM (*n* = 8–10 mice). Two‐way ANOVA/Bonferroni, ***P < 0.001.E, FZymosan‐induced mechanical (panel E) and thermal (panel F) hypersensitivity of control or anti‐Siglec F antibody‐treated mice. Data are shown as the mean ± SEM (*n* = 8 mice). Two‐way ANOVA/Bonferroni, **P < 0.01, ***P < 0.001 vs. control mice. Source data are available online for this figure.

Next, we depleted eosinophils using anti‐Siglec F antibodies following established protocols (Zimmermann *et al*, 2008; Driss *et al*, 2016; Wang *et al*, 2021). Compared to the isotype control the treatment with anti‐Siglec F antibody reduced the number of eosinophils by around 90% in blood and inflamed paws (Fig 4A–C). Notably, the anti‐Siglec F antibody used for depletion antibody did not interfere with binding of the detection antibody (Appendix Fig S5). Eosinophil depletion increased edema formation during its resolution phase without altering onset or maximal size of edema formation (Fig 4D). Also, the resolution of mechanical and thermal zymosan‐induced hypersensitivity was delayed by eosinophil depletion without affecting the onset or the maximal behavioral response (Fig 4E and F) demonstrating that eosinophils fulfill predominantly anti‐inflammatory functions in this inflammation model.

**Figure 5:**
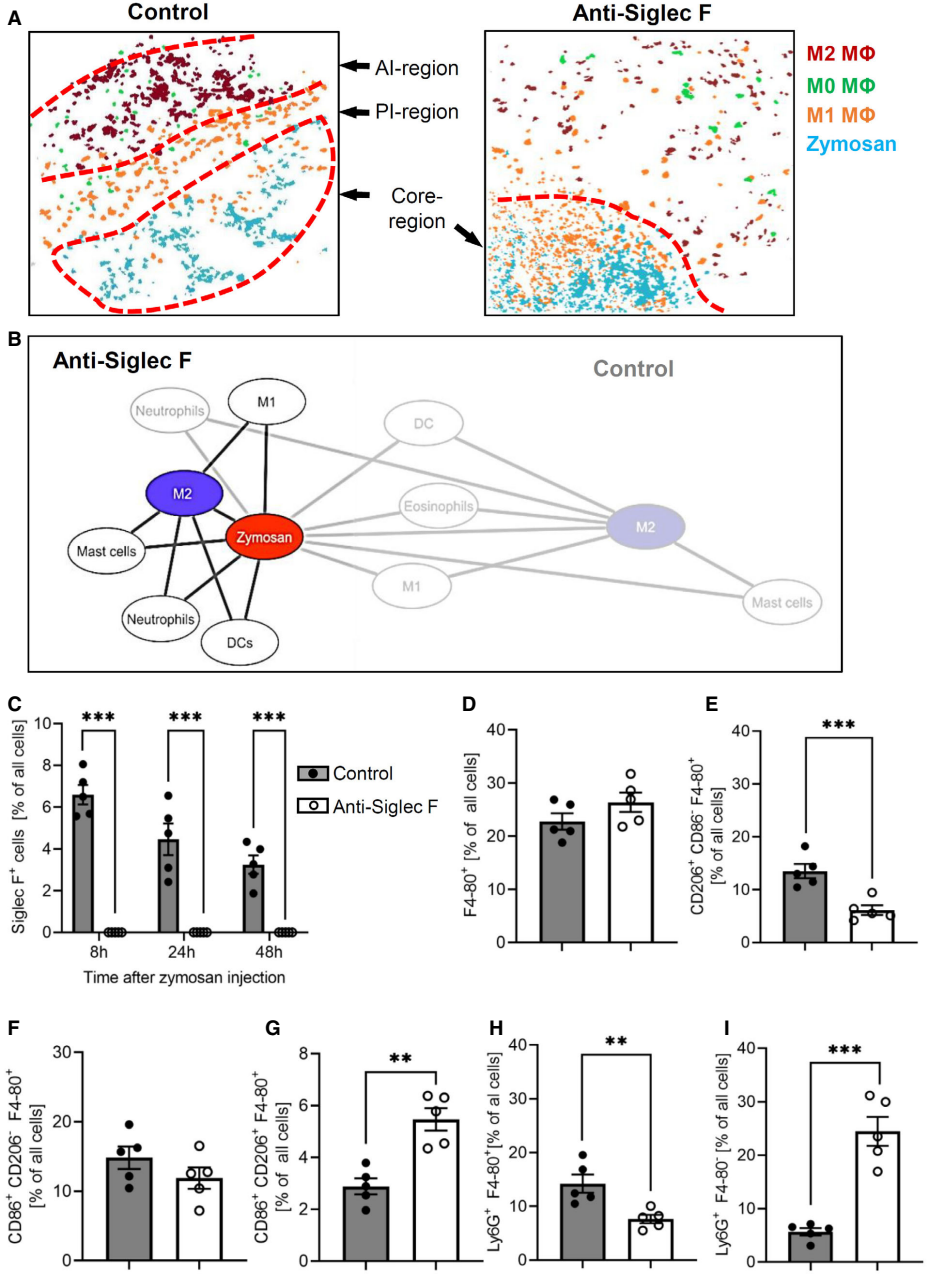
Eosinophils maintain inflammatory regions AComposite MELC image showing the disappearance of the PI‐ and the AI‐regions 24 h after zymosan injection in control or eosinophil‐depleted mice. The red dotted lines depict the area where the transition between the neighboring regions occurs.BNetwork visualization of the cellular neighborhoods of zymosan and M2‐like macrophages 24 h after zymosan injection in eosinophil‐depleted mice superimposed on control (transparent labels and lines as shown in Fig 2C).C–IQuantification of eosinophils, the sum of all macrophages, M2‐like, M1‐like, and M0‐macrophages (panels C–G) as well as efferocytosis (panel H) and neutrophils (panel I) in the MELC images of paws from control or eosinophil‐depleted mice 24 h after zymosan injection. Data are presented as mean ± SEM (*n* = 5). Student's *t*‐test, **P < 0.01, ***P < 0.001. Source data are available online for this figure.

On the cellular‐level eosinophil depletion caused a breakdown of the boundaries between the AI‐ and PI‐regions, marked by an even distribution of M1‐ and M2‐like macrophages around the core‐region (Fig 5A). Fittingly, superimposition of the cellular networks of zymosan and M2‐like macrophages showed in eosinophil‐depleted mice a reduction of the relative distances for basically all observed immune cells toward a direct neighborhood (Fig 5B). Further analysis of the cell clusters derived from the MELC analysis showed a complete absence of eosinophils at the site of inflammation after anti‐Siglec F treatment (Fig 5C), while the number of macrophages was not altered (Fig 5D). Importantly, M2‐like macrophage numbers were significantly decreased in the observed areas (Fig 5E), whereas the number of M1‐like macrophages was not changed (Fig 5F) and the number of M0‐macrophages increased (Fig 5G). The altered macrophage polarization was accompanied by a decreased efferocytosis (Ly6G^+^/F4‐80^+^; Fig 5H) and increased neutrophil numbers (Fig 5I). Taken together, the consequences of eosinophil depletion in regard to the decreased M2‐like macrophage polarization support the *in vivo* data showing a predominantly anti‐inflammatory effect of the eosinophils.

### Eosinophil‐derived IL‐4 mediates macrophage polarization

**Figure 6:**
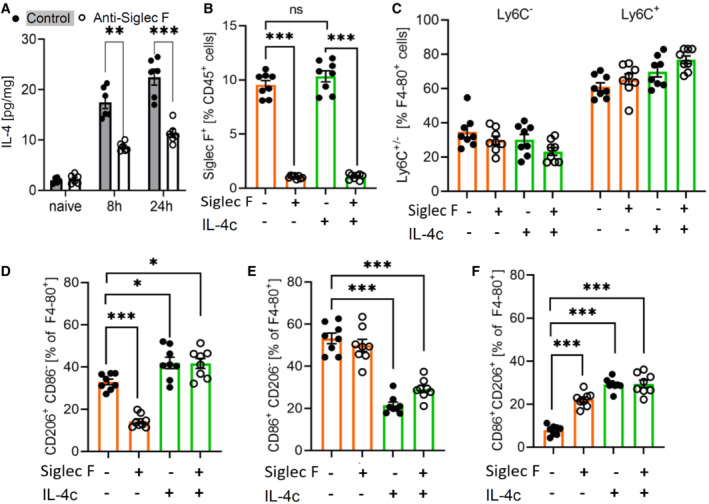
IL‐4 mediates eosinophil effects during resolution of inflammation AIL‐4 levels in paws from naive and zymosan‐injected mice were determined by ELISA 8 and 24 h after injection of zymosan in paws of control or eosinophil‐depleted mice. Data are shown as mean ± SEM (*n* = 6 mice). Student's *t*‐test/two‐way ANOVA/Bonferroni, control vs. anti‐Siglec F antibody treatment: **P < 0.01, ***P < 0.001.BFACS analysis of the eosinophil number in paws from control and eosinophil‐depleted mice with or without IL‐4c treatment 24 h prior zymosan injection. Data are shown as the mean ± SEM (*n* = 8 mice). Two‐way ANOVA/Bonferroni ***P < 0.001.CSame as panel B except that resident (Ly6C^−^) and monocyte‐derived macrophages (Ly6C^+^) were determined.D–FSame as panel B except that M2‐like (panel D), M1‐like (panel E) and M0 macrophages (panel F) were determined. Data are shown as mean ± SEM (*n* = 8 mice). Student's *t*‐test/Two‐way ANOVA/Bonferroni, control vs. anti‐Siglec F antibody treatment: *P < 0.05, ***P < 0.001. Source data are available online for this figure.

Since IL‐4‐expressing eosinophils were cellular neighbors of M2‐like macrophages, we hypothesized that eosinophil‐derived IL‐4 is necessary for correct polarization of M2‐like macrophages. In support of this hypothesis, eosinophil depletion decreased the total IL‐4 levels in the inflamed paws 8 and 24 h after zymosan injection (Fig 6A). To determine whether the altered resolution of inflammation induced by eosinophil depletion can be rescued by IL‐4 application, we administered a stabilized form of IL‐4 (IL‐4c) (Finkelman *et al*, 1993; Milner *et al*, 2010; Jenkins *et al*, 2011). Administration of IL‐4c did neither affect recruitment of eosinophils (Fig 6B) nor the number of resident (Ly6C^−^/CD45^+^/F4‐80^+^) or monocyte‐derived macrophages (Ly6C^+^/CD45^+^/F4‐80^+^) (Fig 6C). However, eosinophil depletion and IL‐4c administration altered macrophage polarization in several ways. First, as seen in the MELC analysis (Fig 5E), eosinophil depletion decreased the number of M2‐like macrophages, which was rescued by IL‐4c administration (Fig 6D). It should be noted that IL‐4c administration to nondepleted mice induced a slight, but significant increase of macrophage polarization toward M2‐like macrophages, which is in line with the known M2‐like polarizing effect of IL‐4 (Fig 6D). Second, the number of M1‐like macrophages was not altered by eosinophil depletion, but decreased in mice receiving IL‐4c (Fig 6E). This decrease could be expected, since IL‐4 counters polarization towards M1‐like phenotypes. Finally, the number of CD86^+^/CD206^+^ M0‐macrophages was increased after eosinophil depletion (Fig 6F) reflecting the compromised M2‐polarization due to decreased IL‐4 levels. Administration of IL‐4c to nondepleted mice increased the number of M0‐macrophages to levels equal to eosinophil‐depleted mice without further affecting their number in eosinophil‐depleted mice (Fig 6F). Thus, eosinophil‐depleted mice show a shift from M2‐like macrophages toward M0 macrophages, while IL‐4c‐induced a shift from M1‐like macrophages toward M0 and M2‐like macrophages, thereby compensating the effects induced by eosinophil depletion.

### Eosinophil‐derived IL‐4 mediates neutrophil recruitment and efferocytosis

**Figure 7:**
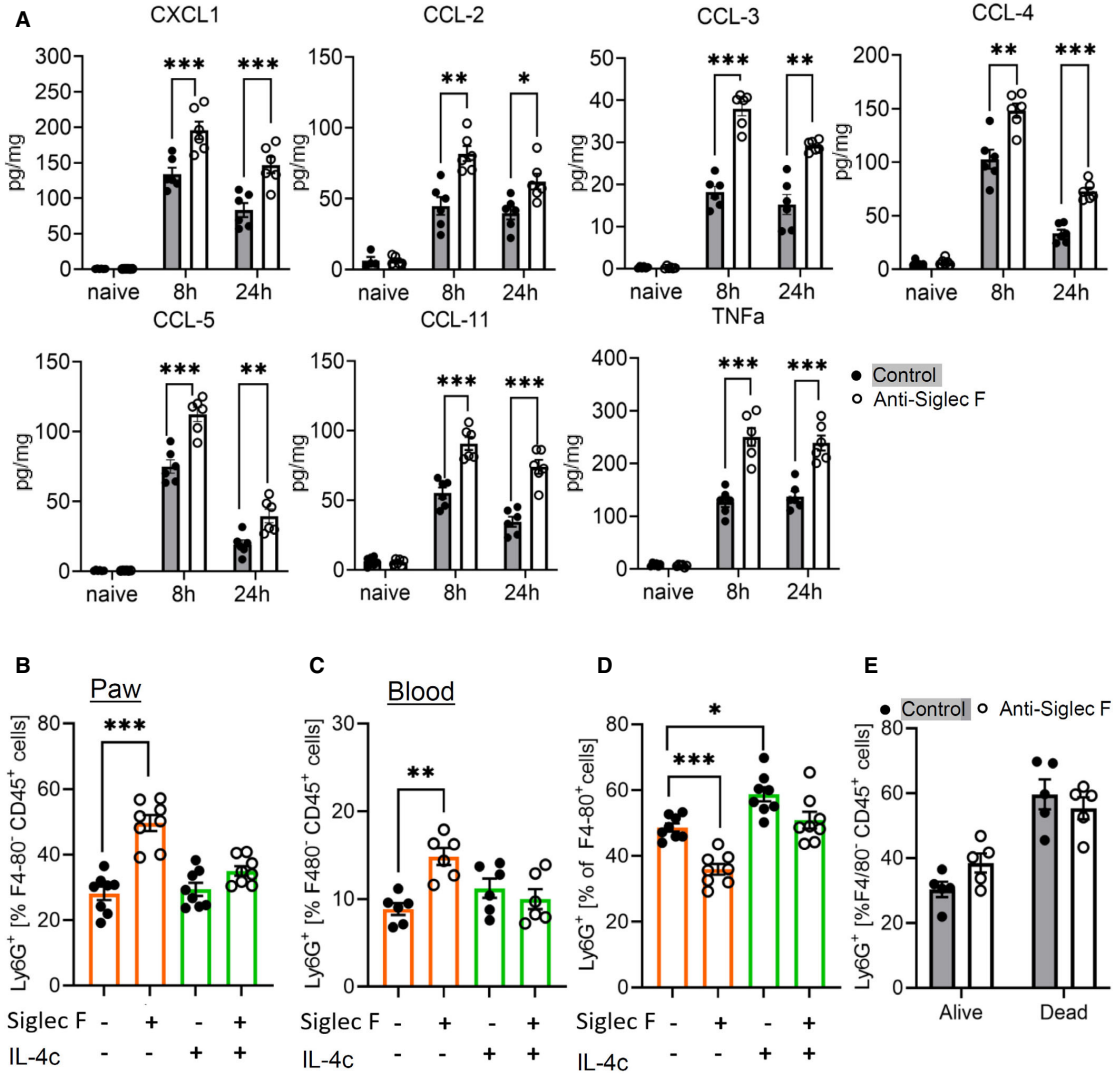
Eosinophil depletion increases neutrophil recruitment and lowers efferocytosis AConcentrations of cytokines and chemokines were determined by multiplex cytokine assay 8 and 24 h after injection of zymosan in paws from control or eosinophil‐depleted mice. Data are mean ± SEM (*n* = 6 mice). Student's one‐way ANOVA, significance between control and anti‐Siglec F antibody treatment *P < 0.05; **P < 0.01; ***P < 0.001.B, CFACS analysis of the neutrophil count in paws (panel B) and peripheral blood (panel C) from control or eosinophil‐depleted mice with or without IL‐4c treatment. Data are shown as the mean ± SEM (*n* = 6–8). Two‐way ANOVA/Bonferroni, **P < 0.01, ***P < 0.001.DSame as panel B except that efferocytosis was determined. *P < 0.05; ***P < 0.001.EFACS analysis of alive and dying neutrophils in paws from control or eosinophil‐depleted mice. Data are shown as the mean ± SEM (*n* = 5 mice). Source data are available online for this figure.

Since MELC analyses shows an increased neutrophil recruitment in eosinophil‐depleted mice (Fig 5I), we determined the levels of neutrophil‐attractant mediators in inflamed paws of eosinophil‐depleted mice. Screening of 26 chemokines, cytokines, and growth factors showed 9 mediators in the inflamed paw 24 h after zymosan injection. Eosinophil depletion significantly upregulated TNFα and the neutrophil‐attractant chemokines CXCL1, CCL‐2, CCL‐3, CCL‐4, CCL‐5, and CCL‐11 (Fig 7A), whereas IL‐1α and IL‐12 were not altered (Appendix Fig S6). FACS analysis confirmed the increased neutrophil numbers in eosinophil‐depleted mice and showed that IL‐4c application is able to reverse this increase of the number of neutrophils (Fig 7B). Notably, neutrophil levels were also increased in the blood of eosinophil‐depleted mice, which normalized after IL‐4c administration (Fig 7C). This is in accordance with a previous report showing that IL‐4 suppresses neutrophil egress from the bone marrow (Panda *et al*, 2020). Eosinophil depletion was sufficient to increase neutrophil numbers also in the blood of mice without inflammation suggesting that eosinophils contribute to the IL‐4‐mediated homeostasis of neutrophil egress (Appendix Fig S7). Next, as predicted by the MELC analyses (Fig 5H), FACS analyses showed a reduced efferocytosis in eosinophil‐depleted mice, which was rescued by IL‐4c administration (Fig 7D). Notably, no change in the survival rate of neutrophils in dependence of eosinophil depletion was seen (Fig 7E) suggesting that the decreased efferocytosis is due to a decreased phagocytotic activity of macrophages.

**Figure 8:**
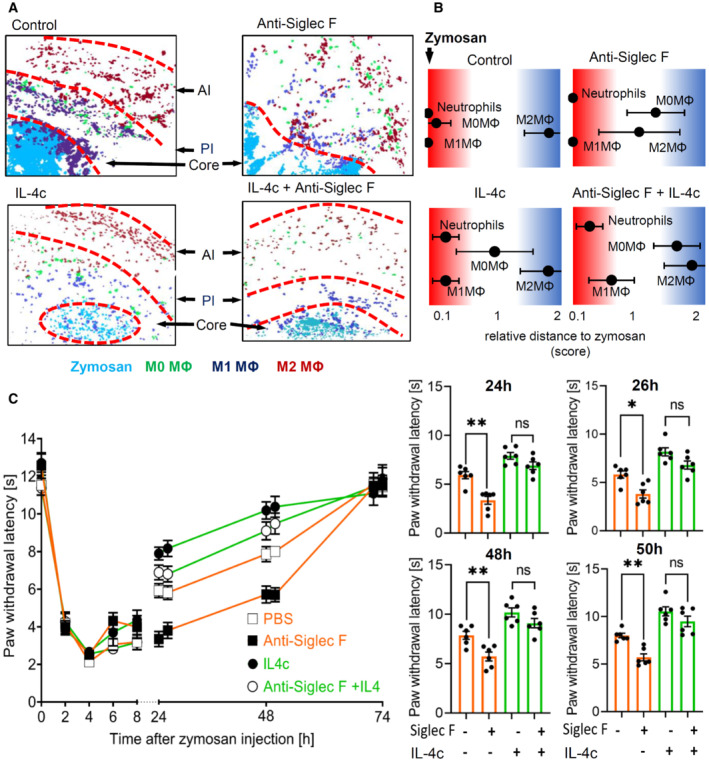
IL‐4 mediates eosinophil effects on the inflammatory structure Composite MELC image showing the disappearance of the pro‐ (PI) and the anti‐inflammatory (AI) regions 24 h after zymosan injection. Shown are representative images of mice receiving anti‐Siglec F or IL‐4c. The red dotted lines depict the area where the transition between the neighboring regions occurs.Relative distance of various immune cell types based on the likelihood for a direct neighborhood of macrophage subtypes in regard to zymosan at the 24 h after zymosan injection. Data are shown as the mean ± SEM (*n* = 5 mice).Zymosan‐induced thermal hypersensitivity in control or eosinophil‐depleted mice with or without IL‐4c treatment 24 h prior zymosan injection. The right panel shows the comparison of paw withdrawal latencies between the 4 treatment groups at the indicated time points. Data are shown as the mean ± SEM (*n* = 6 mice). Two‐way ANOVA/Bonferroni, *P < 0.05, **P < 0.01; ns, not significant. Source data are available online for this figure.

**Figure EV3:**
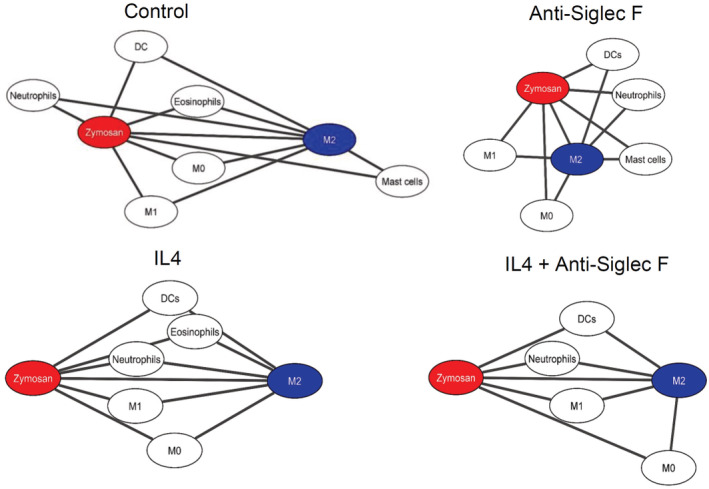
Cellular neighborhood networks of zymosan and M2‐like macrophages Dual centered network analysis of immune cells 24 h after zymosan injection based on the likelihood for a direct neighborhood of macrophage subtypes in regard to zymosan and M2‐like macrophages at the. Data are shown as the mean ± SEM (*n* = 5 mice). Mice were treated with or without anti‐Siglec F antibody and IL4c. The distances between the cells in the visualization represents the statistical probability of being direct neighbors.

Next we studied whether IL‐4c administration can rescue the formation of inflammatory regions in eosinophil‐depleted mice. Analysis of the cellular neighborhood of zymosan showed a similar cell cluster distribution in control and IL‐4c‐treated nondepleted mice (Fig 8A), although IL‐4c administration induced M0 macrophages to shift from a direct to a random neighbor of zymosan. This can be attributed to the overall increased number of M0 macrophages in these mice and as consequence of a more widespread distribution. In eosinophil‐depleted mice, the most important effect is the reversal of the decreased relative distance between zymosan and M2‐like macrophages, after IL‐4c application (Fig 8A). Also the dual‐centered network analysis for zymosan and M2‐like macrophages showed that IL‐4c application prevented the eosinophil depletion‐induced collapse of the neighborhood architecture (Fig EV3). Visualization of cell cluster representing the major immune cell populations confirmed that IL‐4c administration alone did not alter the basic overall structure of the inflammation in regard to the formation of the three inflammatory regions (Fig 8B) but was able to rescue the breakdown of the borders between the PI‐ and AI‐regions after eosinophil depletion (Fig 8B). Finally, IL‐4c treatment removed the differences between control and eosinophil‐depleted mice regarding the thermal paw withdrawal latencies during the resolution phase (24–48 h after zymosan injection; Fig 8C). Thus, we found that eosinophils support the resolution of zymosan‐induced inflammation through IL‐4 by enabling the development of an anti‐inflammatory framework permitting appropriate macrophage polarization, neutrophil recruitment, and efferocytosis.

## Discussion

Here, we identified three major inflammatory areas in our inflammation model based on the macrophage polarization and their cellular neighborhoods, whereby an inflammatory core region including the pathogen is surrounded by a proinflammatory region containing M1‐like macrophages and an outer region containing anti‐inflammatory cells. This regional structure, which starts to form within 8 h beginning with the polarization of M1‐like macrophages in the neighborhood of the pathogen, is later followed by the formation of an outer region defined by M2‐like macrophages. The finding that the pro‐ and anti‐inflammatory regions coexist during an ongoing inflammation shows that the concept of a gradual temporal transition from pro‐ to anti‐inflammation (Sansbury & Spite, 2016; Schett & Neurath, 2018; Serhan & Levy, 2018) needs further refinement in the sense that the temporal transition is reflective of a change in the ratio of the sizes of pro‐ and anti‐inflammatory regions. The finding that the results of the MELC analysis were reproduced in the FACS analysis of total paw tissue, supports, and strengthen the point that the majority of M1‐like and M2‐like macrophages are indeed confounded to the specific inflammatory regions.

While probably a variety of events participate in the formation of the anti‐inflammatory region, one triggering event may be neutrophil apoptosis. Recognition and phagocytosis of apoptotic neutrophils by M1‐like macrophages has been shown to be a key element in the induction of resolution of inflammation (Filep, 2022). Efferocytosis has been demonstrated to induce a metabolic switch in macrophages and their reprogramming from M1‐like to a M2‐like phenotype (Morioka *et al*, 2018), which exhibits release of anti‐inflammatory mediators (i.e. IL‐10, TGF‐β, and prostaglandin E2; Fadok *et al*, 1998; McDonald *et al*, 1999; Huynh *et al*, 2005; Girkontaite *et al*, 2007; Kumaran Satyanarayanan *et al*, 2019). However, also other signals from the inflammatory environment that can induce M2‐like phenotypes in macrophages could be involved in the late appearance of these anti‐inflammatory cells. In this regard, metabolic changes (i.e. bioenergetics) in immune cells can occur as response to external signals received either from other cells, such as signals and antigens activating pattern recognition receptors (PRRs) and cytokine receptors, or from changes in their microenvironment, including nutrient or oxygen availability (Tannahill *et al*, 2013), influencing transcriptional and posttranscriptional events in macrophages (O'Neill & Pearce, 2016). Thus, the formation of the AI‐region might be initiated by early polarization events, leading then to the production of anti‐inflammatory mediators by the M2‐like macrophages. These mediators would over the time generate their own local microenvironment if located in a sufficient distance to the core region and the high density of proinflammatory signals in this region (Kern *et al*, 2018). Once this anti‐inflammatory structure (in our model the AI‐region) is established, newly recruited cells polarize according to the region they arrive in thereby reinforcing the character of this specific inflammatory region (Chen *et al*, 2020). The balance between the pro‐ and anti‐inflammatory regions would depend on the amount of pro‐ and anti‐inflammatory mediators generated locally ensuring a flexible response by expansion or shrinkage of either pro‐ or anti‐inflammatory regions.

The observed basic architecture may provide the possibility to predict the role of an immune cell based on its localization within the given inflammatory setting. However, any attempt to make functional predictions based on the localization of an immune cell must take into consideration that several immune cell subtypes/phenotypes exist, which fulfill different and in some cases, for example, M1‐ and M2‐like macrophages, even opposing functions (Murray & Wynn, 2011; Ortega‐Gomez *et al*, 2013). We found that eosinophils were located in all three inflammatory regions, but adapted to its surroundings with an altered cytokine expression, which is in accordance to previous reports of environment‐specific eosinophil subtypes (Andreev *et al*, 2020; Dolitzky *et al*, 2021). Depletion of eosinophils leads to increased neutrophil recruitment, decreased M2‐like macrophage polarization, and decreased efferocytosis as well as the breakdown of the anti‐inflammatory region. IL‐4c administration restored these cellular functions, which is in line with previous publications showing that IL‐4 is involved in the regulation of these three processes. However, since eosinophils are known to generate a multitude of mediators, it seems likely that eosinophil‐derived IL‐4 has a prominent role in mediating the observed phenotypes allowing to compensate for the loss of other potentially involved eosinophil‐derived mediators. Although the net effect of eosinophil function in the zymosan‐induced inflammation is anti‐inflammatory, this net effect does not rule out local proinflammatory functions of eosinophils, that is, in the core region. Thus, the immune cells in question must be carefully examined for subtype‐specific localizations, especially if they are located in regions, which would suggest opposing roles (i.e. core‐ and AI‐region). In this regard, the observed seemingly even distribution of DCs throughout the three inflammatory regions may just be based on the lack of the use of more specialized DC markers.

It should be noted that the observed regional architecture of the zymosan‐induced inflammation does not necessarily represent an architecture found in other inflammation models. The zymosan‐induced inflammation model was chosen, since zymosan can be easily localized after fluorescence labeling (Pierre *et al*, 2017; Kern *et al*, 2018). This circumstance allows the immune response to form and maintain the observed regional structures. It would be expected that a similar inflammatory architecture will be seen in tissues with other pathogen‐driven models for innate inflammation with relative immobile pathogens (e.g. bacteria, yeast). Moreover, it will be interesting to see if inflammation models with mixed immune responses, including components of the innate and the adaptive immune system, reproduce the observed structures or whether they exhibit other distinct inflammatory architectures. Finally, the methodological approach described in our report will allow new insights in therapeutic mechanisms and consequences, that is, immunosuppressants, in regard to immune cell interactions helping to refine therapeutic approaches.

## Materials and Methods

### Mice

Male C57BL/6N mice (6–8 weeks) were purchased from Janvier (Le Genest, France) and treated according to the International Association for the Study of Pain guidelines. For all experiments the ethics guidelines for investigations in conscious animals were obeyed and the procedures were approved by the local ethics committee (Regierungspräsidium Darmstadt). The animals had free access to food and water and were maintained in climate‐ (23 ± 0.5°C) and light‐controlled rooms (light from 6.00 a.m. to 6.00 p.m.).

Inflammation was induced by injection of 10 μl zymosan (3 mg/ml in PBS; Merck, Darmstadt, Germany) subcutaneously into the plantar side of one hind paw. Eosinophil depletion was achieved by intraperitoneal (i.p.) injection of anti‐Siglec F antibody (0.883 mg/kg; clone 238,047; R&D Systems, Minneapolis, MN) 24 h prior zymosan injection. As control purified rat IgG2a (Biolegend, San Diego, USA) was used. IL‐4c was prepared using IL‐4 (Peprotech, Hamburg, Germany) and anti‐IL4 antibody (Biolegend, San Diego, USA) (Finkelman *et al*, 1993; Milner *et al*, 2010; Jenkins *et al*, 2011) and administered i.p. (0.166 mg/kg IL‐4 and 0.883 mg/kg anti‐IL4 antibody) 24 h prior zymosan injection. (Finkelman *et al*, 1993; Milner *et al*, 2010; Jenkins *et al*, 2011).

### Multiepitope‐ligand‐carthographie

Multiepitope‐ligand‐carthographie is an automated immunohistological imaging method that can be used to visualize high numbers of antibodies on the same sample (Pierre & Scholich, 2010; Pierre *et al*, 2017; Kornstädt *et al*, 2020). Briefly, paw tissue sections were taken at 10 μm thickness on silanized cover slips, fixed in 4% paraformaldehyde in PBS for 15 min, permeabilized with 0.1% Triton X100 in PBS for 15 min, and blocked with 3% BSA in PBS for 1 h. The tissue sample was placed on the stage of a Leica DM‐IRE2 and a picture was taken. Then, in an automated procedure, the sample was incubated with bleachable fluorescence‐labeled antibodies and washed with PBS. Afterward, phase‐contrast and fluorescence signals were imaged by an Apogee‐KX4 camera (Apogee Instruments, Logan, UT). A bleaching step was performed to delete fluorescence signals and the post‐bleaching image was recorded. Then the next antibody was applied and the process was repeated. For data analysis, fluorescence images produced by each antibody were aligned pixel‐wise and corrected for illumination faults using flat‐field correction. The post‐bleaching images were subtracted from their following fluorescence image. The antibodies used are listed in Appendix Table S1.

### Image analysis

First, all grayscale antibody channel images were processed using ImageJ v1.52 (NIH, Bethesda, MD, USA) to diminish noise, background fluorescence, and remove artifacts for further analyses if necessary. Then Cell Profiler (v3.1.9) (McQuin *et al*, 2018) was used for additional illumination correction and the generation of a cell mask for single‐cell segmentation using the images for propidiumiodide (cell nuclei) and CD45. The segmentation mask was imported in histoCAT (v1.76) (20) with the corresponding antibody channel images. All images, excluding the images used for single‐cell mask generation, were z‐score normalized and used for Barnes‐Hut t‐SNE (BH t‐SNE) (Amir *et al*, 2013) and PhenoGraph analysis (Levine *et al*, 2015) as implemented in histoCAT. PhenoGraph defines cell clusters based on single‐cell mask and marker colocalization (k set to 20 or 30). BH t‐SNE scatter plot was overlaid with a colored PhenoGraph cluster map. PhenoGraph clusters were determined on their marker expression and classified as different cell types for quantification of cells in the images. The number of objects per cluster was normalized to the total number of objects in the cell mask to calculate the relative number of cells per cell type. PhenoGraph clusters were exported as CSV file and further analyzed with the SPADE (v3.0) tool for Matlab to generate Spanning‐trees of density‐normalized events using standard conditions (Qiu *et al*, 2011). After defining single cells with the segmentation mask in histoCAT the z‐score normalized images were exported and used for a FACS‐like analysis in FlowJo (v10.8.1). To investigate the relationship between clusters, neighborhood analysis was performed under standard conditions as implemented in histoCAT (Schapiro *et al*, 2017). Here, pairwise interactions between cell phenotypes are calculated for each cell and its neighbors at a user‐defined distance (4 pixels) and compared to a randomized version of the cell distribution. The permutation test provides either a significant (*P* < 0.05) interaction, avoidance, or no likelihood at all between cell phenotypes. The gained result was a score between 0 and 100 for all clusters which was imported in Cytoscape (v3.8.2) to generate neighborhood networks showing relative distances to the defined centers (Otasek *et al*, 2019).

### FACS analysis

Tissue preparation for polychromatic flow cytometry was performed as described previously (Suo *et al*, 2014). Single‐cell suspensions were incubated with 2% Fc‐blocking reagent (Mouse BD Fc Block; BD Pharmingen, NJ, USA) in PBS (10 min, 4°C), followed by incubation with an antibody cocktail (Appendix Table S1) for 30 min at 4°C. Samples were acquired with a FACS Canto II flow cytometer and analyzed using FlowJo software v10 (both BD Biosciences, Heidelberg, Germany). For gating, fluorescence minus 1 (FMO) controls were used.

### Behavioral tests and edema measurement

Mechanical hypersensitivity was determined by measuring paw withdrawal latency using a plantar aesthesiometer (Dynamic Plantar Aesthesiometer, Ugo Basile). A force range of 0 to 5 g with a ramp of 0.5 g/s was applied with a steel rod of 2 mm in diameter, until a strong and immediate withdrawal occurred. The cutoff time was set to 20 s. Thermal hypersensitivity was determined using the Hargreaves test as described previously (Hohmann *et al*, 2017). Paw withdrawal latency was determined by increasing heat at the mid‐plantar region of a paw at the same rate for each trial (up to 12% starting at 32°C) using an IITC Plantar Analgesia Meter (Hargreaves Test; IITC Life Science, Woodland Hills, CA, USA) with a cutoff time of 20 s. Baseline measurements were performed on two consecutive days before zymosan injection.

The size of paw edemas was determined at the indicated times after injection of zymosan in one hind paw. A plethysmometer (IITC Life Science, Woodland Hills, CA, USA) was used to quantify the edema volume by immersion of the mouse paw as described previously (Tarighi *et al*, 2019).

### Cytokine measurements

Cytokine and chemokine levels in paws of mice were determined using the Bio‐Plex Pro mouse cytokine group I kit (Bio‐Rad Feldkirchen, Germany). The tissue was lysed in 400 μl lysis buffer with 1x Protease Inhibitor Cocktail (Roche, Mannheim, Germany) in Tissue Extraction Reagent (Thermo Fisher Sciemtific, Waltham, MS). Samples were cut in small pieces and then sonicated twice at 60% power for 10 s with an Ultrasonic Homogenizer (SONOPULS HD2070 MS73, Bandelin, Berlin, Germany). Afterward, all samples were centrifuged for 10 min at 10,000 *g* and the supernatant harvested. The concentration of total protein in the samples was assessed by the bicinchoninic acid assay. All samples were diluted to a final protein concentration of 200–900 μg/ml, according to the kit requirements. IL‐1α, IL‐1β, IL‐2, IL‐3, IL‐4, IL‐5, IL‐6, IL‐9, IL‐10, IL‐12p40, IL12‐p70, IL‐13, IL‐17, Eotaxin, G‐CSF, GM‐CSF, IFN‐γ, CXCL1, CCL2, CCL3, CCL4, CCL5, and TNF‐α levels were determined using a Bioplex‐200 (Bio‐Rad, Feldkirchen, Germany) according to the manufacturer's protocol. IL‐4 was also measured using the Mouse IL‐4 Quantikine ELISA Kit (R&D Systems, Minneapolis, MN) according to the manufacturer's protocol.

### Data analysis and statistics

Determination of statistically significant difference in all experiments was conducted with One‐ or Two‐way analysis of variance (ANOVA) followed by *post hoc* Bonferroni‐correction. Comparison of two groups was performd by Student's *t‐*test with Welch's correction. In all animal experiments, the mice were randomized, and the researcher was blinded in behavioral experiments edema measurements. All data show biological replicates.

## Author contributions

**Anja Kolbinger:** Investigation; visualization; writing—review and editing. **Tim J Schäufele:** Investigation; writing—review and editing. **Hanna Steigerwald:** Investigation; writing—review and editing. **Joschua Friedel:** Investigation; writing—review and editing. **Sandra Pierre:** Resources; investigation; writing—review and editing. **Gerd Geisslinger:** Resources; supervision; funding acquisition; writing—review and editing. **Klaus Scholich:** Conceptualization; resources; supervision; funding acquisition; writing—original draft; project administration; writing—review and editing.

## Disclosure and competing interests statement

The authors declare that they have no conflict of interest.

**The paper explained**

**Problem**

An inflammation comprises numerous immune cells, which are positioned related to their function thereby allowing the appropriate interactions with other immune cells. Identification of the relevant immune cell interactions *in vivo* is crucial for successful interventions in immunological processes, but are normally rudimentary due to the limited numbers of markers, which can be visualized.

**Results**

Using multiplex immunohistology we identified cellular neighborhoods of the immune cell subtypes involved in a localized TLR‐type 2 inflammation. The pathogen‐containing region bordered to a proinflammatory region, which was enclosed by an antiinflammatory region. This structure remained steady throughout the course of the inflammation. Eosinophil‐derived IL‐4 was essential for forming the regions and correct resolution of inflammation.

**Impact**

This study shows that a pathogen‐driven inflammation is based on pro‐ and anti‐inflammatory processes, which are spatially, but not temporally, separated. The identification of the internal architecture of an inflammation allows the prediction of immune cell functions and interactions based on their localization providing new possibilities to develop and assess therapeutic interventions.

## For more information

- HistoCat: https://www.bodenmillerlab.com/#/
- Cell profiler: https://cellprofiler.org/

## Supporting information



Appendix

Expanded View Figures PDF

Source Data for Expanded View and Appendix

PDF+

Source Data for Figure 1

Source Data for Figure 2

Source Data for Figure 3

Source Data for Figure 4

Source Data for Figure 5

Source Data for Figure 6

Source Data for Figure 7

Source Data for Figure 8

## Data Availability

This study includes no data deposited in external repositories.
