# Supplementary material for: Eosinophil‐derived IL‐4 is necessary to establish the inflammatory structure in innate inflammation
Source: EMBO Mol Med. 2022 Dec 21;15(2):e16796. doi: 10.15252/emmm.202216796 (PMC9906331; doi:10.15252/emmm.202216796)
Supplement: Supplementary file 1 — Appendix [file EMMM-15-e16796-s002.pdf]

Appendix table and figures

Table S1:	Antibodies used for MELC and FACS analysis
Fig. S2:	MELC-based bioinformatic analysis of macrophage polarization
Fig. S3:	Gating strategies for FACS analysis
Fig. S4:	MELC-based neighborhood analysis of zymosan and macrophage subpopulations
Fig. S5:	MELC analysis of the distribution of dendritic cells and eosinophils
Fig. S6:	The anti Siglec F-depletion antibody does not interfere with binding of the eosinophil detection antibody
Fig. S7:	IL1a and IL6 levels after eosinophil-depletion
Fig. S8:	Neutrophil numbers in blood after eosinophil-depletion in naive mice

Appendix Table S1:

Table ST1: Antibodies used for MELC and FACS analysis

Target	Company	Clone number	Order number
CD3	Miltenyi Biotec	REAG1	130-109-838
CD4	Southern Biotech	L3T4	1540-02
CD8	BD Pharmingen	53-6.7	553030
CD11b	BioRad	M1/70.15	MCA74F
CD11c	Miltenyi Biotec	N418	130-102-799
CD19	BDPharmingen	1D3	557398
CD22	Miltenyi Biotec	Cy34.1	130-102-576
CD25	Miltenyi Biotec	7D4	130-102-550
CD29	Miltenyi Biotec	HMβ1-1	130-102-557
CD31	BD Biosciences	MEC13.3	553373
CD41	AbDSerotec	MWReg30	MCA2245F
CD45	Miltenyi Biotec	30F11.1	130-116-535
CD54	Biolegend	YN1/1.7.4	116105
CD80	Biolegend	16-10A1	104706
CD86	Biolegend	GL-1	105002
CD117	Bioss		bs-10005R-Cy5
CD127	eBiosciences	A7R34	11-1271-82
CD206	Biolegend	C068C2	141708
CD209	eBiosciences	LWC06	11-2092-80
Cytokeratin	eBiosciences	AE1/AE3	53-9003-82
CD335	Invitrogen	29A1.4	11-3351-82
F4-80	Biolegend	BM8	123107
GATA3	Santa Cruz	HG3-31	sc-268
IFNg	eBioscience	XMG1.2	12-7311-41
IL1b	Thermo Fisher	NJTEN3	11-7114-82
IL-4	Biolegend	11b11	504109
IL-6	eBioscience	MP5-20F3	11-7061-82
IL-10	eBioscience	JES5-16E3	11-7101-41
IL13	invitrogen	eBio13A	53-7133-82
IL-33	R&D	396118	IC3626P
Ki67	eBioscience	SolA15	11-5698-80
Ly6C	eBioscience	HK 1.4	17-5932-80
Ly6G	eBioscience	RB6-8C5	RM3005
MHC II	Miltenyi Biotec	REA813	130-112-233
NK1.1	BDPharmingen	PK136	561046
Propidium Iodide	Sigma		P4170
RORgt	eBioscience	B2D	12-6981-80
Sca1(Ly-6A/E)	Biolegend	E13-161.7	122511
Siglec F	BD Bioscience	E50-2440	552126
TNFa	Miltenyi	REA636	130-119-561
**Antibodies used only for FACSAnalysis**			
CD45	Miltenyi Biotec	REA737	130-110-665
Siglec F	Miltenyi Biotec	ES22-10D8	130-102-167
Ly6G	Biolegend	1A8	127624

Appendix Fig. S2

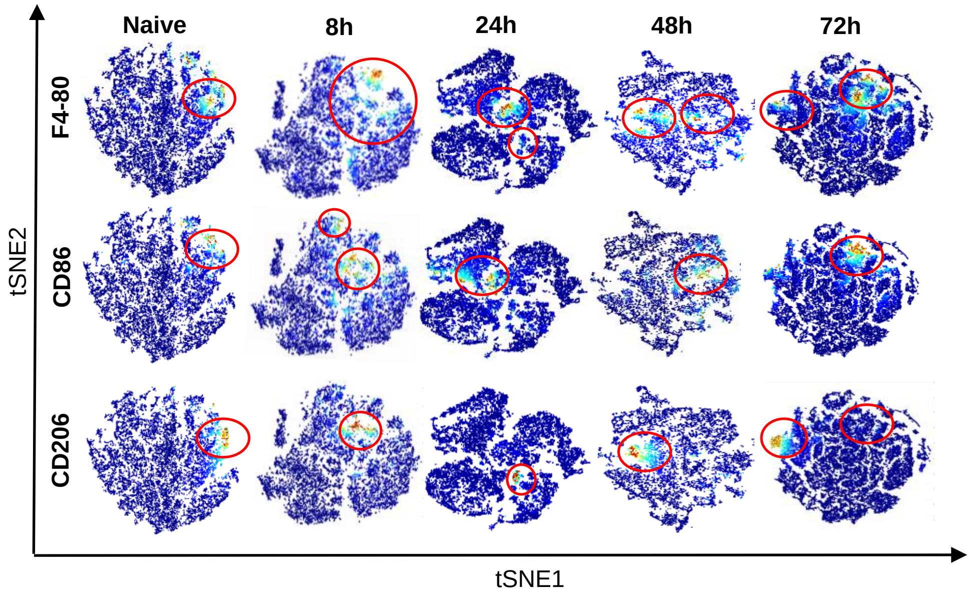

Fig. S2: MELC-based bioinformatic analysis of macrophage polarization. tSNE analysis of macrophage polarization based on M1-like (CD86) and M2-like (CD206) markers at various time points after zymosan injection in one hind paw

Appendix Fig. S3

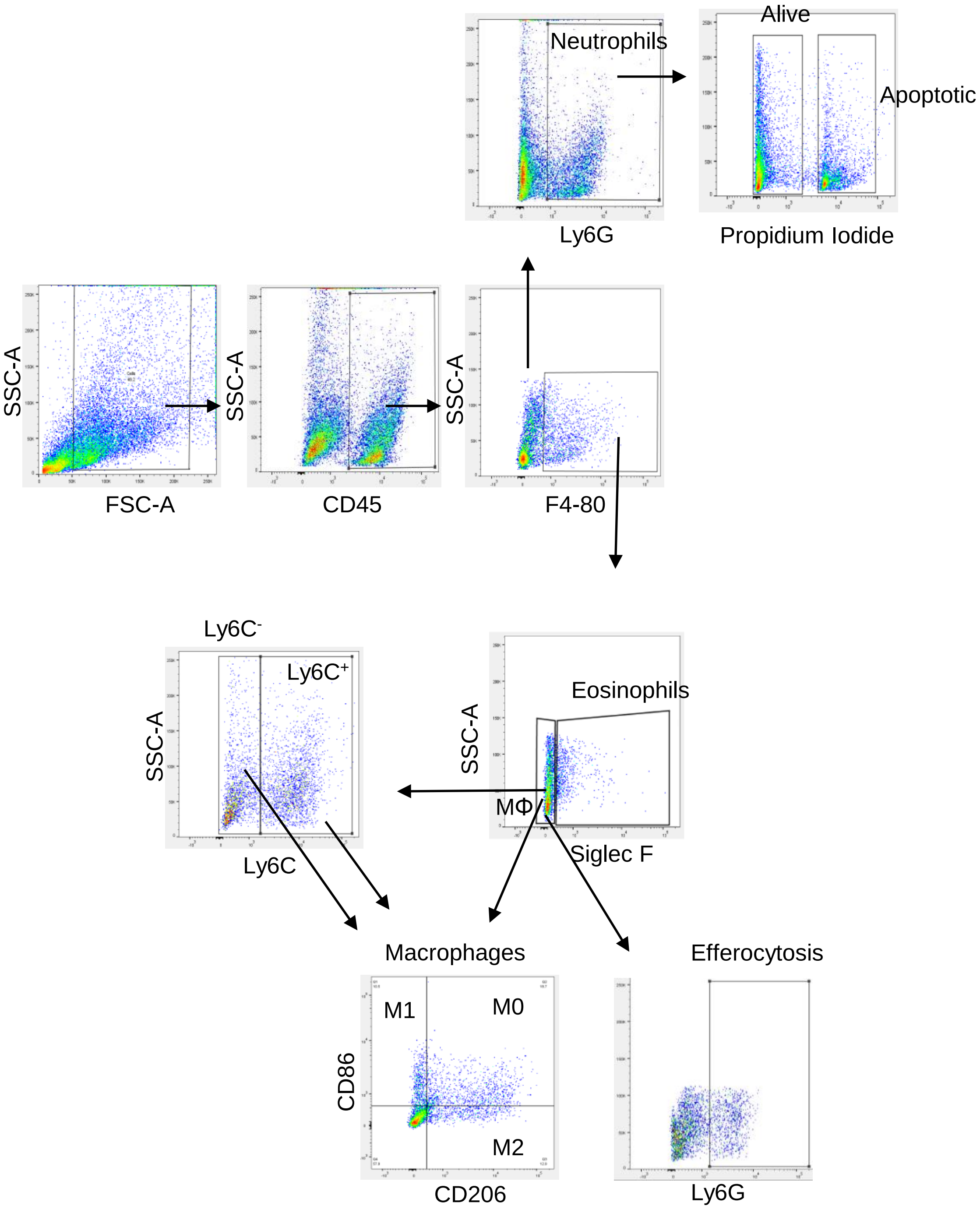

Fig. S3: Gating strategy for immune cells and their subtypes in paws after injection of zymosan (10 µl, 3 mg/ml). Cells were gated based on their side scatter area (SSC-A) and forward scatter area (FSC-A). Afterwards $CD45^+$ immune cells were identified. Within F4-80^+ cells Siglec F^- cells were identified as macrophages and Siglec F^+ cells as eosinophils. F4-80^+ Siglec F^- macrophages were also further analysed for in resident ($Ly6C^-$) and monocyte-derived macrophages ($Ly6C^+$). M1-like macrophages were defined as $CD86^+/CD206^-$ cells, M2-like macrophages as $CD86^-/CD206^+$ cells and double positive macrophages as M0 macrophages.
$Ly6G^+$/F4 80^- cells were identified as neutrophils. The neutrophils were divided in dead cells positive for propidium iodide and alive cells negative for propidium iodide.

Appendix Fig. S4

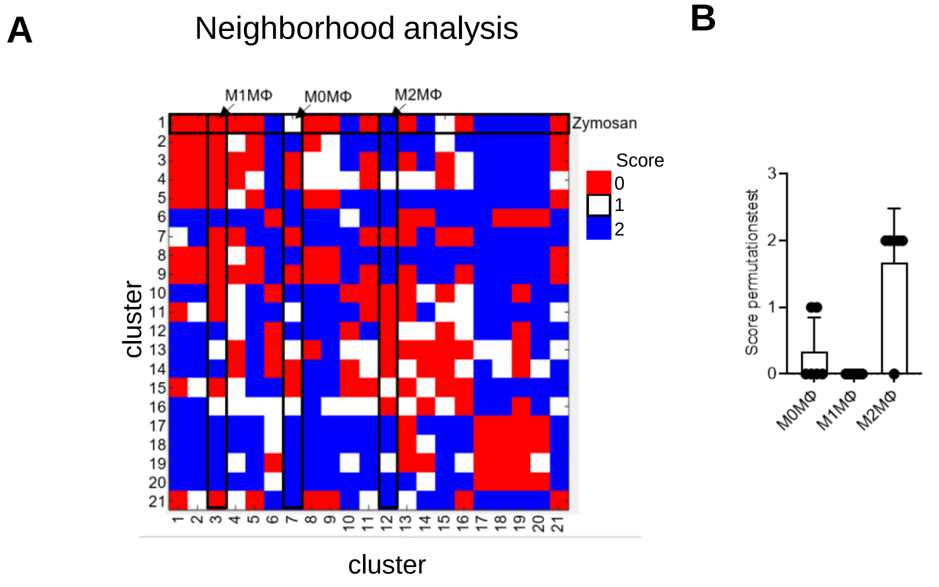

Fig. S4: MELC-based neighborhood analysis of zymosan and macrophage subpopulations. (A) Representative analysis of the zymosan neighborhood in inflamed paws 24 h after zymosan injection. Score 0 = neighbors, Score 1 = random distribution, score 2 = no neighbors. **(B)** Score distribution of macrorphage populations determined for the zymosan neighborhood in individual mice 24 h after zymosan injection.

Appendix Fig. S5

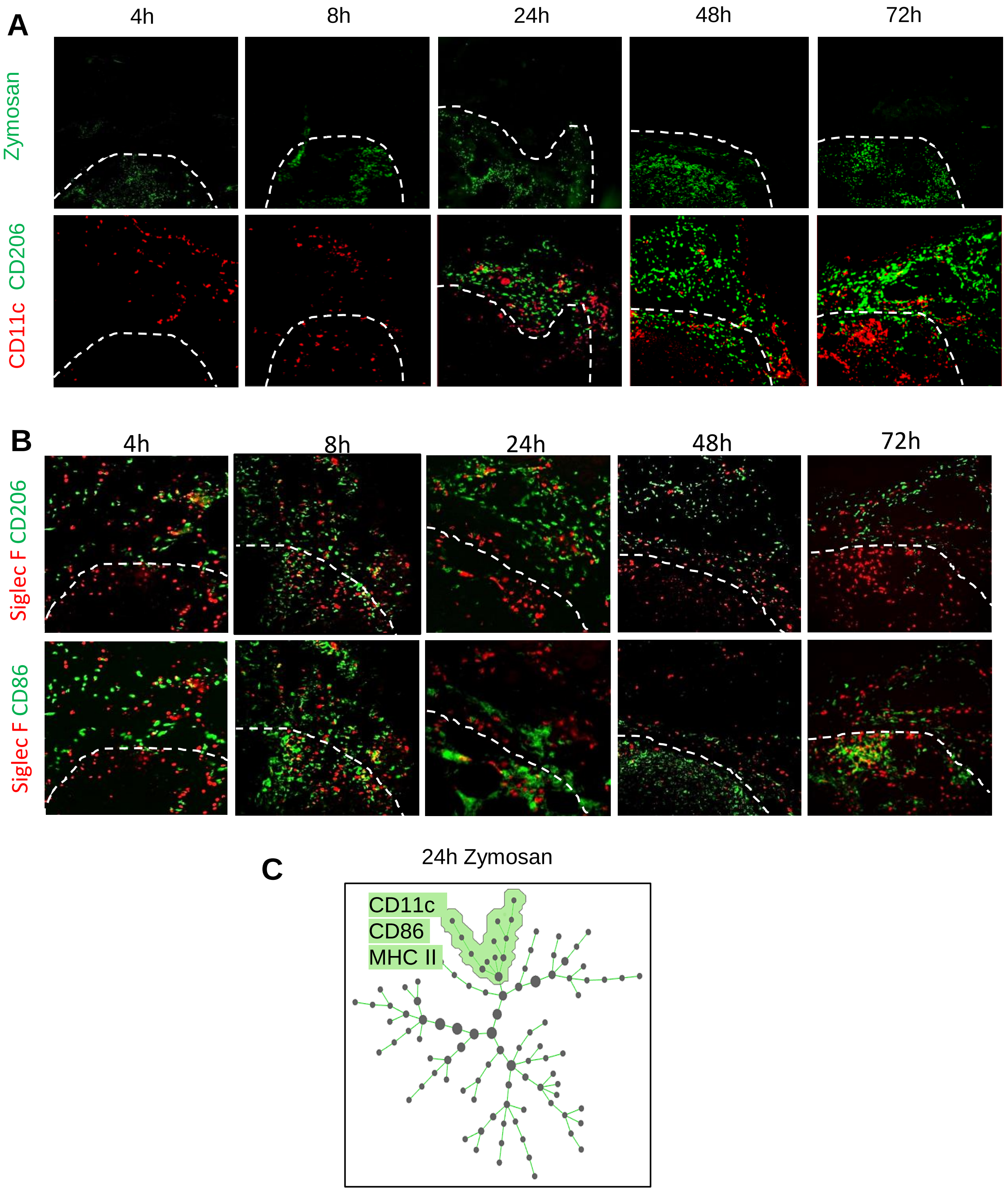

Fig. S5: MELC analysis for the distribution and dendritic cells and eosinophils. (A, B) Representative MELC images showing the distribution of DCs (panel A) and eosinophils (panel B) in the inflamed paw at different time points after zymosan injection**.** The white dotted lines depict the border of the core region. **(C)** SPADE analysis of the distribution of CD11c-expressing clusters 24 hours after zymosan injection in one hind paw.

Appendix Fig. S6

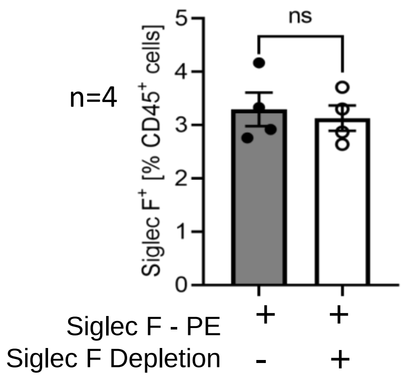

Fig. S6: The anti Siglec F-depletion antibody does not interfere with binding of the eosinophil detection antibody. Blood cells were incubated either with the anti-Siglec F FACS antibody (clone ES22-10D8, Miltenyi Biotech) alone or together with equal amounts of the depletion antibody (clone 238047, R&D Systems). Data are mean ± S.E.M. (n=4). Students T-test was used. No significance was detected.

Appendix Fig. S7

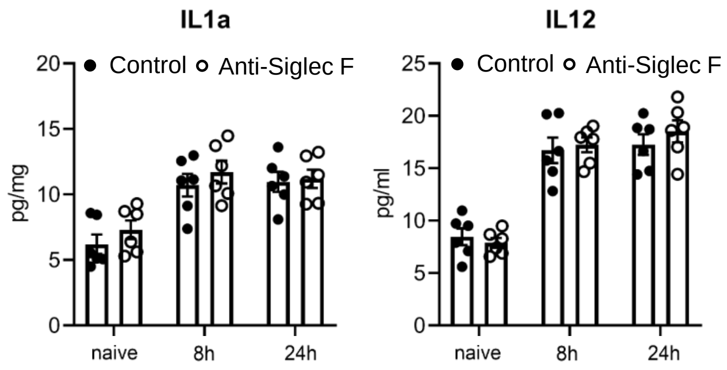

Fig. S7: Eosinophil-depletion does not alter IL1a and IL12 levels in inflamed hind paws. Concentrations of cytokines and chemokines were determined by multiplex cytokine assay 8 and 24 h after injection of zymosan in paws from control or eosinophil-depleted mice. Data are mean ± S.E.M. (n=6). Two-way ANOVA. No significance was detected.

Appendix Fig. S8

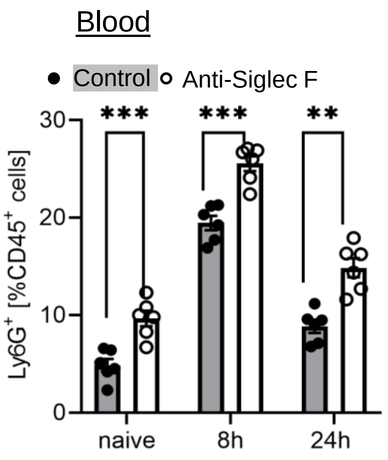

Fig. S8: FACS analysis of neutrophils in blood. FACS analysis of the neutrophil count in peripheral blood from control or eosinophil-depleted mice in naïve animals and 8 or 24 hours after zymosan injection. Data are shown as the mean ± S.E.M. (n=6). Two-way ANOVA/Bonferroni, **p< 0.01, ***p< 0.001.
