# Supplementary material for: Eosinophil‐derived IL‐4 is necessary to establish the inflammatory structure in innate inflammation
Source: EMBO Mol Med. 2022 Dec 21;15(2):e16796. doi: 10.15252/emmm.202216796 (PMC9906331; doi:10.15252/emmm.202216796)
Supplement: Supplementary file 2 — Expanded View Figures PDF [file EMMM-15-e16796-s007.pdf]

Expanded View Figures

Figure EV1. Workflow of the bioinformatic analysis for the MELC images.

© 2022 The Authors

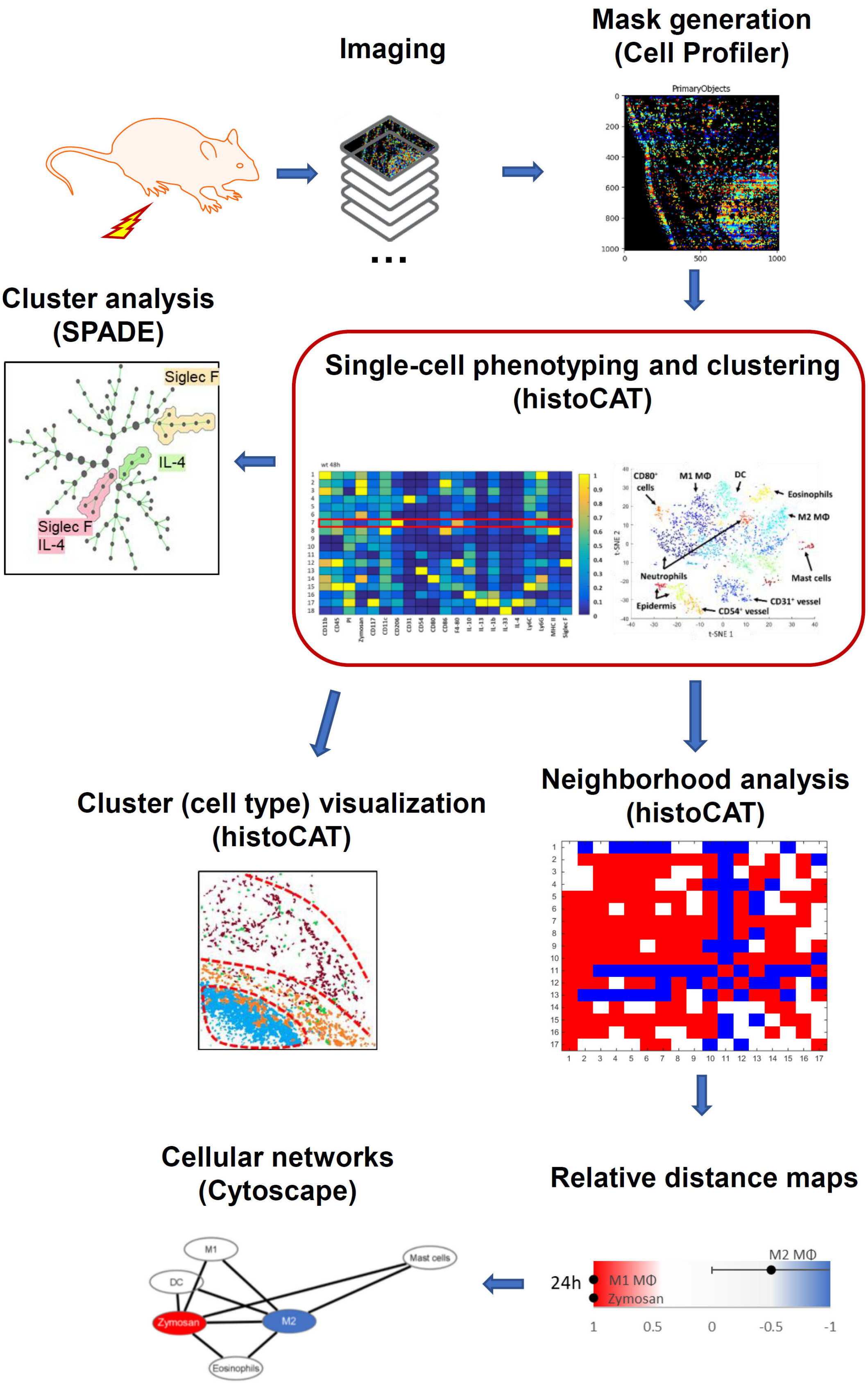
Imaging
Mask generation
(Cell Profiler)
PrimaryObjects
Cluster analysis
(SPADE)
Siglec F
IL-4
Siglec F
IL-4
Single-cell phenotyping and clustering
(histoCAT)
CD80+ cells
M1 MΦ
DC
Eosinophils
M2 MΦ
Neutrophils
Mast cells
Epidermis
CD31+ vessel
CD54+ vessel
t-SNE 1
t-SNE 2
Cluster (cell type) visualization
(histoCAT)
Neighborhood analysis
(histoCAT)
Cellular networks
(Cytoscape)
Relative distance maps
24h
M1 MΦ
Zymosan
M2 MΦ
M1
DC
Mast cells
Zymosan
M2
Eosinophils

Figure EV1.

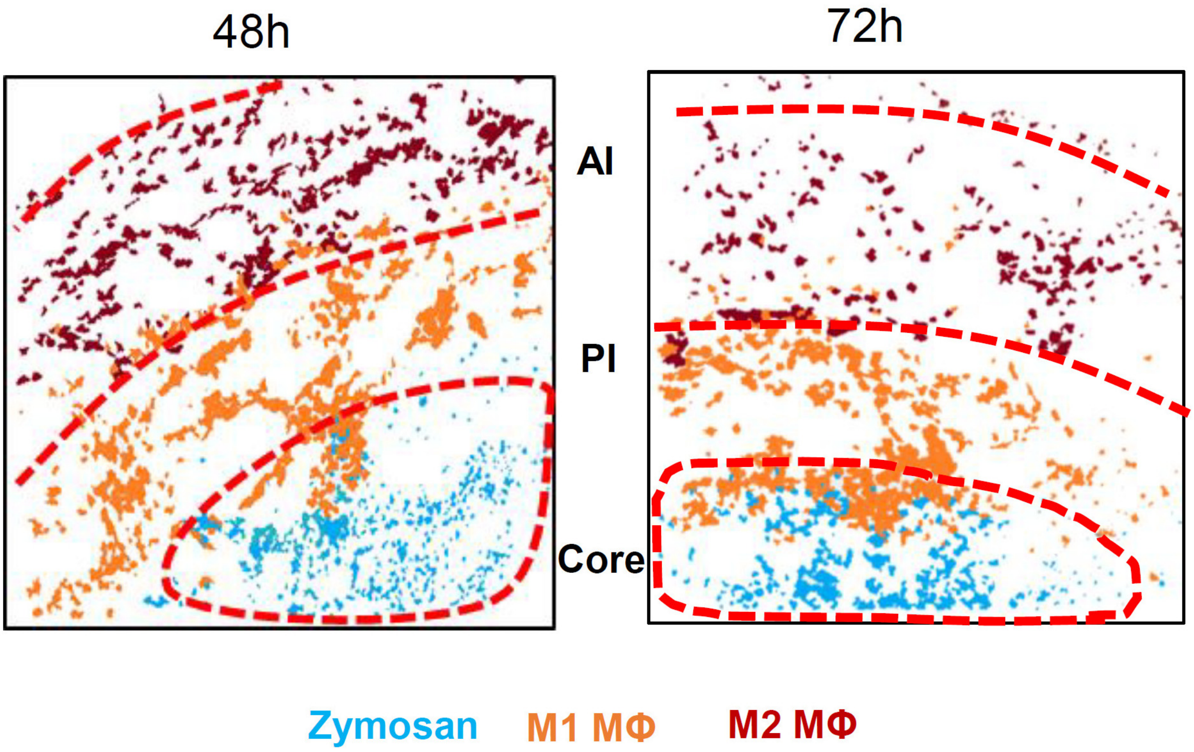

Figure EV2. Distribution of macrophage subpopulation 48 and 72 h after zymosan injection.

Composite MELC images 24 and 72 h after zymosan injection showing the position of the core-, PI- and the AI-regions in the tissue in regard to the localization of M1- and M2-like macrophages (MΦ) and zymosan. The red dotted lines depict the area where the transition between the neighboring regions occurs.

 © 2022 The Authors

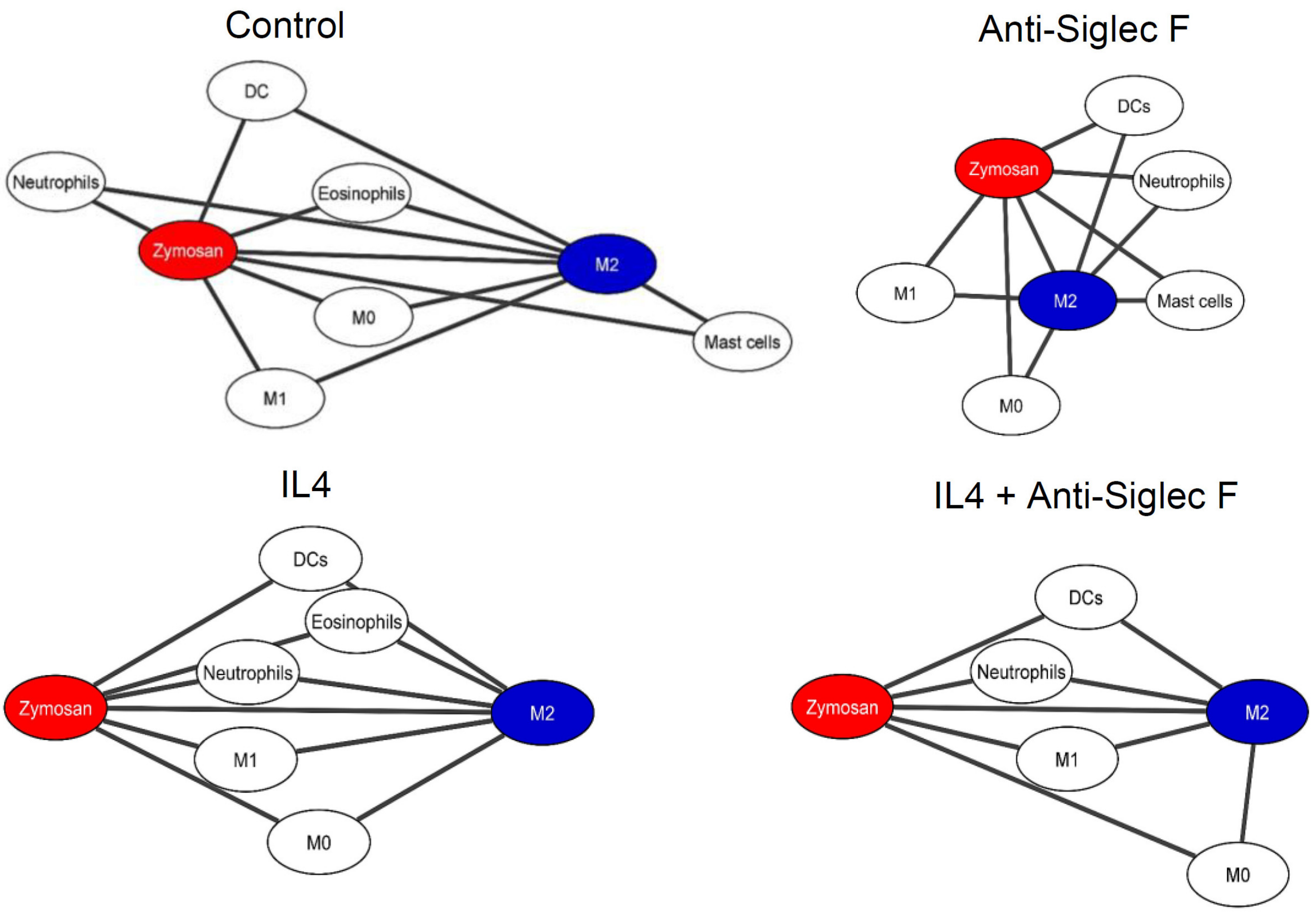

Figure EV3. Cellular neighborhood networks of zymosan and M2-like macrophages.

Dual centered network analysis of immune cells 24 h after zymosan injection based on the likelihood for a direct neighborhood of macrophage subtypes in regard to zymosan and M2-like macrophages at the. Data are shown as the mean ± SEM ($n = 5$ mice). Mice were treated with or without anti-Siglec F antibody and IL4c. The distances between the cells in the visualization represents the statistical probability of being direct neighbors.
