# Supplementary figures and images for: Eosinophil‐derived IL‐4 is necessary to establish the inflammatory structure in innate inflammation
Source: EMBO Mol Med. 2022 Dec 21;15(2):e16796. doi: 10.15252/emmm.202216796 (PMC9906331; doi:10.15252/emmm.202216796)

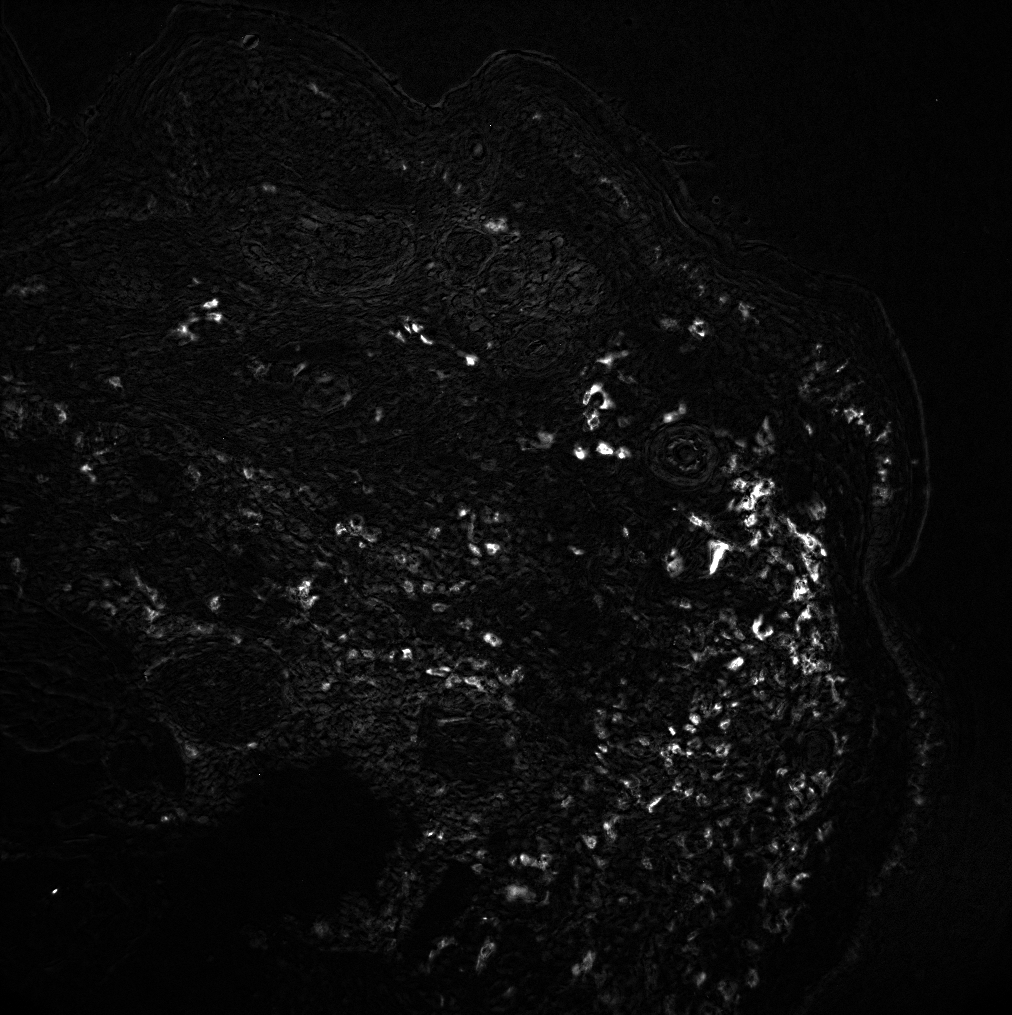

Supplement: Supplementary file 3 — Source Data for Expanded View and Appendix [file EMMM-15-e16796-s010.zip › Source Data/Supplement Figure 5/5A/24h/CD11c_CD206.tif]

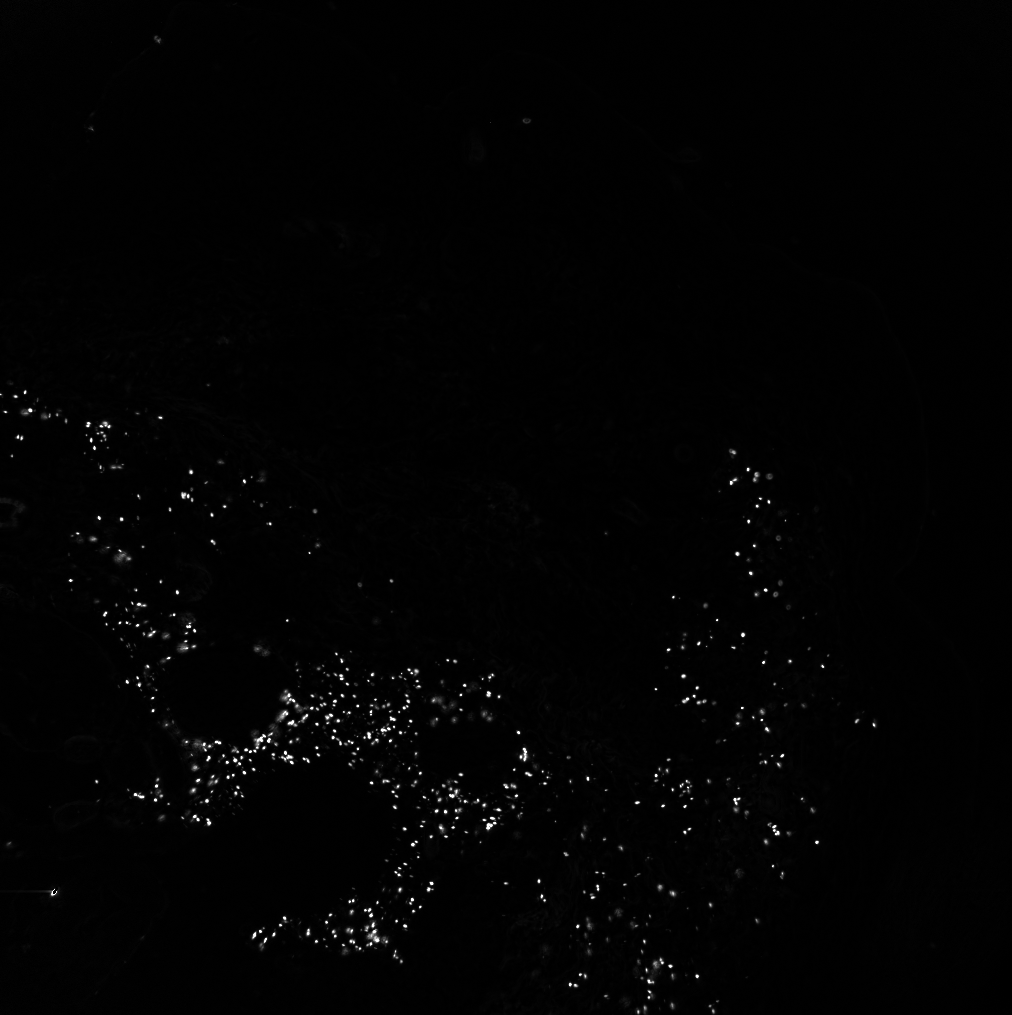

Supplement: Supplementary file 3 — Source Data for Expanded View and Appendix [file EMMM-15-e16796-s010.zip › Source Data/Supplement Figure 5/5A/24h/Zymosan.tif]

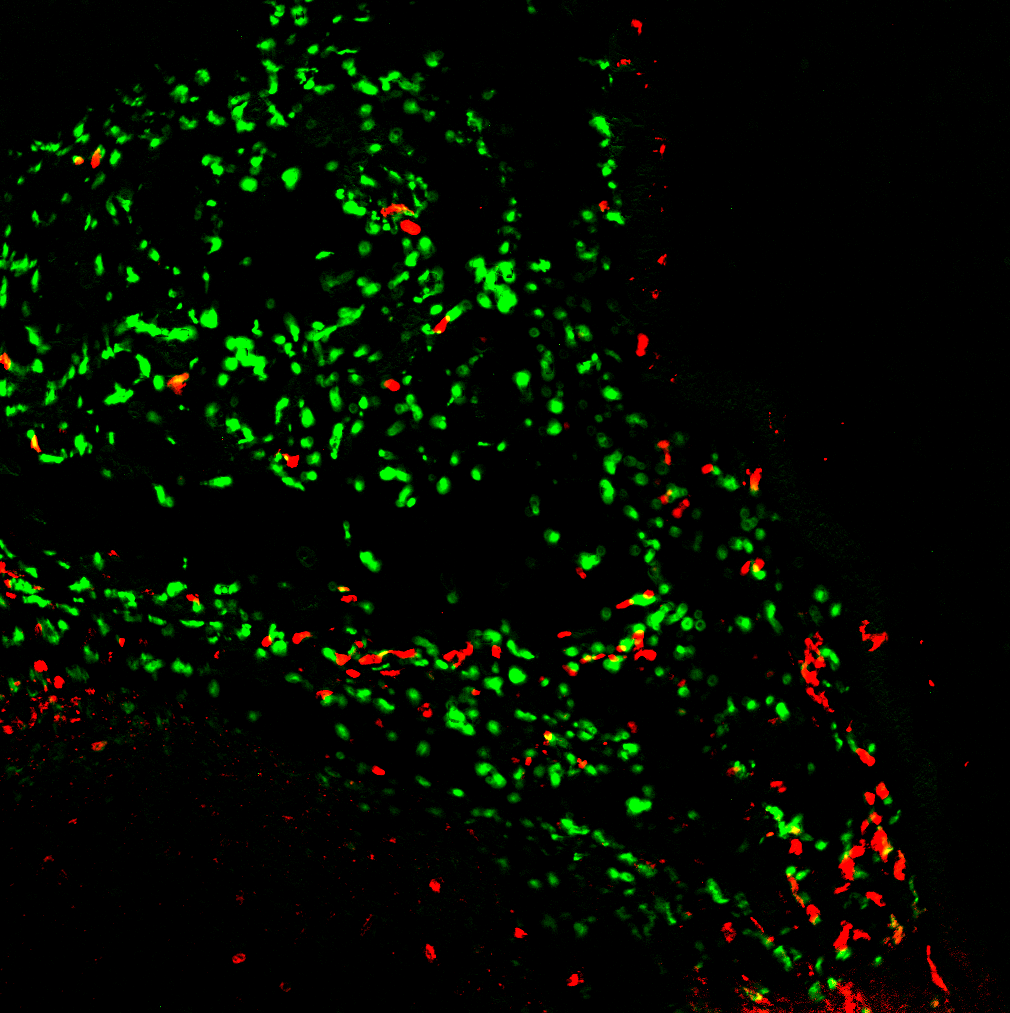

Supplement: Supplementary file 3 — Source Data for Expanded View and Appendix [file EMMM-15-e16796-s010.zip › Source Data/Supplement Figure 5/5A/48h/CD11c_CD206.tif]

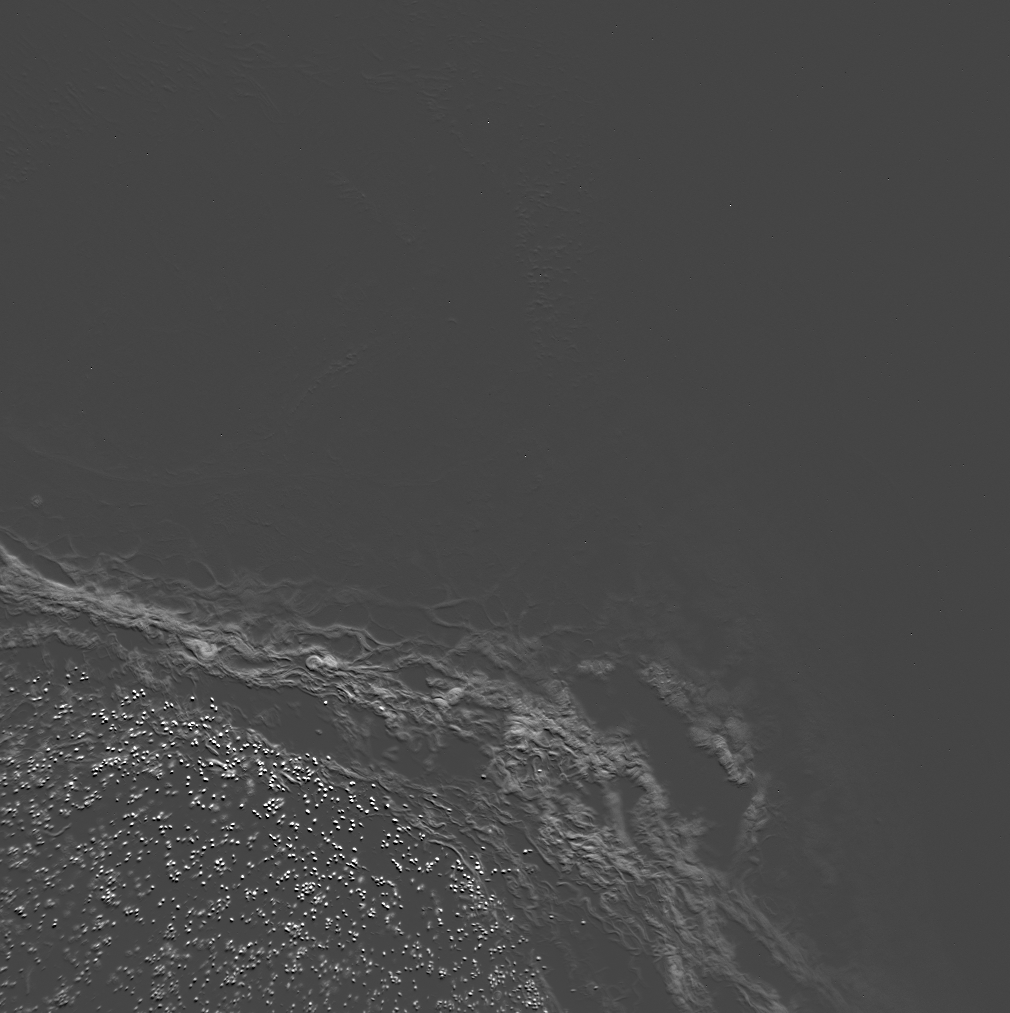

Supplement: Supplementary file 3 — Source Data for Expanded View and Appendix [file EMMM-15-e16796-s010.zip › Source Data/Supplement Figure 5/5A/48h/Zymosan.tif]

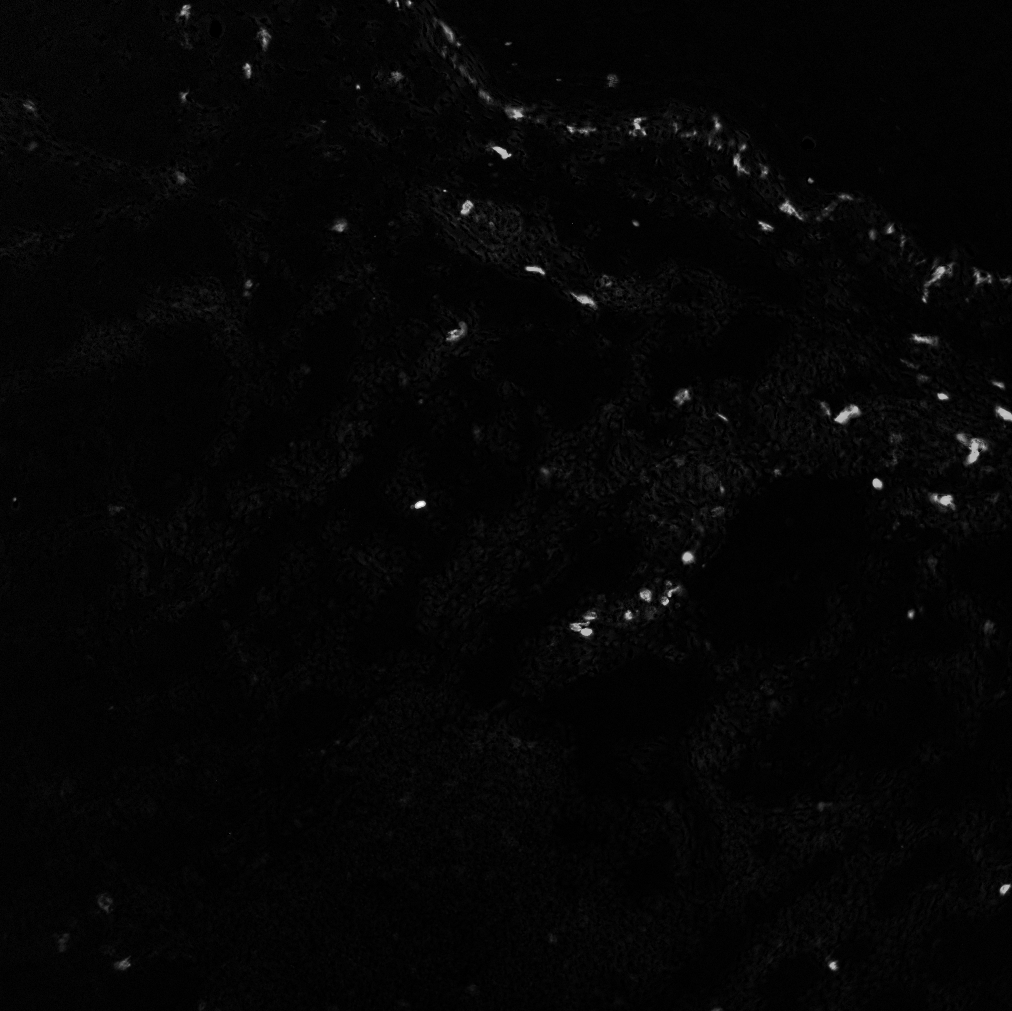

Supplement: Supplementary file 3 — Source Data for Expanded View and Appendix [file EMMM-15-e16796-s010.zip › Source Data/Supplement Figure 5/5A/4h/CD11c.tif]

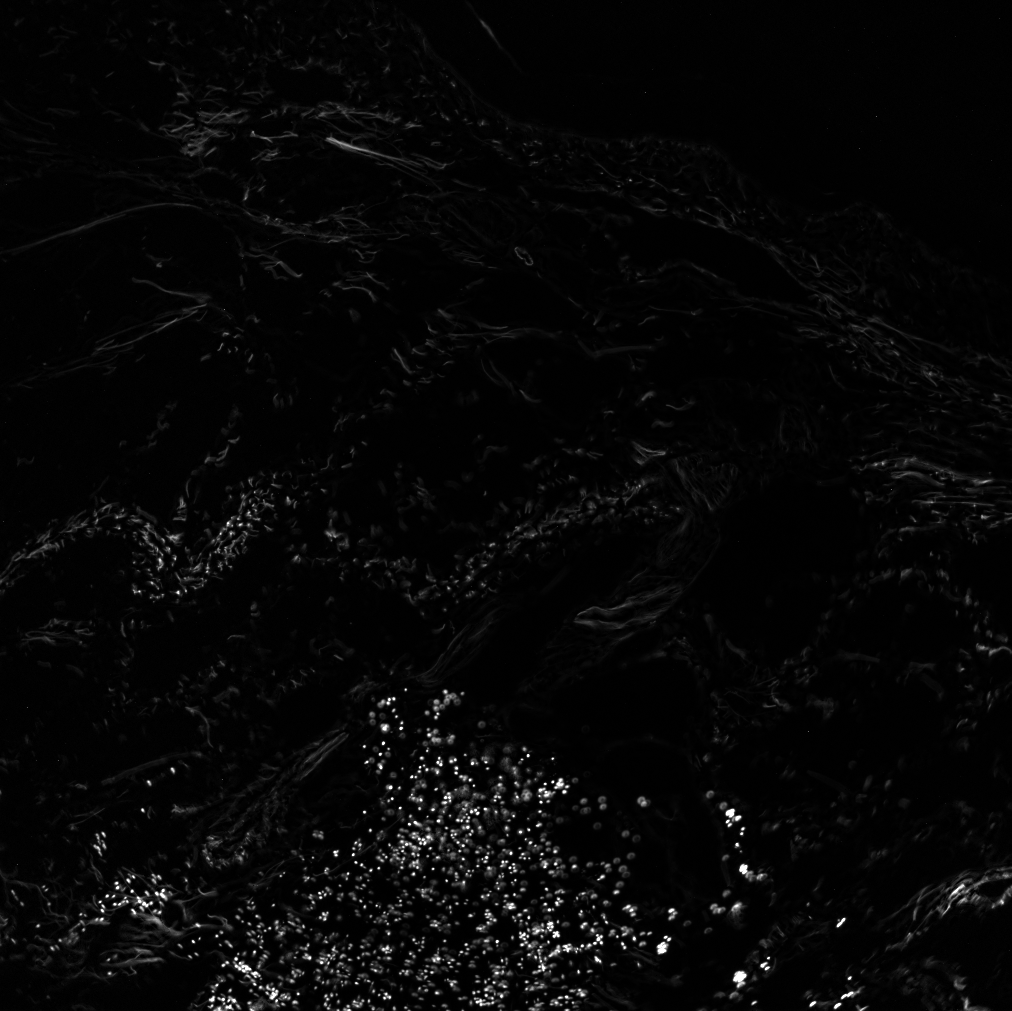

Supplement: Supplementary file 3 — Source Data for Expanded View and Appendix [file EMMM-15-e16796-s010.zip › Source Data/Supplement Figure 5/5A/4h/Zymosan.tif]

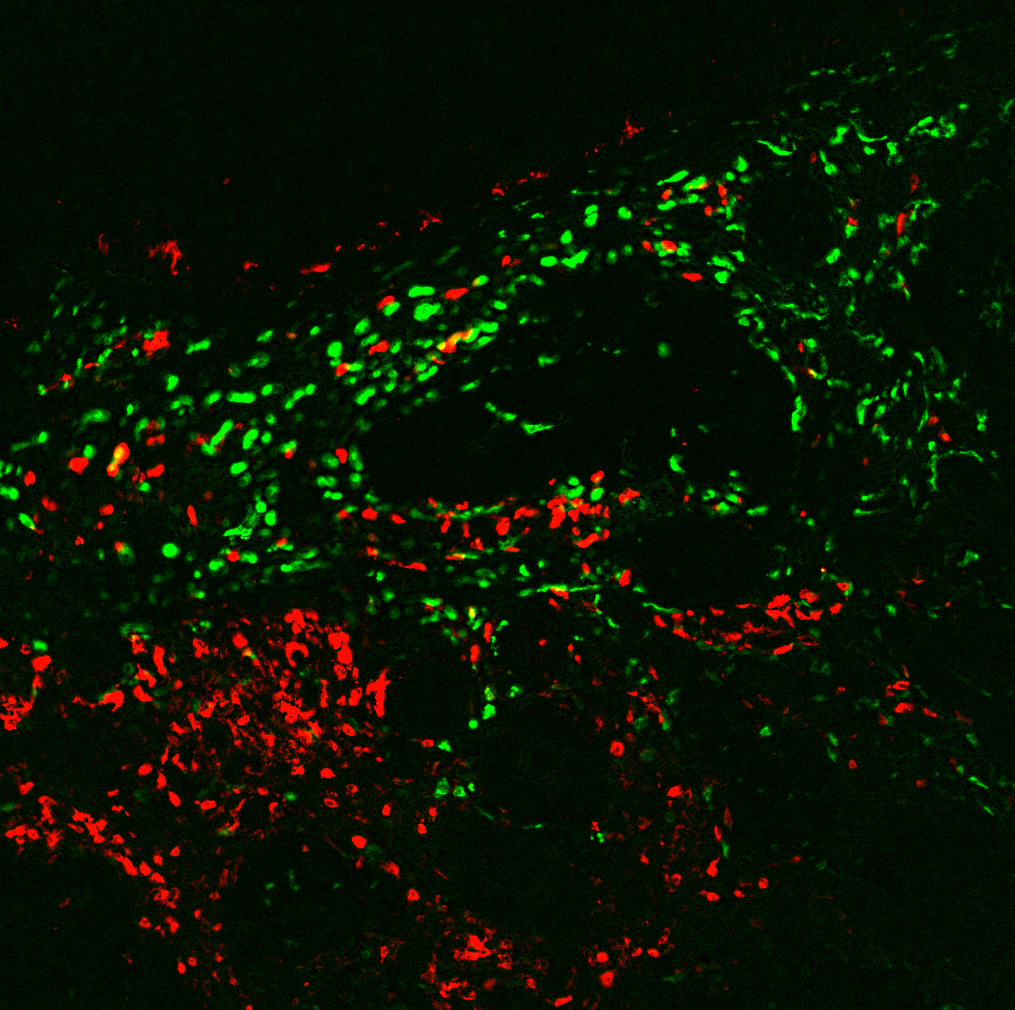

Supplement: Supplementary file 3 — Source Data for Expanded View and Appendix [file EMMM-15-e16796-s010.zip › Source Data/Supplement Figure 5/5A/72h/CD11c_CD206.tif]

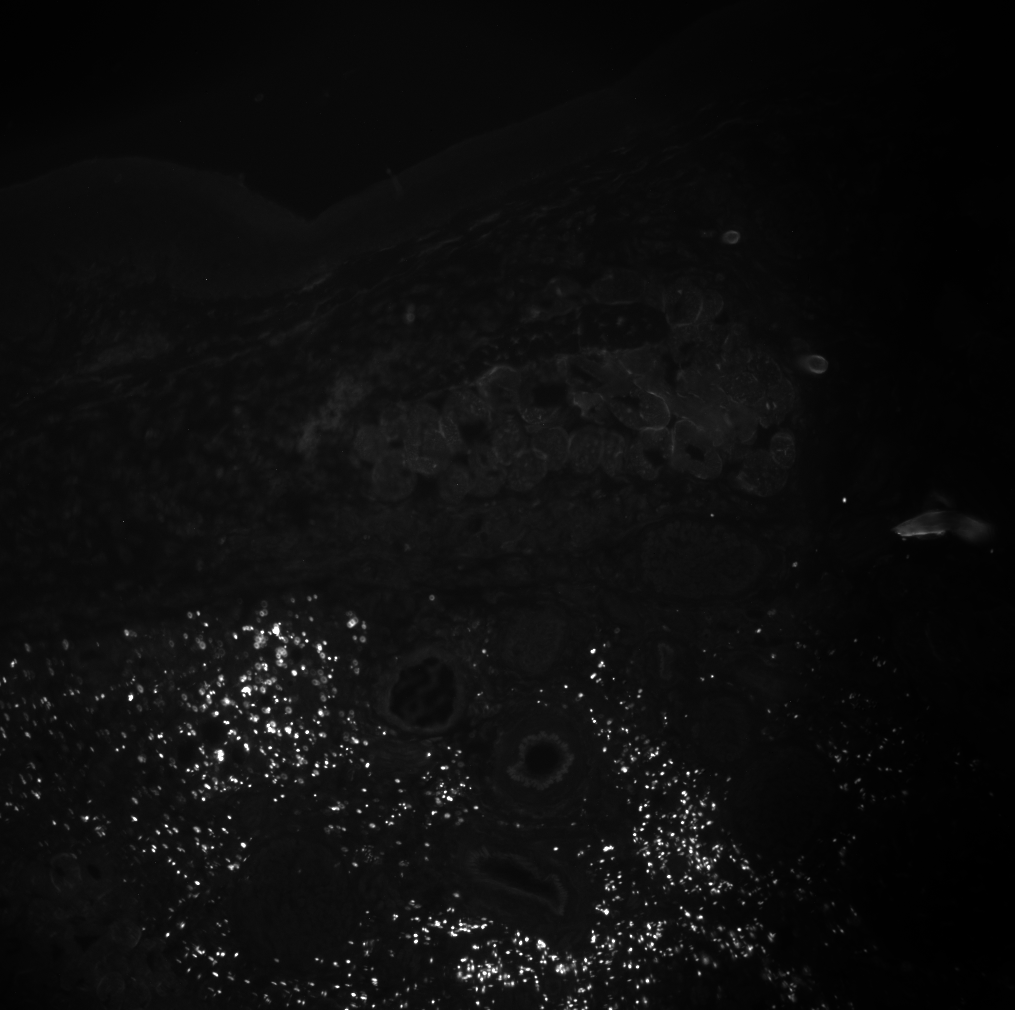

Supplement: Supplementary file 3 — Source Data for Expanded View and Appendix [file EMMM-15-e16796-s010.zip › Source Data/Supplement Figure 5/5A/72h/Zymosan.tif]

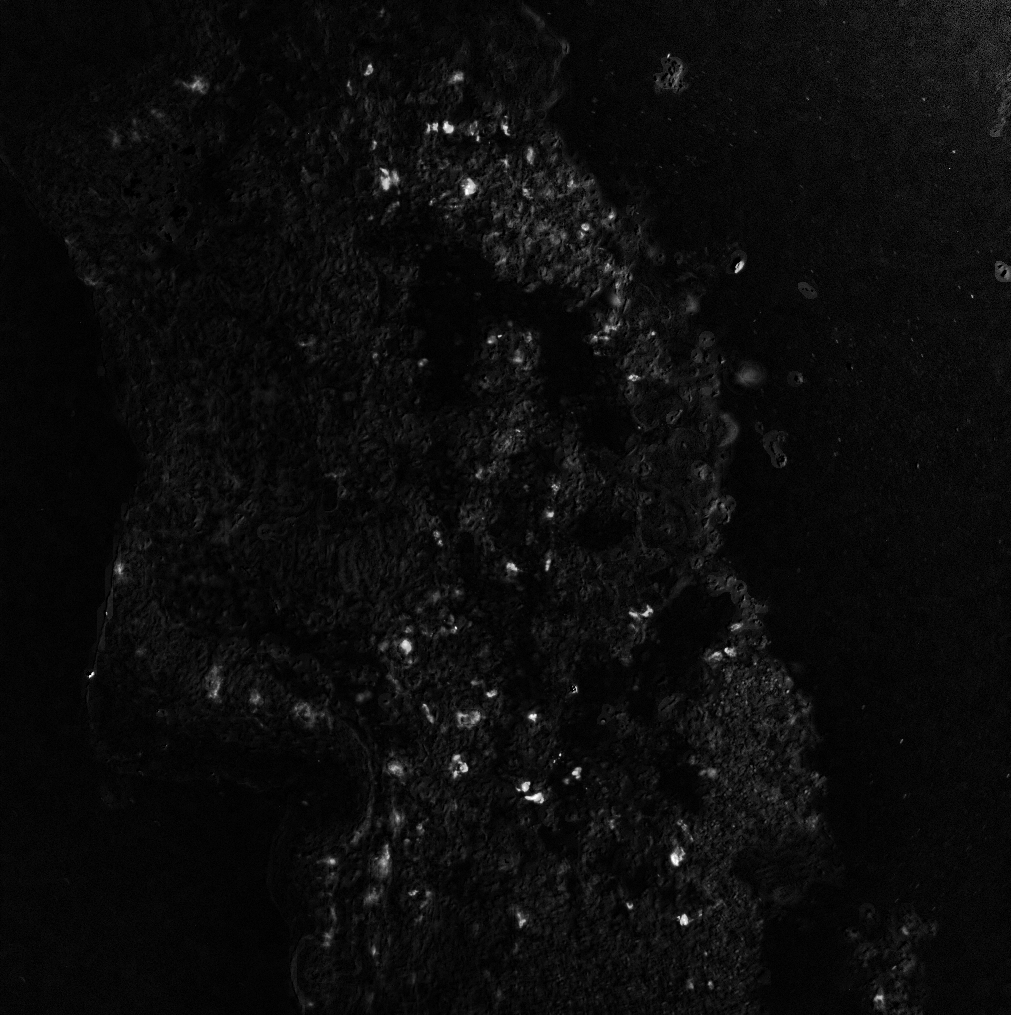

Supplement: Supplementary file 3 — Source Data for Expanded View and Appendix [file EMMM-15-e16796-s010.zip › Source Data/Supplement Figure 5/5A/8h/CD11c.tif]

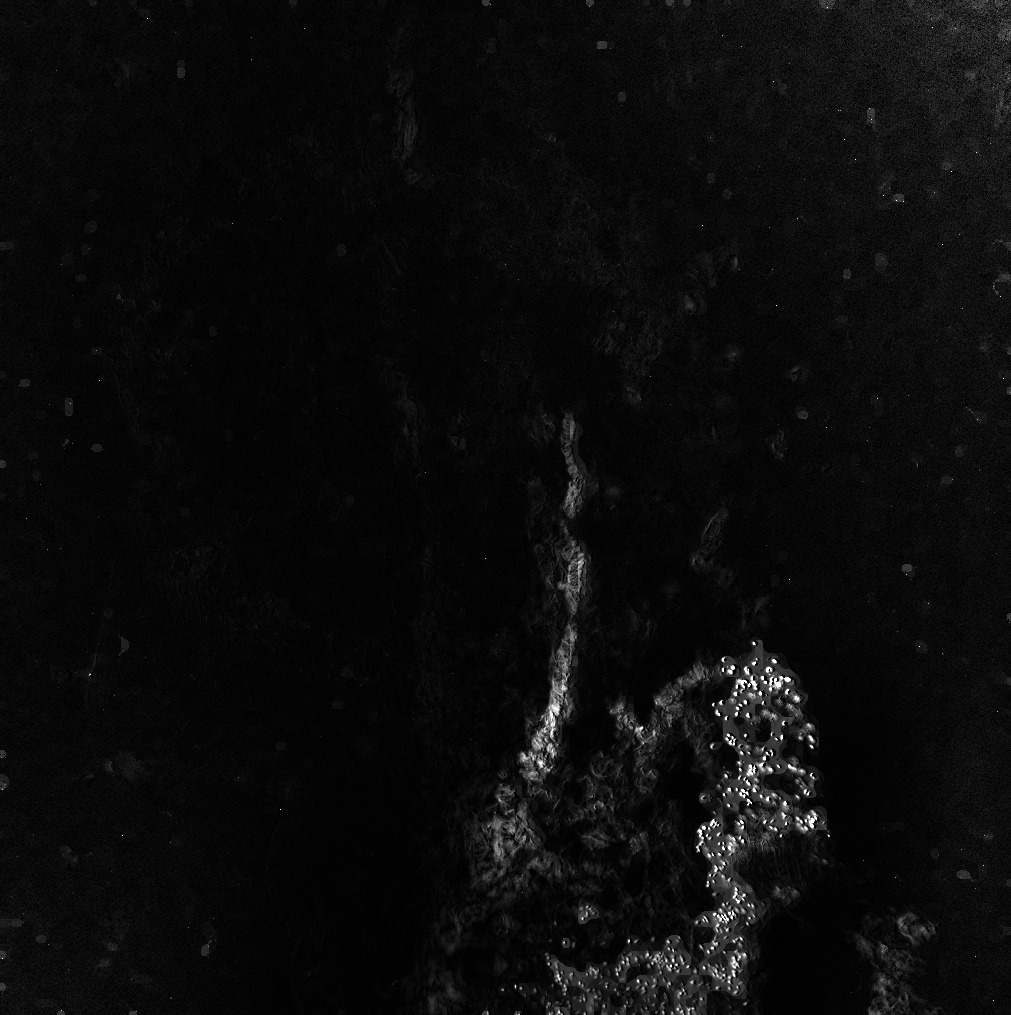

Supplement: Supplementary file 3 — Source Data for Expanded View and Appendix [file EMMM-15-e16796-s010.zip › Source Data/Supplement Figure 5/5A/8h/Zymosan.tif]

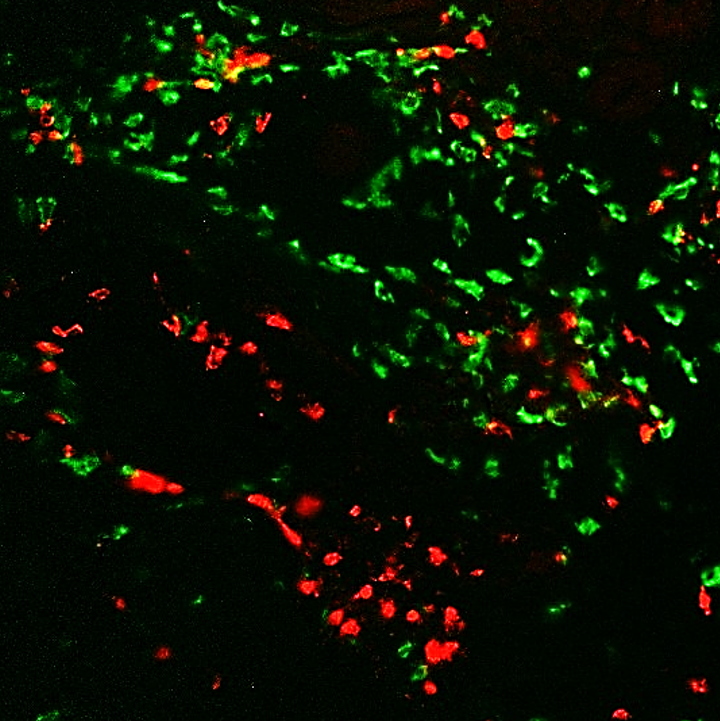

Supplement: Supplementary file 3 — Source Data for Expanded View and Appendix [file EMMM-15-e16796-s010.zip › Source Data/Supplement Figure 5/5B/24h/Siglec F_CD206.tif]

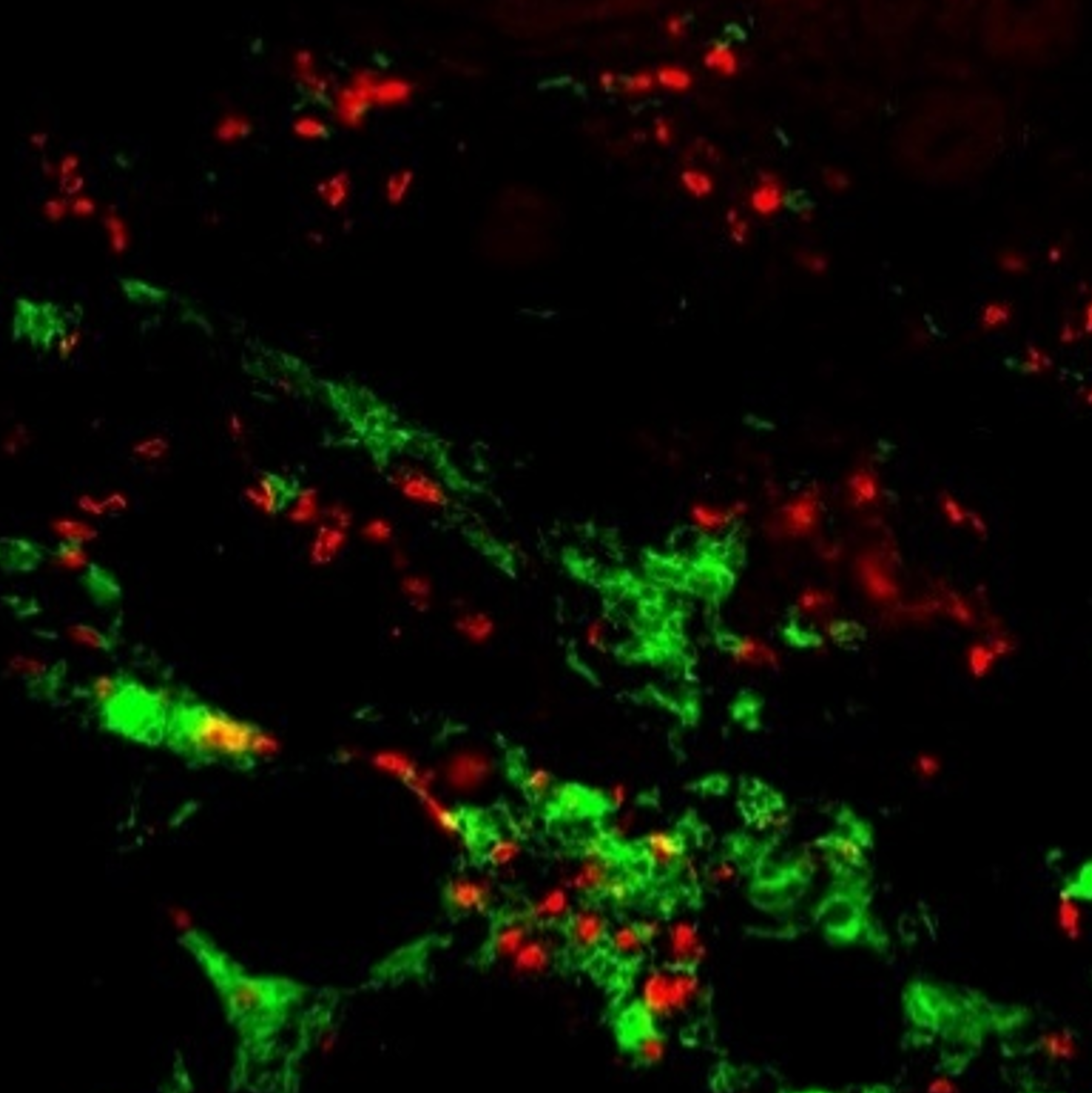

Supplement: Supplementary file 3 — Source Data for Expanded View and Appendix [file EMMM-15-e16796-s010.zip › Source Data/Supplement Figure 5/5B/24h/SiglecF_CD86.tif]

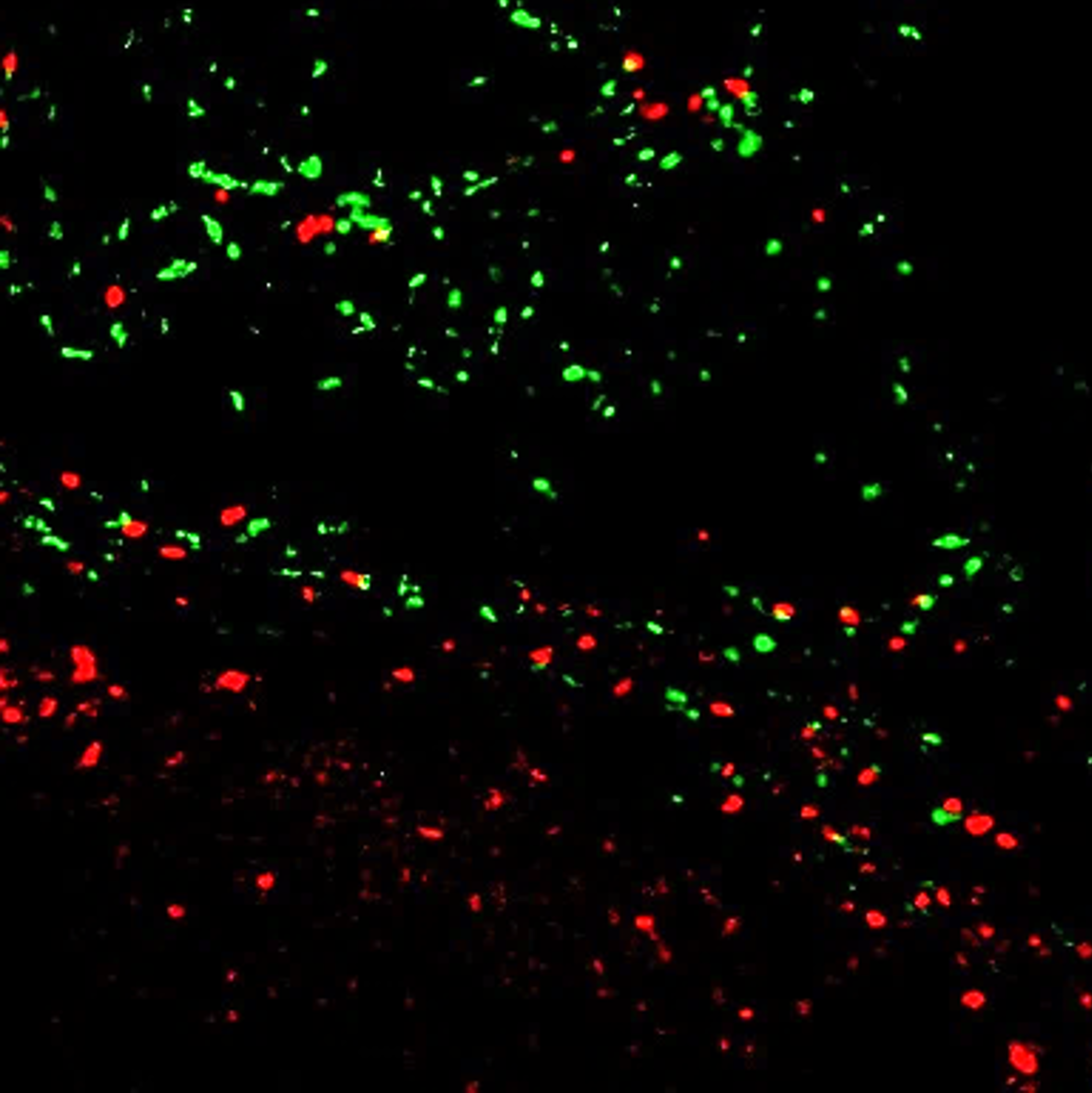

Supplement: Supplementary file 3 — Source Data for Expanded View and Appendix [file EMMM-15-e16796-s010.zip › Source Data/Supplement Figure 5/5B/48h/Siglec F_CD206.tif]

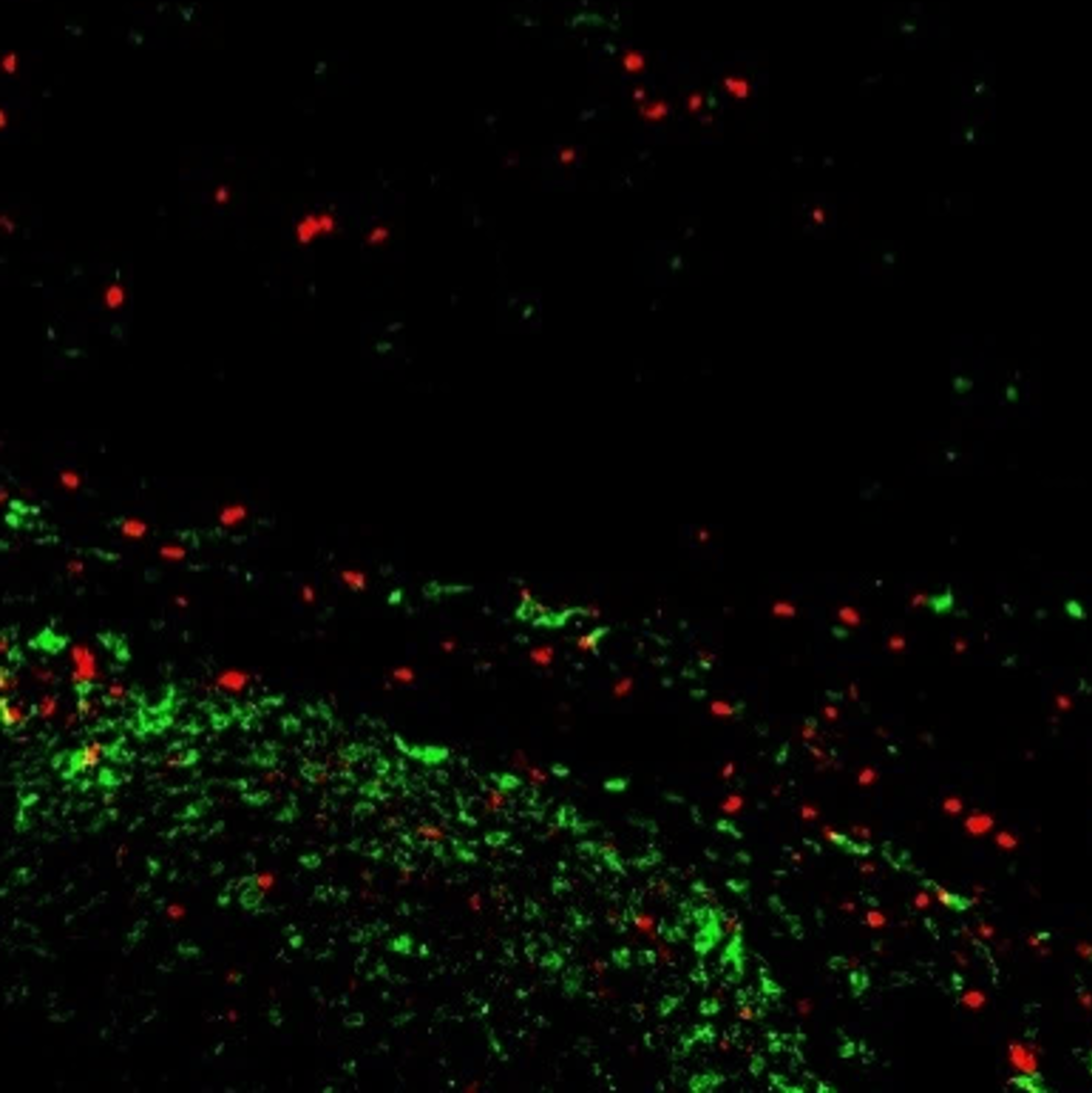

Supplement: Supplementary file 3 — Source Data for Expanded View and Appendix [file EMMM-15-e16796-s010.zip › Source Data/Supplement Figure 5/5B/48h/Siglec F_CD86.tif]

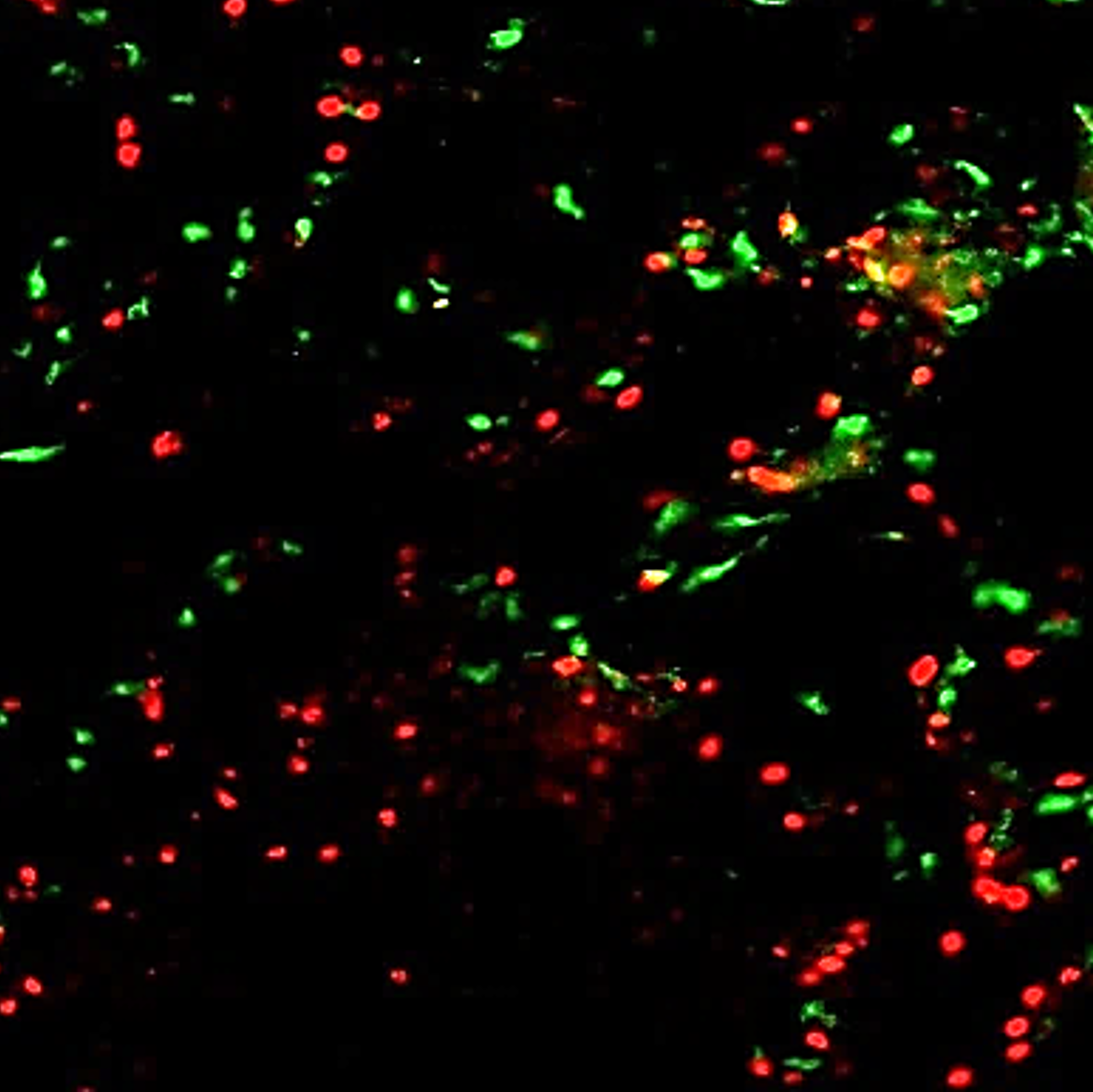

Supplement: Supplementary file 3 — Source Data for Expanded View and Appendix [file EMMM-15-e16796-s010.zip › Source Data/Supplement Figure 5/5B/4h/SiglecF_CD206.tif]

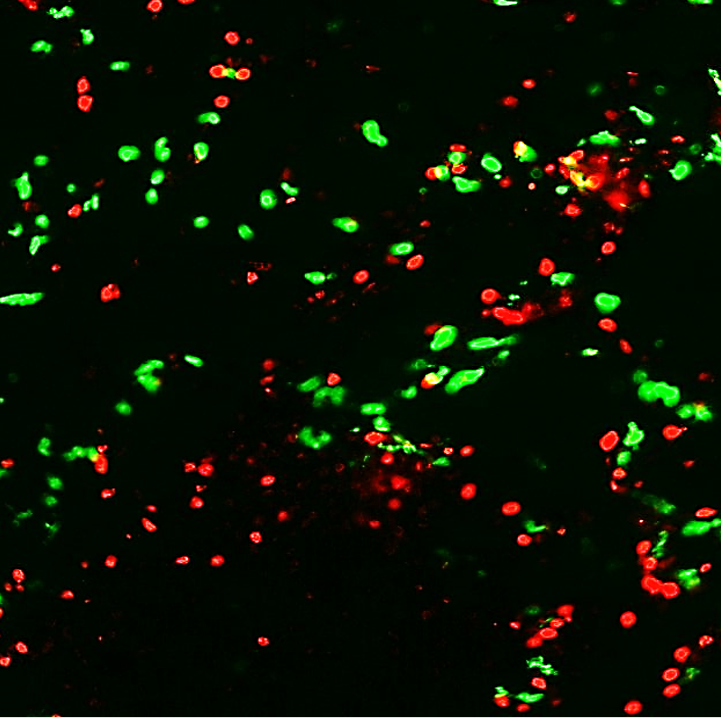

Supplement: Supplementary file 3 — Source Data for Expanded View and Appendix [file EMMM-15-e16796-s010.zip › Source Data/Supplement Figure 5/5B/4h/SiglecF_CD86.tif]

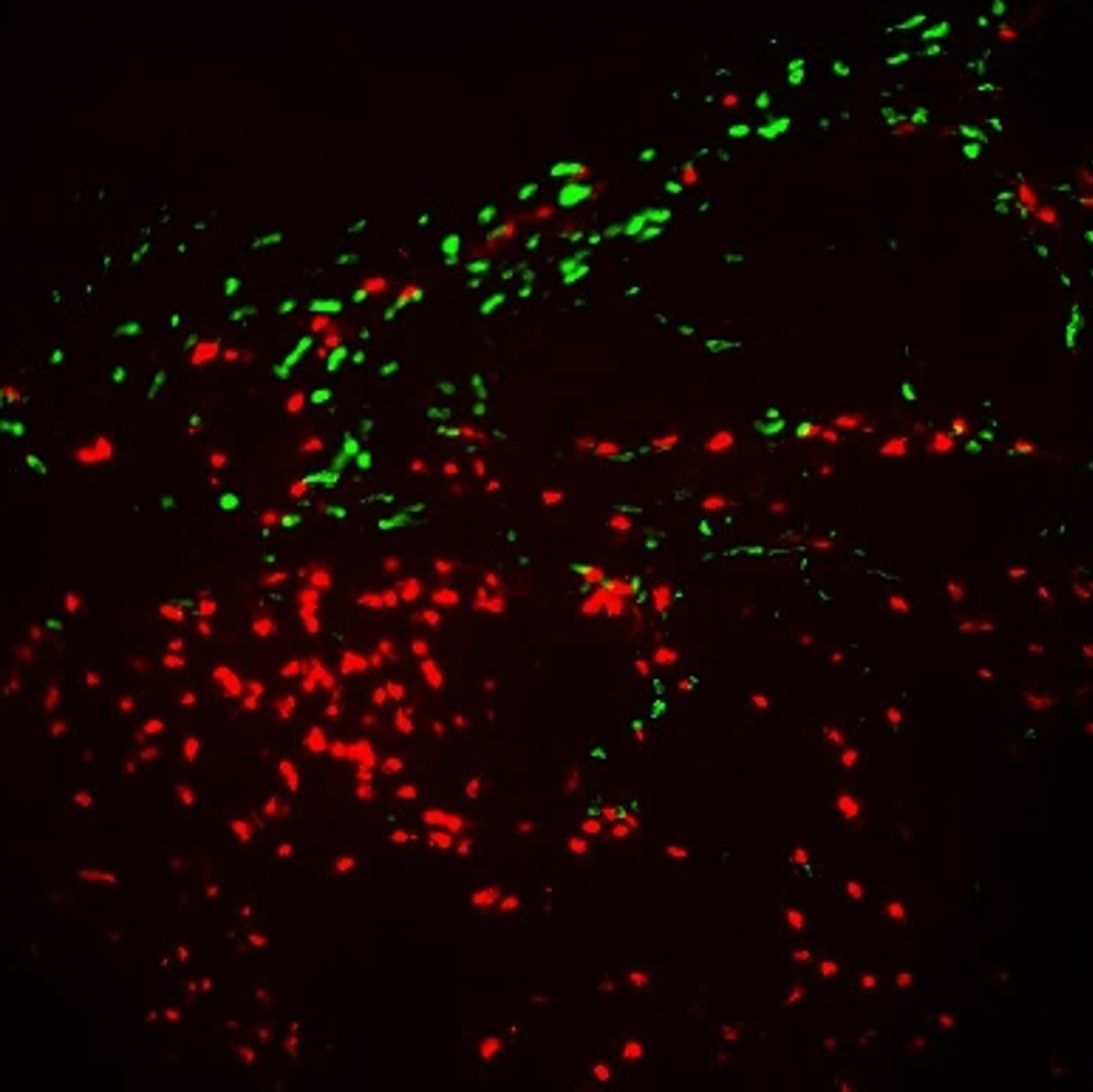

Supplement: Supplementary file 3 — Source Data for Expanded View and Appendix [file EMMM-15-e16796-s010.zip › Source Data/Supplement Figure 5/5B/72h/Siglec F_CD206.tif]

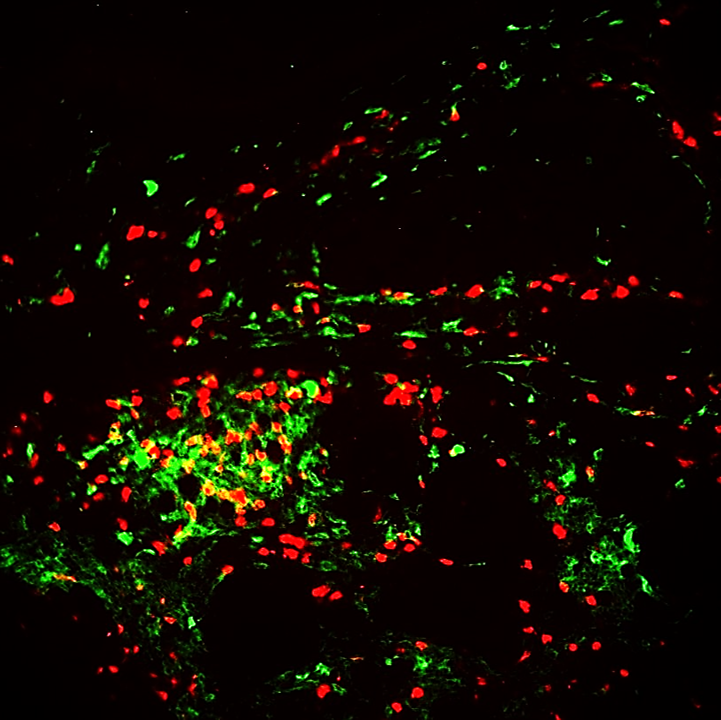

Supplement: Supplementary file 3 — Source Data for Expanded View and Appendix [file EMMM-15-e16796-s010.zip › Source Data/Supplement Figure 5/5B/72h/Siglec F_CD86.tif]

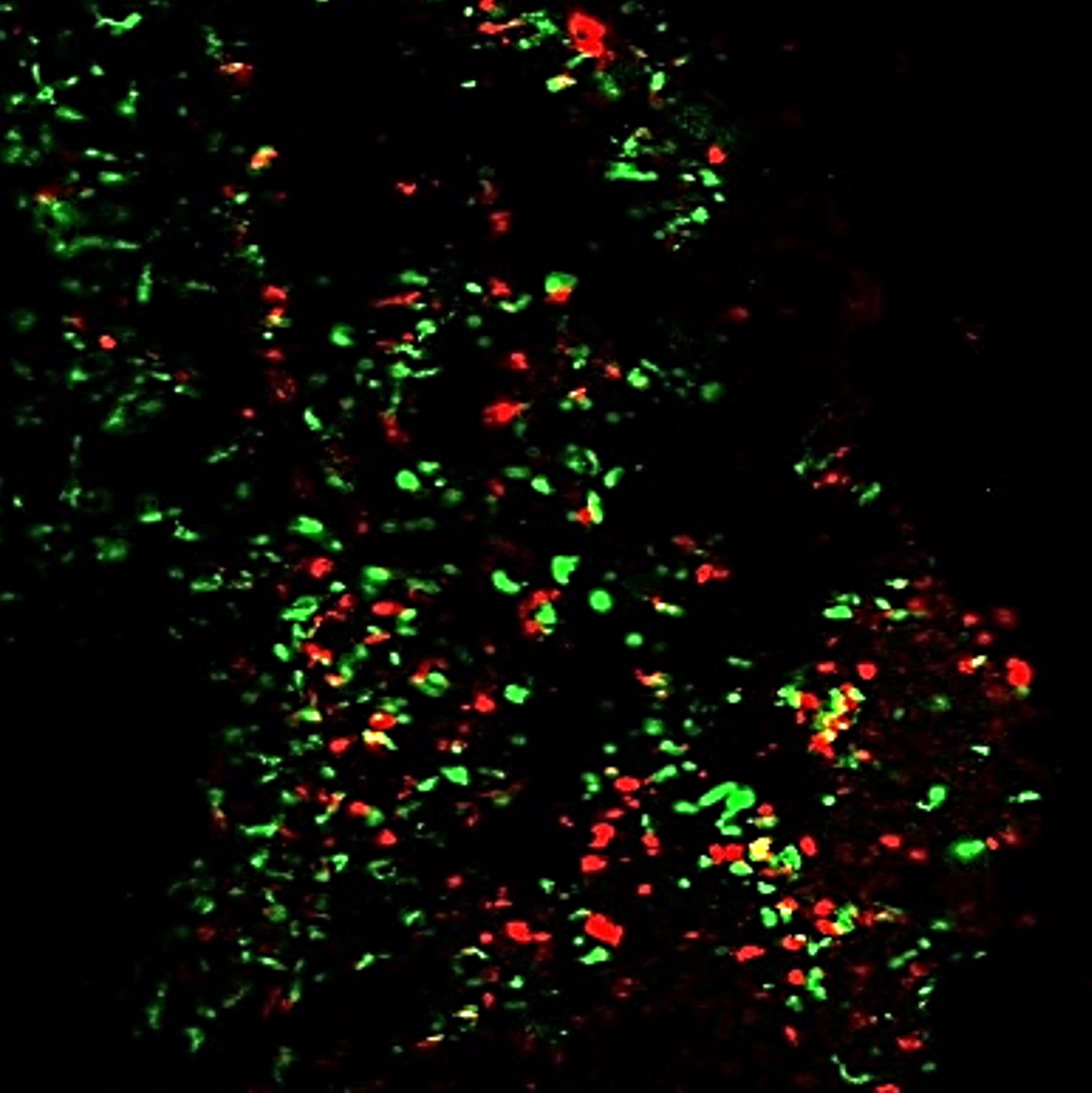

Supplement: Supplementary file 3 — Source Data for Expanded View and Appendix [file EMMM-15-e16796-s010.zip › Source Data/Supplement Figure 5/5B/8h/SiglecF_CD206.tif]

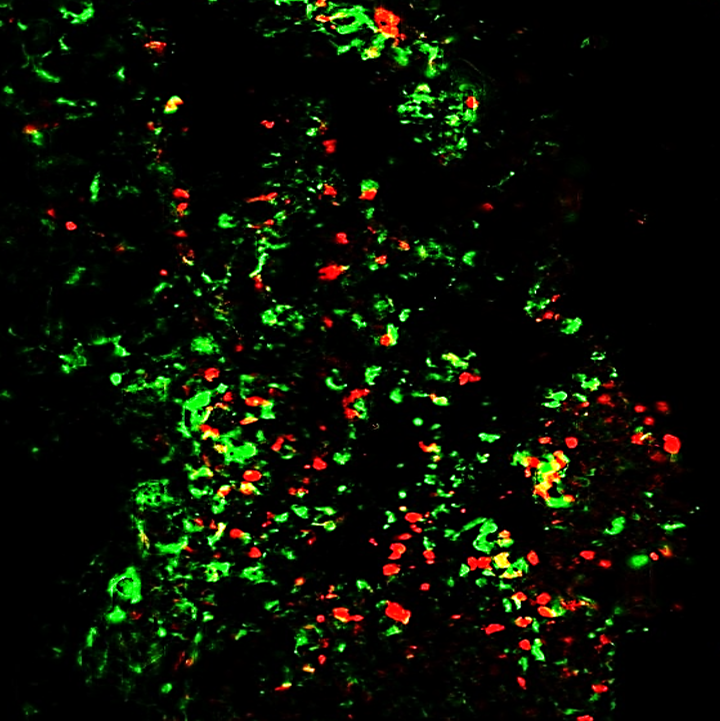

Supplement: Supplementary file 3 — Source Data for Expanded View and Appendix [file EMMM-15-e16796-s010.zip › Source Data/Supplement Figure 5/5B/8h/SiglecF_CD86.tif]

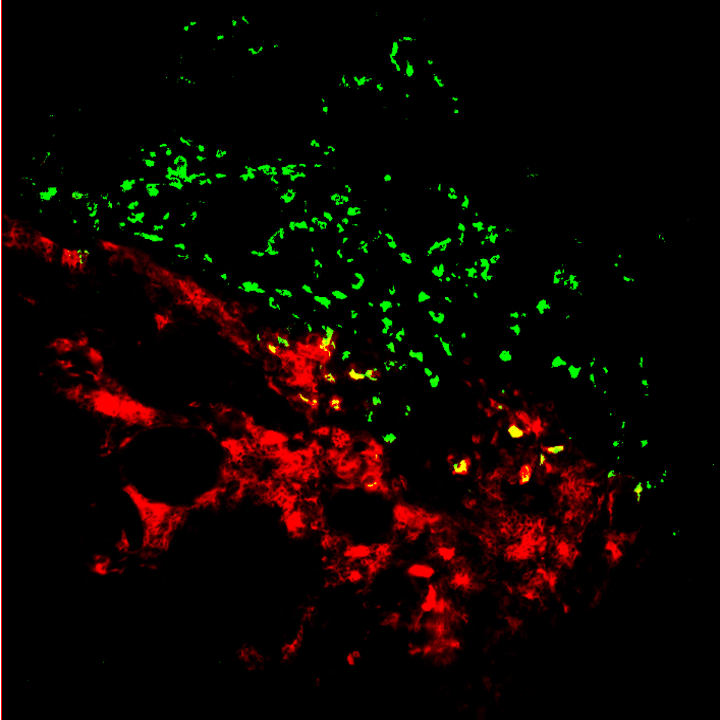

Supplement: Supplementary file 5 — Source Data for Figure 1 [file EMMM-15-e16796-s009.zip › EMMM202216796-sup-0004-SDataFig1/1A/24h/CD206_CD86.tif]

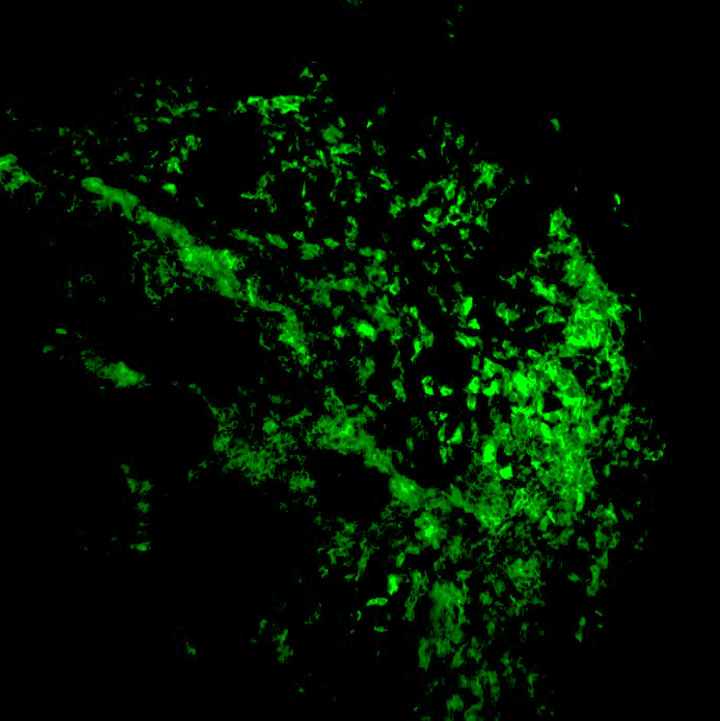

Supplement: Supplementary file 5 — Source Data for Figure 1 [file EMMM-15-e16796-s009.zip › EMMM202216796-sup-0004-SDataFig1/1A/24h/F4-80.tif]

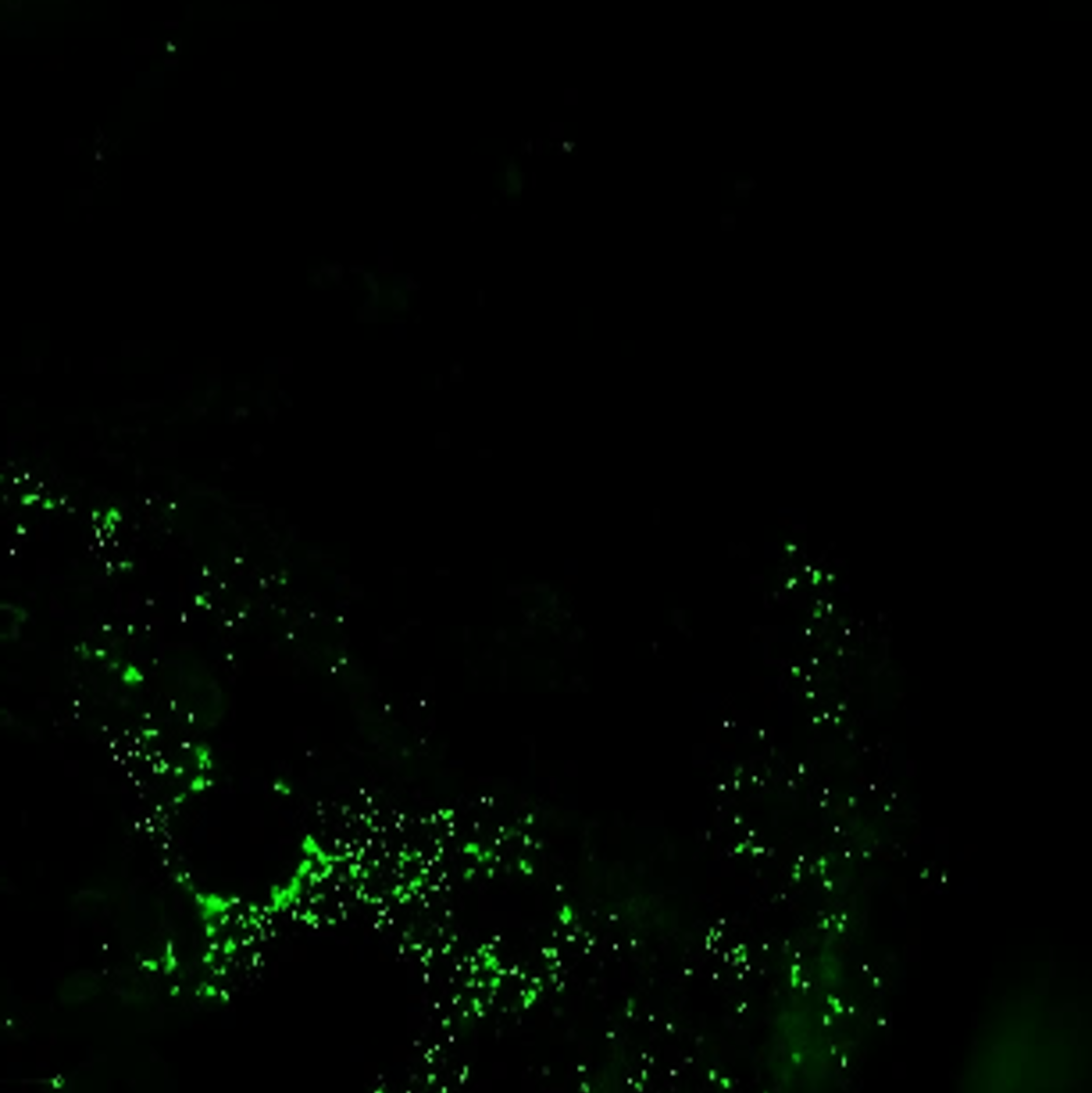

Supplement: Supplementary file 5 — Source Data for Figure 1 [file EMMM-15-e16796-s009.zip › EMMM202216796-sup-0004-SDataFig1/1A/24h/Zymosan.tif]

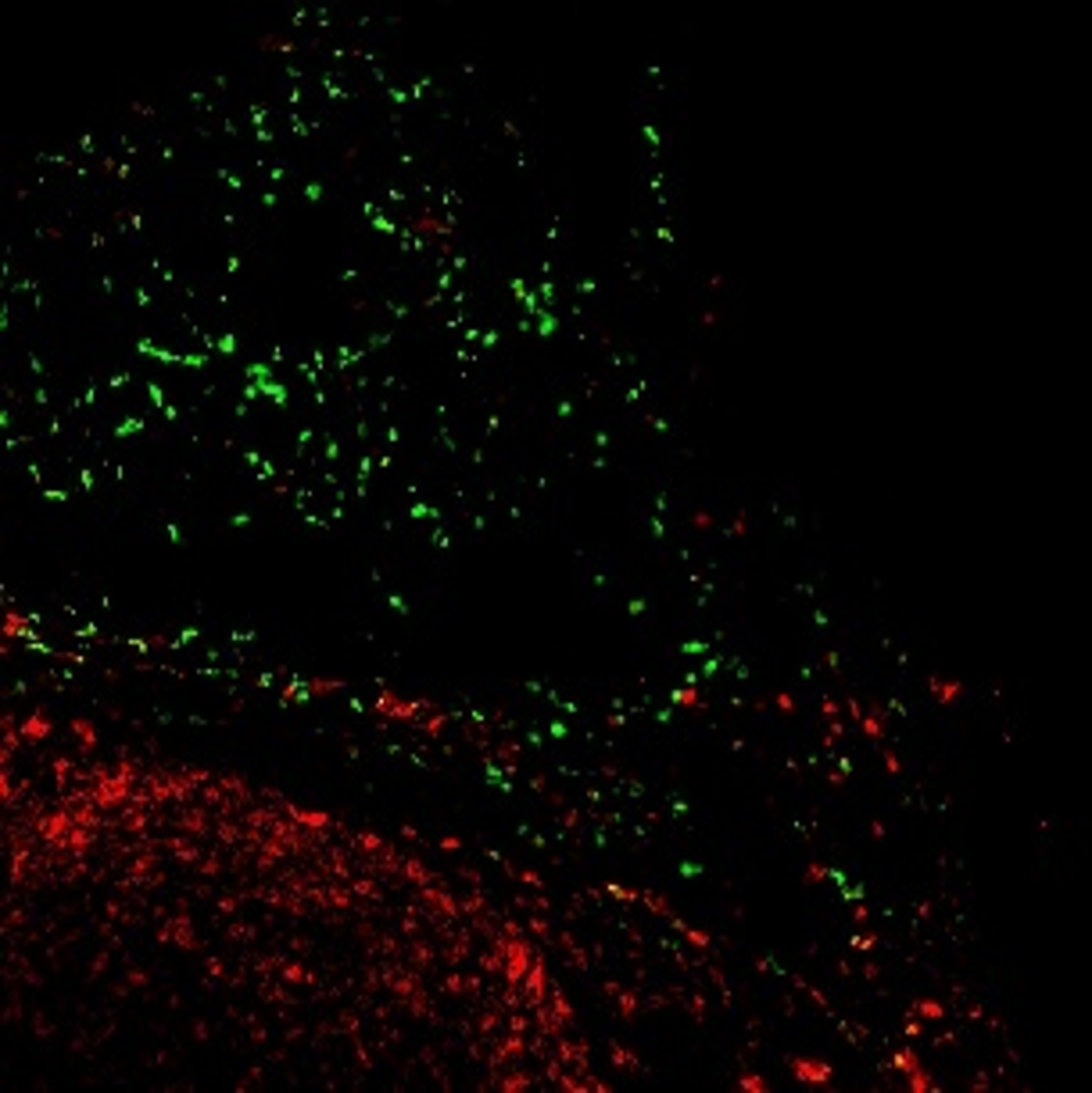

Supplement: Supplementary file 5 — Source Data for Figure 1 [file EMMM-15-e16796-s009.zip › EMMM202216796-sup-0004-SDataFig1/1A/48h/CD206_CD86.tif]

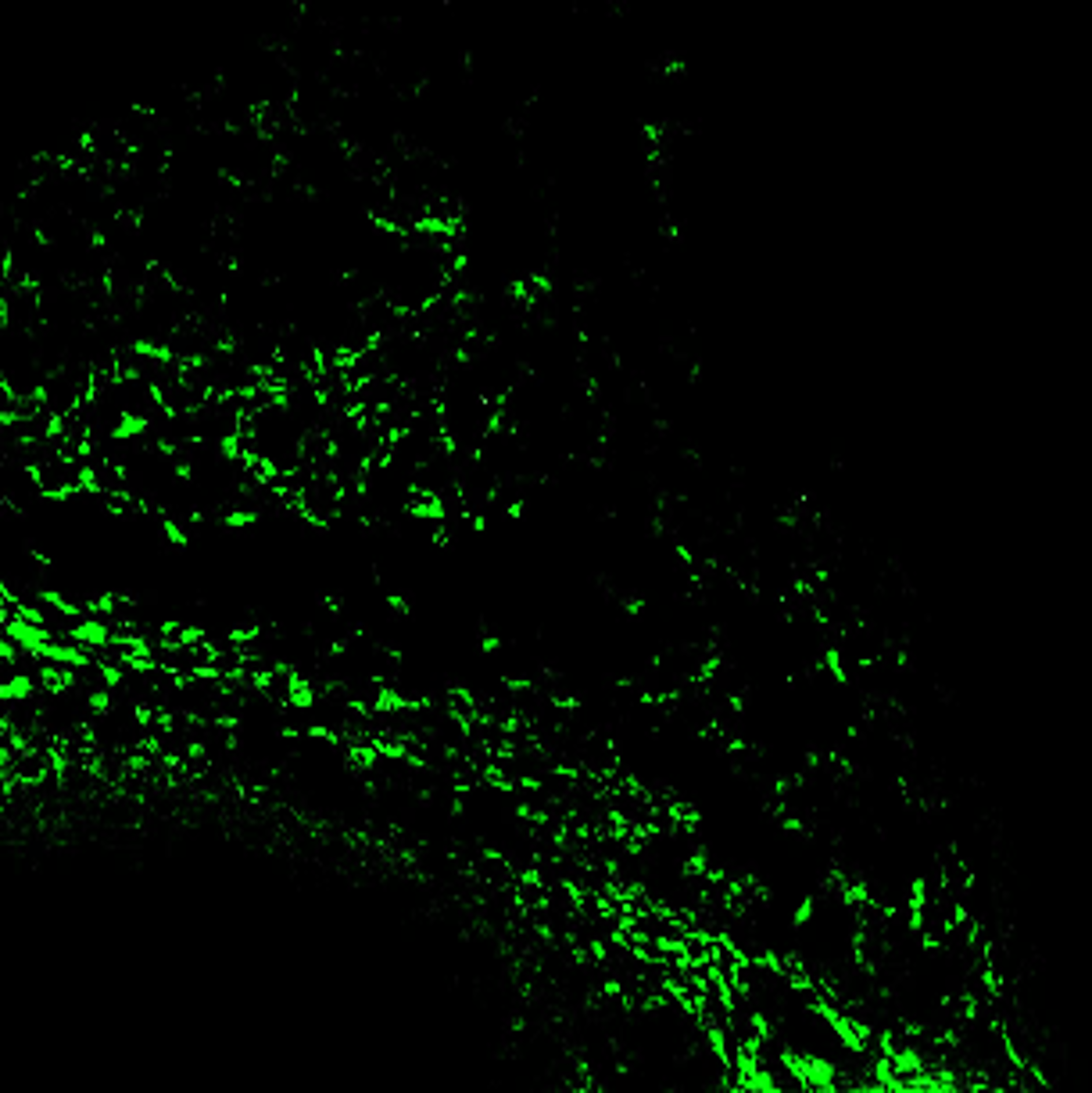

Supplement: Supplementary file 5 — Source Data for Figure 1 [file EMMM-15-e16796-s009.zip › EMMM202216796-sup-0004-SDataFig1/1A/48h/F4-80.tif]

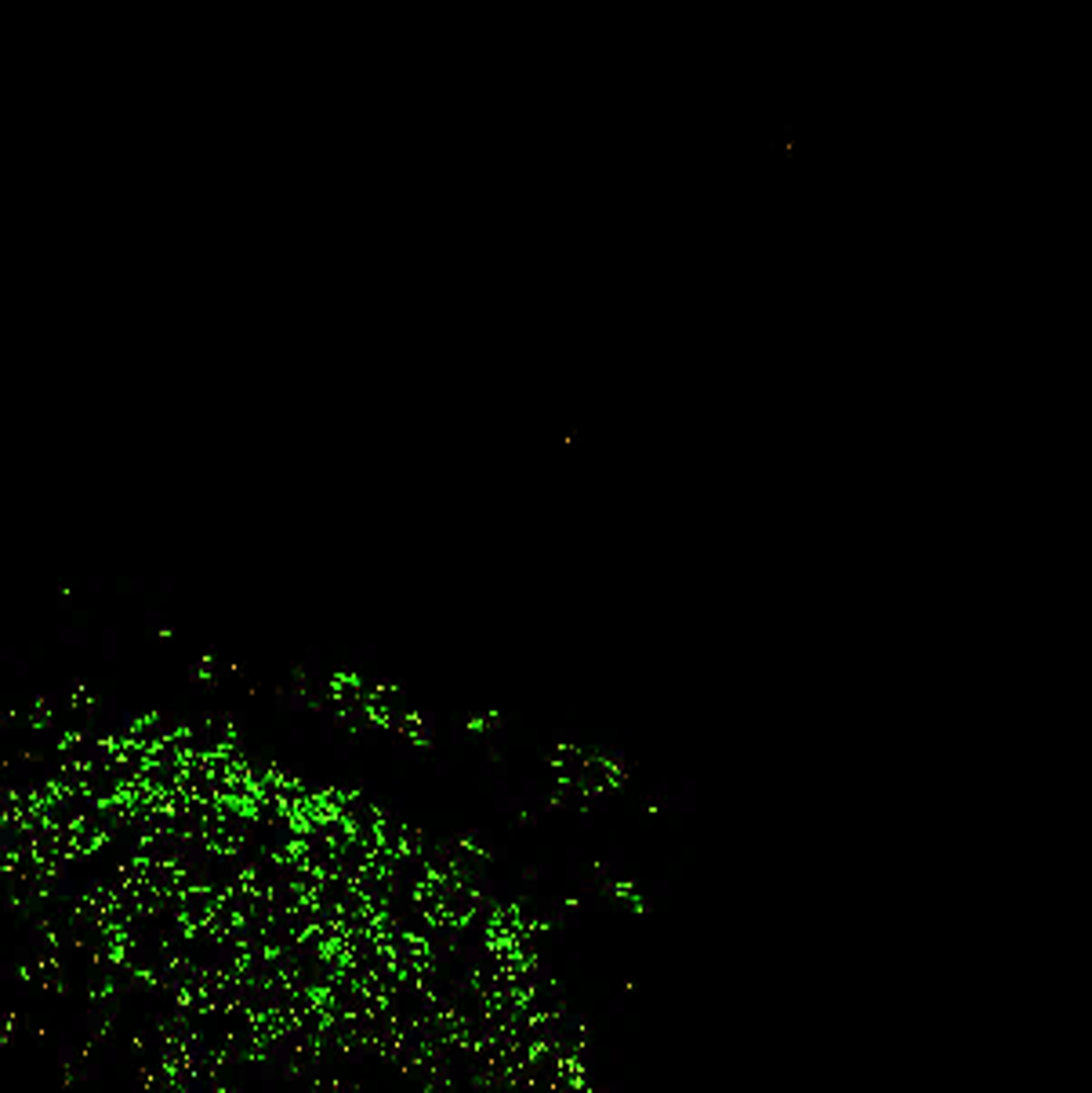

Supplement: Supplementary file 5 — Source Data for Figure 1 [file EMMM-15-e16796-s009.zip › EMMM202216796-sup-0004-SDataFig1/1A/48h/Zymosan.tif]

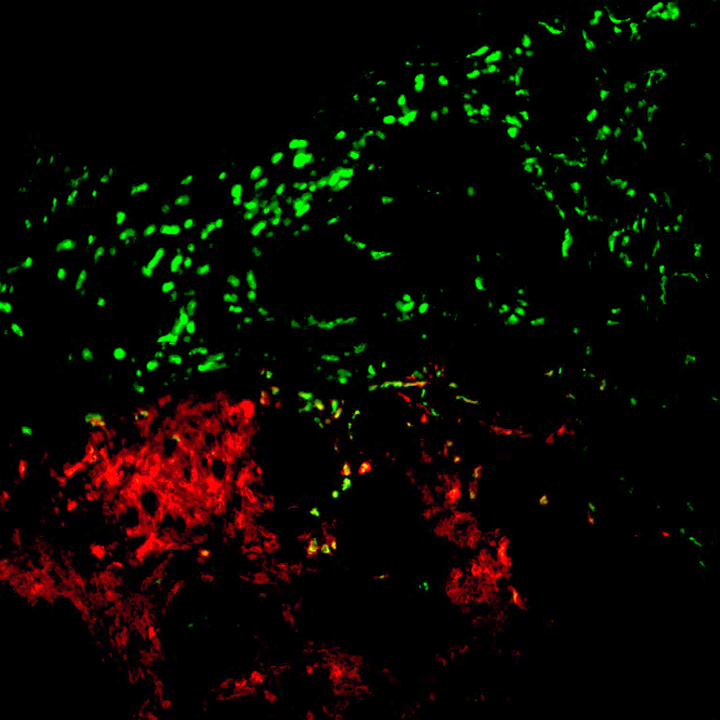

Supplement: Supplementary file 5 — Source Data for Figure 1 [file EMMM-15-e16796-s009.zip › EMMM202216796-sup-0004-SDataFig1/1A/72h/CD206_CD86.tif]

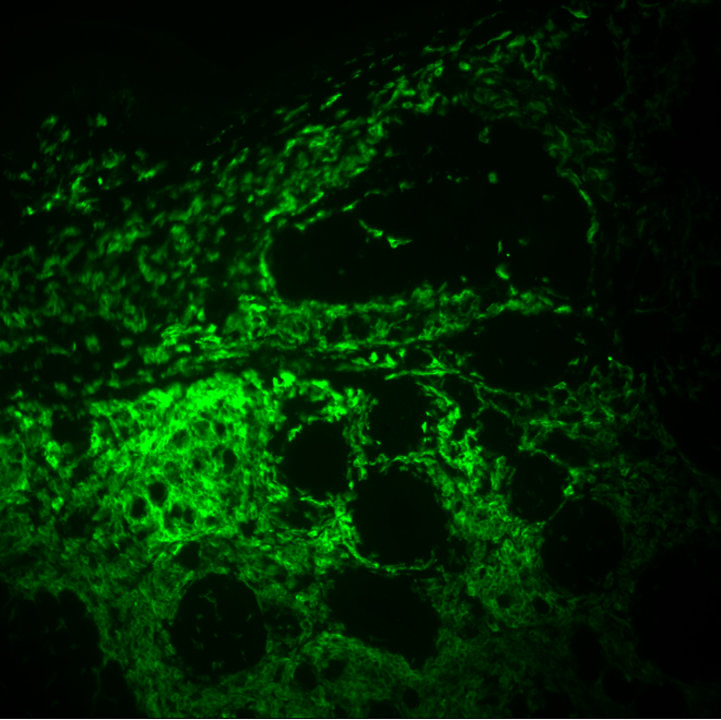

Supplement: Supplementary file 5 — Source Data for Figure 1 [file EMMM-15-e16796-s009.zip › EMMM202216796-sup-0004-SDataFig1/1A/72h/F4-80.tif]

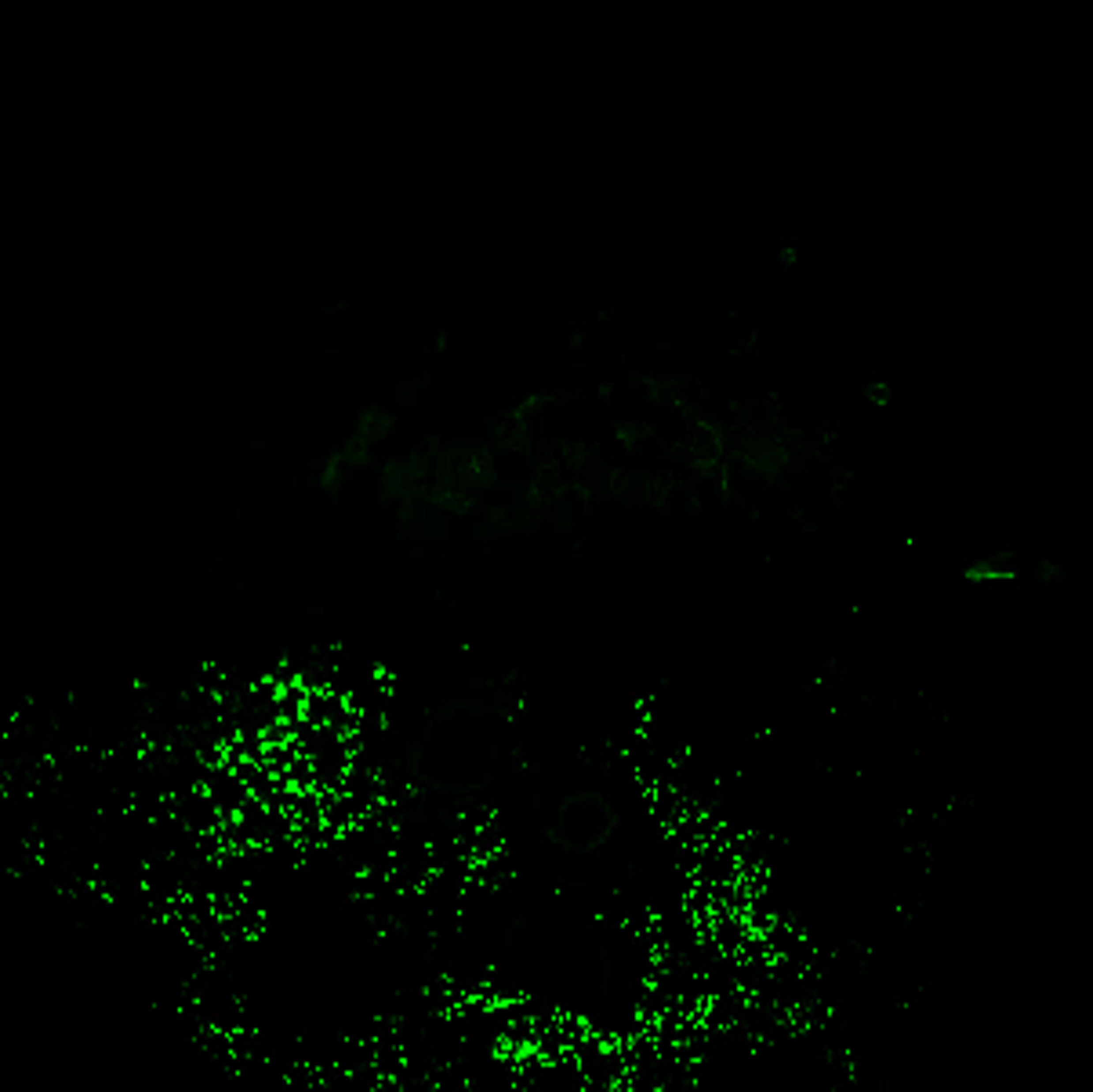

Supplement: Supplementary file 5 — Source Data for Figure 1 [file EMMM-15-e16796-s009.zip › EMMM202216796-sup-0004-SDataFig1/1A/72h/Zymosan.tif]

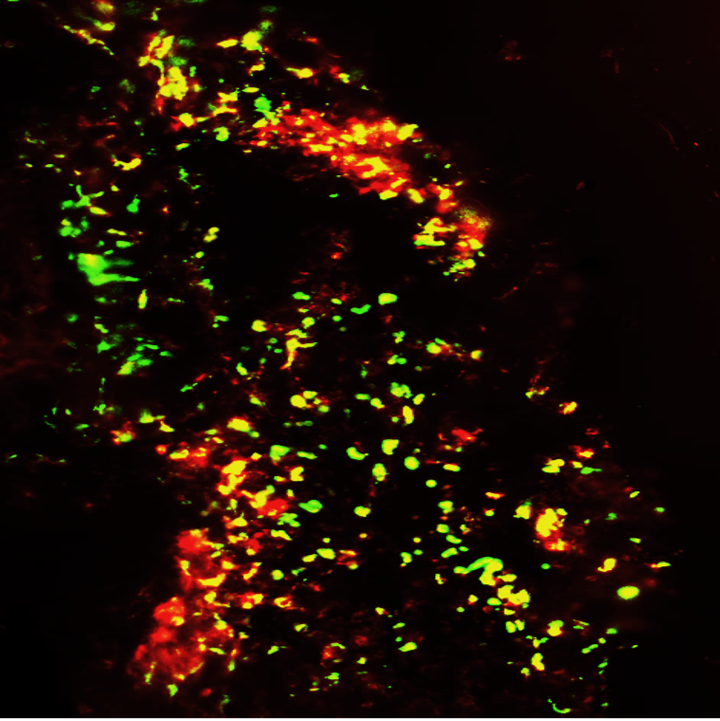

Supplement: Supplementary file 5 — Source Data for Figure 1 [file EMMM-15-e16796-s009.zip › EMMM202216796-sup-0004-SDataFig1/1A/8h/CD206_CD86.tif]

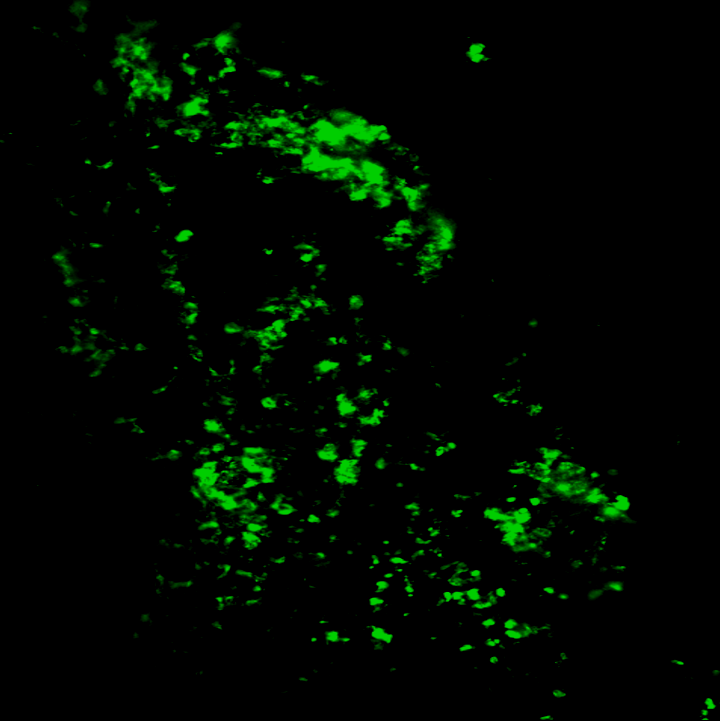

Supplement: Supplementary file 5 — Source Data for Figure 1 [file EMMM-15-e16796-s009.zip › EMMM202216796-sup-0004-SDataFig1/1A/8h/F4-80.tif]

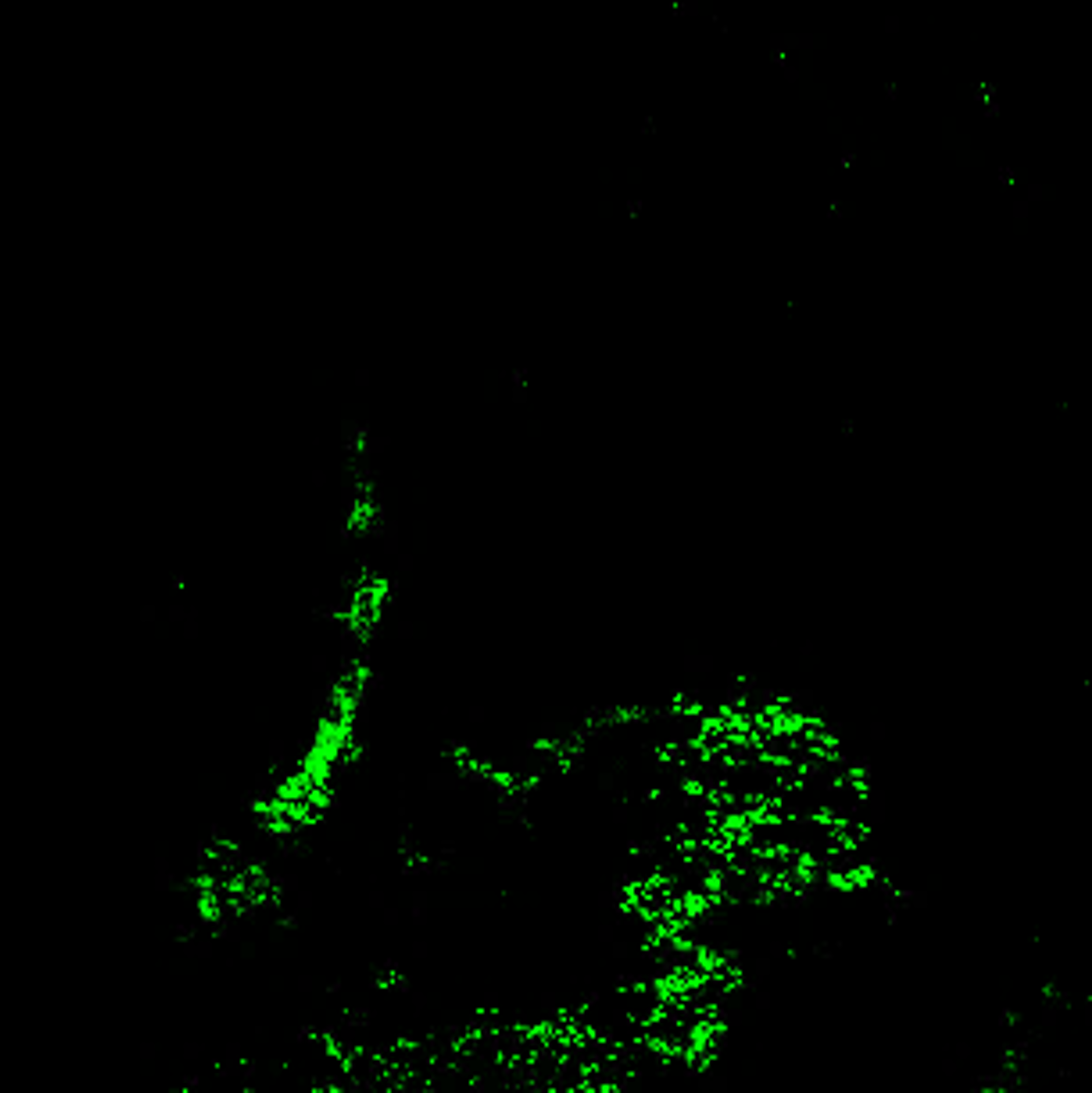

Supplement: Supplementary file 5 — Source Data for Figure 1 [file EMMM-15-e16796-s009.zip › EMMM202216796-sup-0004-SDataFig1/1A/8h/Zymosan.tif]

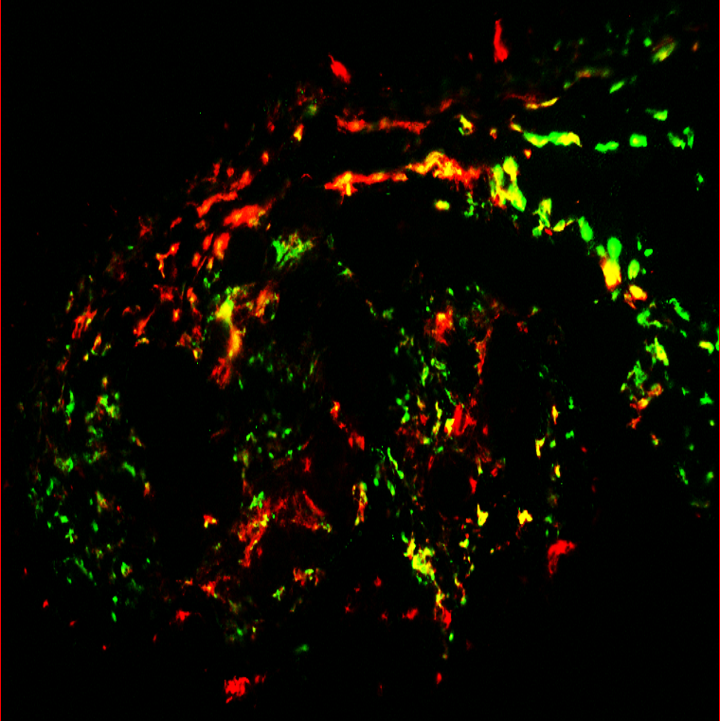

Supplement: Supplementary file 5 — Source Data for Figure 1 [file EMMM-15-e16796-s009.zip › EMMM202216796-sup-0004-SDataFig1/1A/naive/CD206_CD86.tif]

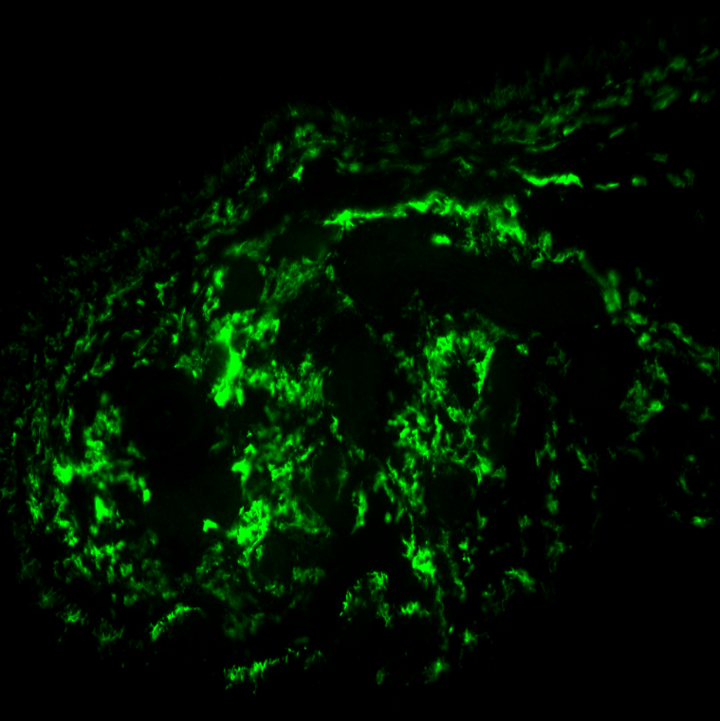

Supplement: Supplementary file 5 — Source Data for Figure 1 [file EMMM-15-e16796-s009.zip › EMMM202216796-sup-0004-SDataFig1/1A/naive/F4-80.tif]

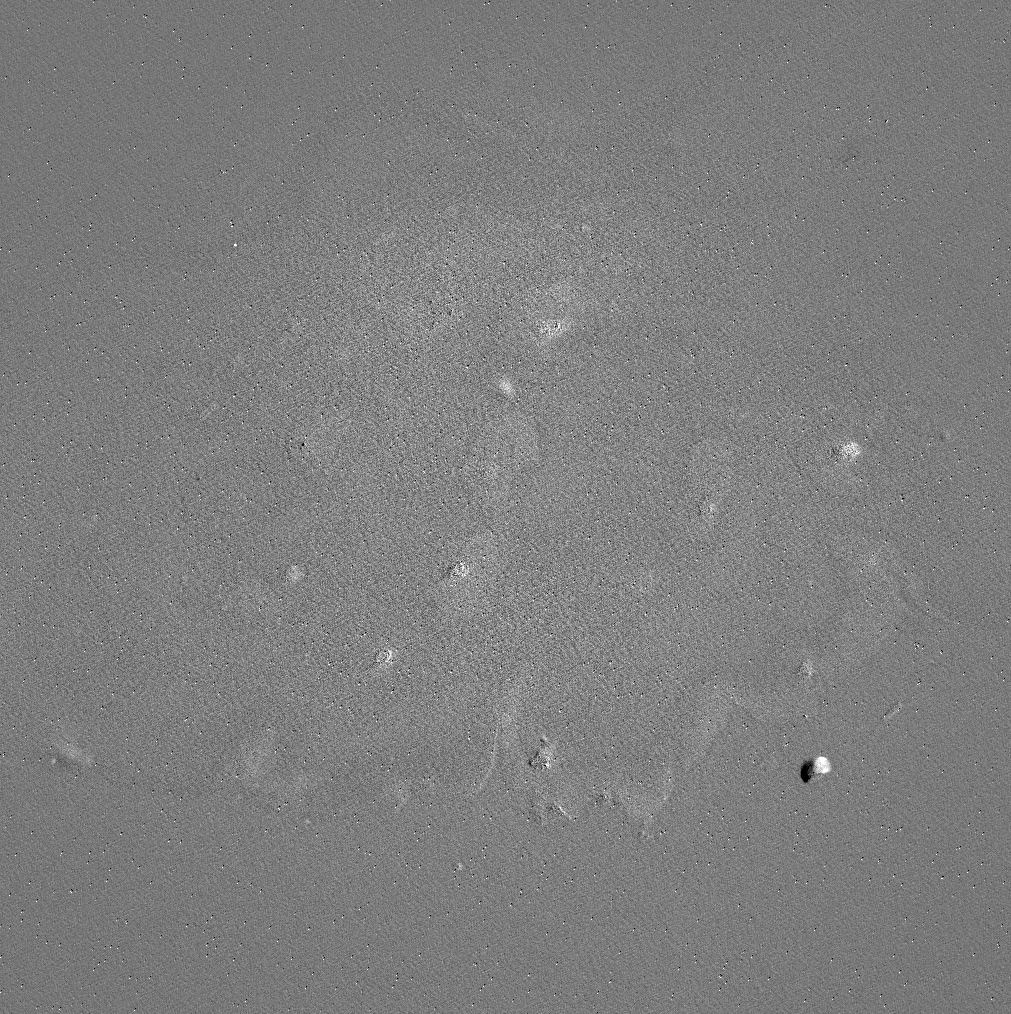

Supplement: Supplementary file 5 — Source Data for Figure 1 [file EMMM-15-e16796-s009.zip › EMMM202216796-sup-0004-SDataFig1/1A/naive/Zymosan.tif]

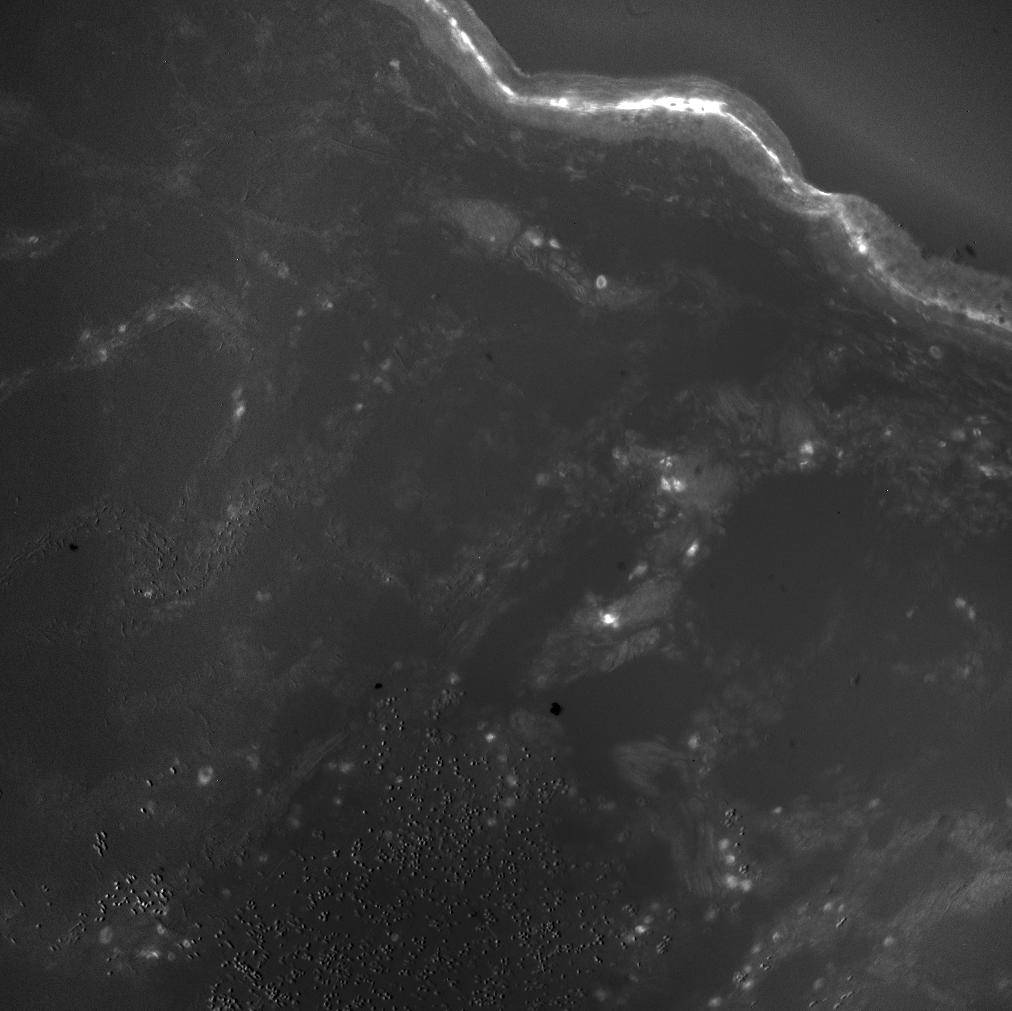

Supplement: Supplementary file 7 — Source Data for Figure 3 [file EMMM-15-e16796-s006.zip › EMMM202216796-sup-0006-SDataFig3/3C/IL4.tiff]

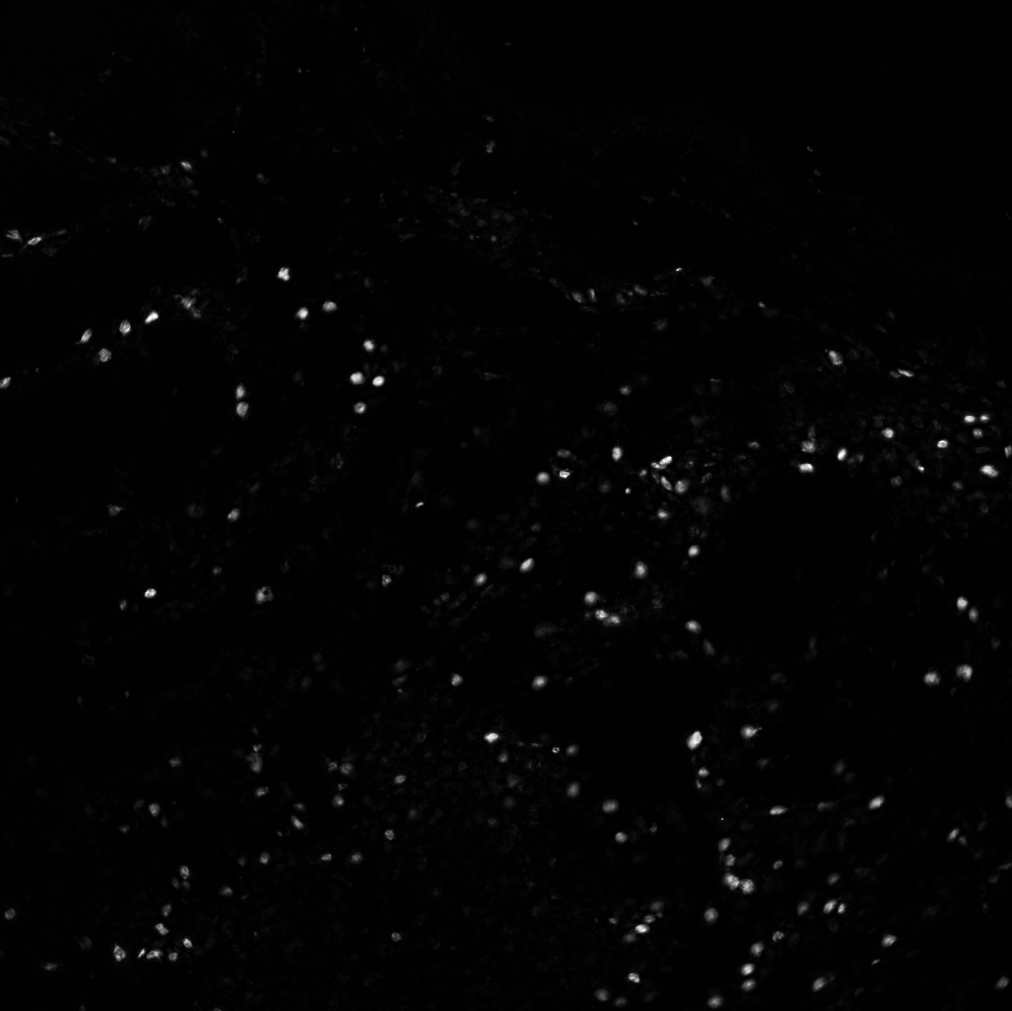

Supplement: Supplementary file 7 — Source Data for Figure 3 [file EMMM-15-e16796-s006.zip › EMMM202216796-sup-0006-SDataFig3/3C/Siglec_F.tiff]

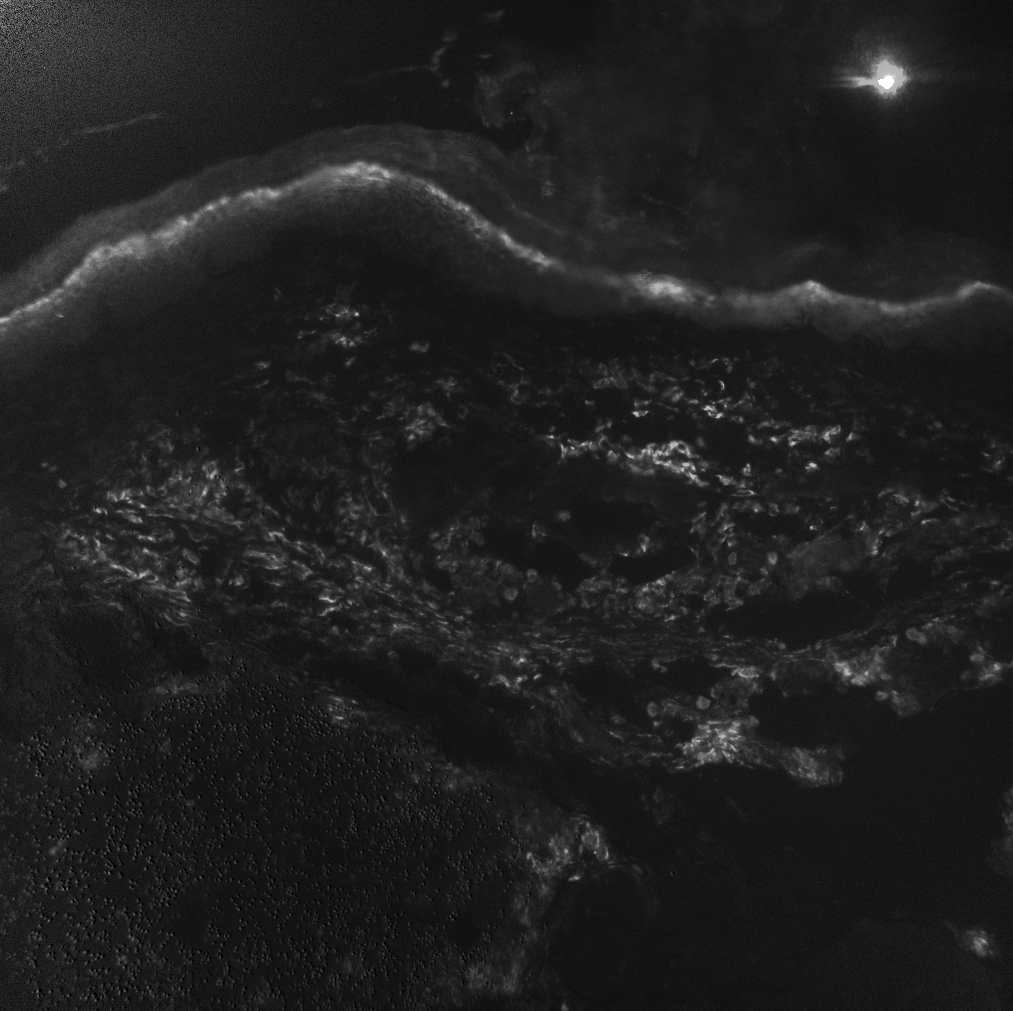

Supplement: Supplementary file 7 — Source Data for Figure 3 [file EMMM-15-e16796-s006.zip › EMMM202216796-sup-0006-SDataFig3/3D/IL4.tiff]

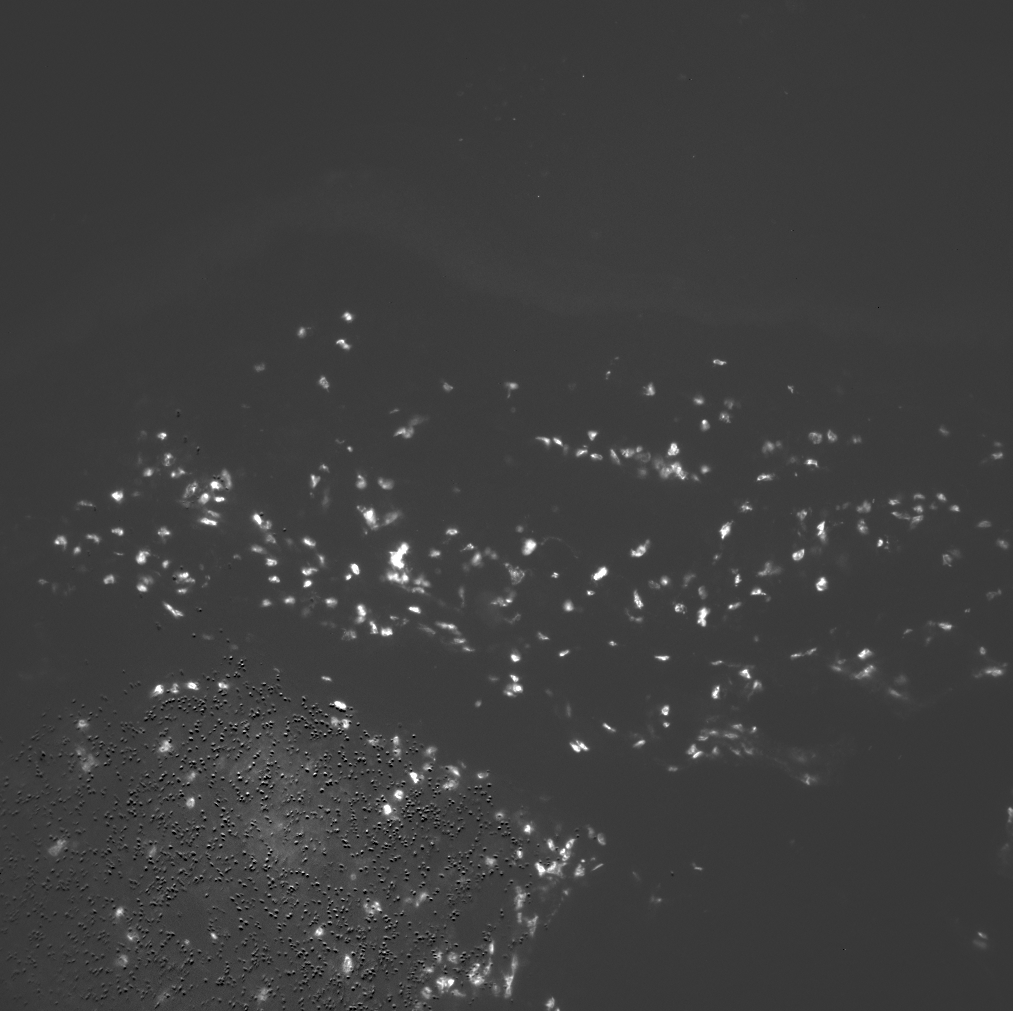

Supplement: Supplementary file 7 — Source Data for Figure 3 [file EMMM-15-e16796-s006.zip › EMMM202216796-sup-0006-SDataFig3/3D/SiglecF.tif]
