# Supplementary material for: Eosinophil‐derived IL‐4 is necessary to establish the inflammatory structure in innate inflammation
Source: EMBO Mol Med. 2022 Dec 21;15(2):e16796. doi: 10.15252/emmm.202216796 (PMC9906331; doi:10.15252/emmm.202216796)
Supplement: Supplementary file 13 [file EMMM-15-e16796-s004.pdf]

EMBO
Molecular Medicine

Eosinophil-derived IL-4 is necessary to establish the inflammatory structure in innate inflammation

Anja Kolbinger, Tim Schäufele, Hanna Steigerwald, Joschua Friedel, Sandra Pierre, Gerd Geisslinger, and Klaus Scholich
DOI: 10.15252/emmm.202216796

Corresponding author: Klaus Scholich (scholich@em.uni-frankfurt.de)

Review Timeline:

Submission Date:	25th Aug 22
Editorial Decision:	4th Oct 22
Revision Received:	21st Nov 22
Editorial Decision:	29th Nov 22
Revision Received:	6th Dec 22
Accepted:	8th Dec 22

Editor: Zeljko Durdevic

Transaction Report:

(Note: With the exception of the correction of typographical or spelling errors that could be a source of ambiguity, letters and reports are not edited. Depending on transfer agreements, referee reports obtained elsewhere may or may not be included in this compilation. Referee reports are anonymous unless the Referee chooses to sign their reports.)

4th Oct 2022

Dear Prof. Scholich,

Thank you for the submission of your manuscript to EMBO Molecular Medicine. We have now received feedback from the three reviewers who agreed to evaluate your manuscript. As you will see from the reports, while the referee #2 is overall supportive of the study, referees #1 and #3 recognize interest of the study but also raise serious concerns, particularly regarding the inappropriate controls, pathogen model and eosinophil depletion strategy.

Further consideration of a revision that addresses reviewers' concerns in full will entail a second round of review. EMBO Molecular Medicine encourages a single round of revision only and therefore, acceptance or rejection of the manuscript will depend on the completeness of your responses included in the next, final version of the manuscript. For this reason, and to save you from any frustrations in the end, I would strongly advise against returning an incomplete revision.

We would welcome the submission of a revised version within three months for further consideration. Please let us know if you require longer to complete the revision.

Please use this link to login to the manuscript system and submit your revision: https://embomolmed.msubmit.net/cgi-bin/main.plex

I look forward to receiving your revised manuscript.

Yours sincerely,

Zeljko Durdevic

Zeljko Durdevic
Editor
EMBO Molecular Medicine

***** Reviewer's comments *****

Referee #1 (Remarks for Author):

In this study the authors aimed to characterize the development of inflammatory microenvironments during a zymosan-induced paw inflammation model that mimics toll-like receptor 2-mediated inflammation. The overall aim of this study is novel especially in view of the methodology, which was used and implemented to assess the spatial and temporal appearance of cells. The main findings of the study were that:

1) Within 24 hours after zymosan injection immune cells were organized in three distinct zones comprising of a pathogen-containing "coreregion", an inflammatory-region and an external anti-inflammatory-region.
2) Polarized macrophages were distinctly located in the different regions whereas eosinophils were present in all regions.
3) Depletion of eosinophils resulted in levels of IL-4 levels, increased edema, and mechanical and thermal hypersensitivities during the resolution phase of inflammation. These parameters were restored upon exogenous delivery of IL-4
4) Eosinophils regulated the formation of the three spatially described regions
5) Eosinophils regulated neutrophil accumulation, efferocytosis and M2 macrophage polarization

These findings and conclusions are novel and substantially add to the growing understaidng regarding the heterogeneity of eosinophils and their activities in different disease contexts or in specific microenvironments.
Despite this, several issue require careful thought and re-examination since some of the conclusions are not supported by the data due to inappropriate controls.

Major comments:

1) The figures in 1D-E are confusing. It will probably be better to generate a bar graph that will display the appearance of M1 and M2 macrophages side by side (i.e., to combine Figured D and E).
2) The authors use an extremely sophisticated staining procedure of multiplex IHC. Yet, eventually they define their cellular compartments and populations using a limited set of markers such as: Ly6G, Siglec-F and F4/80. None of which is unique to one cell population. Furthermore, these markers are dynamically expressed on different myeloid cells. Thus, despite the advanced method, the conclusions are limited, and the full cellular spectrum remains unexplored.
3) How did the authors confirm eosinophil depletion? Since anti-Siglec-F used as a surface marker for the gating strategy, it is possible that anti-Sigelc F ab masked the staining by anti-Siglec-f 9 that was used for validation.
4) IN eosinophil depletion studies no control antibody was used thus, it is possible that the observed effect was independent of eosinophil activities.
5)

Referee #2 (Remarks for Author):

The authors, as well summarized in the Abstract, employ elegant MELC technology to probe the nature of cells, especially eosinophils, in the development and resolution of inflammation in a zymosan TLR elicited mouse paw model of inflammation. Eosinophils were present in the inflammatory core, a bordering proinflammatory region, and a third anti-inflammatory region. As presented, sequential experimental approaches support the conclusion that IL-4-expressing eosinophils support the resolution of inflammation.

The findings bolster increasing evidence of the multiplicity of roles for eosinophils, and their varying "phenotypes'" or "sub types." Whereas recent studies have relied on transcriptional profiling of eosinophils (a problematic issue for tissue eosinophils), notable innovative approaches here include both in situ immunostaining for cell associated cytokine proteins and assays of lesional cytokine proteins. For eosinophils that contain preformed cytokine proteins, either stored with in their granules and/or present in cytosolic transport vesicles, direct assays of preformed cytokines, including IL-4, can be more informative of more acute cellular responses than transcript analyses.

The experimental model utilizes a focal and identifiable stimulus, zymosan. As suggested by the authors similar analyses of other eosinophil associated responses (e.g., schistosome egg granulomas) will be of interest.

Referee #3 (Comments on Novelty/Model System for Author):

The pathogen models that they used is a zymosan injection model which is not the equivalent of a pathogen infection.

moreover they used Siglec F antibody for eosinophils depletion where a eosinophils deficient mice would be a better model.

Referee #3 (Remarks for Author):

The manuscript from A. Kolbinger et al. described the immunophenotopical signature of the eosinophils that are presented in the tissue in 3 regions that they defined after zymosan injection.
They would like to analyse the infection phenotype after eosinohils depletion and redefine the region in eosinophils deficient conditions. All their phenotypes observed in eosinophils deleted conditions are restore by IL4. The data and subjects are interesting, however there are several flaws in the manuscript.

1-It is quite surprising that all the phenotypes are only dependent of the production of iL4, knowing the multitudes of secretory factors specific for eosinophils, that have not been checked or addressed.
2- the pathogen models used of zymosan is not reflecting fully a pathogen infection
3- they concentrate their analyses on eosinophils, other cell type should be investigated and compared to eosinophils, notably the neutrophils.
4-they use the markers CD86 as macrophages markers to delineate the different tissue 'regions'. this marker is not specific of macrophages, CD68 should be used in this purpose.
5- the separation of the region are unclear, is there lining cells visible? the demarcations presented seems quite arbitrary draw.
6- the spatial analyses confirmed previous finding where the level of pro inflammatory go to non inflammation when the cells are at further distance of the center of 'infection'.
7-The FACS data from all paw reproduce the regions analysed, but it is difficult to understand since the cells are pooled in the paw analyses.
8- the authors show that siglec F cells are present in the 3 regions defined, but they seem absent in the first region. In the figure3D the region analysed could be part of the region 2.
9-the same is true for the IL4-siglecF co expression, their quantification should be done.
10- the deletion of 50% of eosinophils is not a deletion model, a genetic models with 100% of eosinophils deficiency should be used.
11-The results of the rescue by IL4 administration are not convincing based 1) on the macrophage markers used, 2) on the known factor as resolvins which could play an identical role.

Referee #1 (Remarks for Author):

1) The figures in 1D-E are confusing. It will probably be better to generate a bar graph that will display the appearance of M1 and M2 macrophages side by side (i.e., to combine Figured D and E).

As suggested by the reviewer we changed the figure and combined figures 1D and 1E in one Figure (Fig. 1D in the revised version).

2) The authors use an extremely sophisticated staining procedure of multiplex IHC. Yet, eventually they define their cellular compartments and populations using a limited set of markers such as: Ly6G, Siglec-F and F4/80. None of which is unique to one cell population. Furthermore, these markers are dynamically expressed on different myeloid cells. Thus, despite the advanced method, the conclusions are limited, and the full cellular spectrum remains unexplored.

We would like to clarify that cell clustering of the immune cell types was achieved based on the combinations of all markers used in the MELC analyses. For better clarity we chose to specifically name in the text only the markers, which define a certain immune cell or its subtype. In the revised manuscript we changed the text throughout the manuscript to point out that in MELC analyses always phenograph analysis-derived cell clusters were used and added a clarification at the beginning of the "Results" section (page 5).

It should be noted that the markers for B-cells, T-cells, NK cells, NK-like cells and ILCs are part of the antibody panel. However, since these cells were in the zymosan model either completely absent or appeared too rarely to fulfil the requirements for bioinformatic analysis (Fig. 2A), they do not appear in neighborhood and network analyses.

3) How did the authors confirm eosinophil depletion? Since anti-Siglec-F used as a surface marker for the gating strategy, it is possible that anti-Sigelc F ab masked the staining by anti-Siglec-F that was used for validation.

To avoid the problem of masking Siglec F by the Siglec F antibody used for depletion, we employed different anti-Siglec F antibody clones for depletion and detection. We added to the revised manuscript a FACS control with blood samples, which are either a) labelled with the detection antibody alone or b) labelled with the detection antibody in presence of equal concentrations of the depletion antibody. The data show that the presence of the depletion antibody did not interfere with its recognition by the detection antibody. The data have been added to the revised manuscript as Appendix Figure S6 and are mentioned in the "Results" section of the revised manuscript (page 8).

4) In eosinophil depletion studies no control antibody was used thus, it is possible that the observed effect was independent of eosinophil activities.

We used a rat IgG2α antibody as control for the Siglec F depletion antibody. Previously we mentioned this only in the "Material and Methods" section, but added a note on page 8 in the "Results" section of the revised manuscript to point out the usage of the control antibody.

Referee #2 (Remarks for Author):

We would like to thank Reviewer 2 for the encouraging remarks.

Referee #3 (Comments on Novelty/Model System for Author):

A) The pathogen models that they used is a zymosan injection model which is not the equivalent of a pathogen infection.

Zymosan is a ligand found in the cell wall of yeast and presents a widely used model system to study Toll-like receptor (TLR) 2-mediated innate inflammation. TLR2 is a key pattern recognition receptor that recognizes and orchestrates the immune response against gram-positive bacteria and a wide range of fungi, viruses, protozoa and helminths. We agree with the reviewer that the use of zymosan as pathogen is not directly comparable to a bacterial or fungal infection, however, we decided to use the zymosan-induced inflammation model based on (1) the importance of TLR2- mediated innate processes in a broad range of infectious diseases, (2) the specificity of zymosan to induce this immune response, and (3) the immobility of fluorophore-labelled zymosan allowing to detect its specific localization. The usage of live or dead organisms (i.e. bacteria or fungi) would have the severe disadvantage that they release (unlabeled) cell components, which are able to diffuse from the infecting organism and thereby prohibiting to localize all immunogenic pathogens. Thus, to gain robust insights in the basic structure of an inflammation in relation to the pathogen it is necessary to be able to detect all immunogenic components, which can be achieved using the zymosan model.

B) Moreover they used Siglec F antibody for eosinophils depletion where a eosinophils deficient mice would be a better model.

As the reviewer states correctly we found that injection of the anti-Siglec F antibody decreased the eosinophil number in the blood by around 50%, while eosinophils were absent at the site of inflammation in the paw. To address this discrepancy, we hypothesized that the destruction of the eosinophils leads to their phagocytosis by neutrophils and macrophages resulting in Siglec F-positive signals by these cells. Therefore, we repeated the FACS quantification of eosinophils using antibodies against all immune cells (CD45), eosinophils (Siglec F), neutrophils (Ly6G) and macrophages (CD68).

For this experiment mice received anti-Siglec F antibody 24 hours prior the zymosan injection. 24 hours after the zymosan injection FACS analyses were performed showing that, after excluding Siglec F-positive macrophages and neutrophils, in anti-Siglec F treated mice the eosinophil numbers were reduced by 90% and 92% in blood and paws, respectively (Fig. 4A-C in the revised manuscript). Most importantly, we did not detect any eosinophils in the inflamed area in MELC analyses, which demonstrates that administration of anti-Siglec F antibody completely depletes eosinophils at the site of inflammation (Fig. 5C in the revised manuscript). Also, to accommodate for the increased neutrophil number in eosinophil-depleted mice, which may lead to a disproportional decrease in the calculated eosinophil number, we changed the presentation of the MELC data from "% of CD45-positive cells" to "% of all cells" in Figure 5C-I. Notably, the change in the calculation had no effect on the significance of the results.

The data have been added to the revised manuscript as Fig. 4A-C and Figure 5C.

Referee #3 (Remarks for Author):

1-It is quite surprising that all the phenotypes are only dependent of the production of IL4, knowing the multitudes of secretory factors specific for eosinophils, that have not been checked or addressed.

We agree with the reviewer that it is unlikely that IL-4 is the only mediator of relevance released from eosinophils during TLR2-mediated inflammation. The mediators known to be released by eosinophils have mostly similar or overlapping physiological functions and work in cooperation with each other to achieve a certain effect. However, when testing level of 23 cytokines and chemokines, we found only IL-4 significantly reduced in paws of eosinophil depleted mice (Figures 6A, 7A and Appendix Figure S7). The tested antibodies included the 4 cytokines, which were seen in MELC analysis of eosinophils (IL-4, IL-6, IL-10 and IL-13) (Figures 3A and B).

Although we do not rule out the involvement of other mediators in the three observed immunological effects (neutrophil recruitment, macrophage polarization and efferocytosis), it is evident that IL-4 administration was able to rescue the eosinophil-depletion phenotype, which is in line with previous publications showing that IL-4 regulates these three processes. We conclude that eosinophil-derived IL-4 fulfils a prominent role in mediating the observed phenotypes allowing to compensate for the loss of other potentially involved eosinophil-derived mediators.

We added a clarification for this point to page 15 of the "Discussion" section of the revised manuscript.

2- the pathogen models used of zymosan is not reflecting fully a pathogen infection

Please see the answer to comment A above (Comments on Novelty/Model System for Author).

3- they concentrate their analyses on eosinophils, other cell type should be investigated and compared to eosinophils, notably the neutrophils.

We agree with the reviewer that the other cell types involved in zymosan-induced inflammation processes need to be studied for their function in developing and maintaining the inflammatory regions.

In this regard we started several projects, which are investigating the influence of different immune cell types on the regional structure and the course of an zymosan-induced inflammation. We found that deletion of the different immune cells evoked very different phenotypes in regard to the physiological read-out, their role in organizing inflammatory structures and the activation of other immune cells.

Due to the complex phenotypes these investigations cannot be integrated into one manuscript, eosinophils were chosen as example, since they were found in all three inflammatory regions and responded to the different microenvironments with distinguished cytokine expression patterns. At the same time, they turned out to be essential for the organization of the pro- and anti-inflammatory regions. Therefore, they are a very good example of how cells adapt to their microenvironment while they are at the same time forming these microenvironments.

4-they use the markers CD86 as macrophages markers to delineate the different tissue 'regions'. this marker is not specific of macrophages, CD68 should be used in this purpose.

CD68 can translocate from the cytoplasm to the cell surface of M1-like macrophages where it can be specifically detected by FFACS analyses when no permeabilization step is used. In contrast to FACS analysis, we use in the MELC analysis tissue sllices, which require a permeabilization step to allow access of the antibodies to their epitopes. This leads to the staining of intracellular and cell surface-located CD68, ruling out its use as M1-like macrophage marker. For better data comparison of the investigated macrophage population we chose to consistently use the same markers in FACS and MELC analyses and therefore chose CD86 as marker for M1-like macrophages.

We agree with the reviewer that CD86 is not macrophage-specific. For this reason, we used, as mentioned in the manuscript, for all analyses CD86 in combination with the common macrophage marker F4 80. In addition, in FACS and MELC analyses Siglec F expression was used to exclude F4 80-postive eosinophils (see gating strategy in Fig. S3). Moreover, CD86-expressing dendritic cells also expressed CD11c and MHC II, but not F4 80, and could therefore reliably distinguished from each other. These considerations were also leading to the gating strategy shown in Appendix Figure S3, which was used in all FACS analyses to determine macrophage-specific CD86 expression. For all MELC analyses only CD86-positive cell clusters attributed as macrophage-specific, if they were F4 80-positive and at the same time negative for Siglec F, CD11c and MHC II.

We added an additional clarification to the "Results" section on page 5 and 6 when the MELC analysis of macrophage clusters and the FACS analysis of macrophage subtypes are mentioned for the first time.

5- the separation of the region are unclear, is there lining cells visible? the demarcations presented seems quite arbitrary draw.

The overall regional structure concept was based on the distribution of the major immune cell populations derived by the statistical neighborhood analyses based on the probability of the occurrence of e.g. a M1-like macrophage neighboring a neutrophil. In this example the statistical description of the neighborhood of M1-like macrophages does not imply that all M1-like macrophages are necessarily neighbors of neutrophils. Therefore, a certain diffuse distribution and exceptional localization of some cells is allowed and expected. Therefore, a clear lining is not expected and would not represent the flexible nature of an inflammation, which is based on highly mobile immune cells. Instead of a clear lining a transition of one region to the next is seen, where for example M1- and M2-like occur together.

The lines shown in the figures are used to illustrate the area where one region transitions to the next region and are based on the following criteria: The core region is defined by the presence of zymosan. Transition of the PI-region to the AI-region occurs where M2-like macrophage outnumber M1-like macrophages. The outer limit of the AI region ends with the disappearance of M2-like macrophages.

We added to the figure legends the following statement: "The dotted lines depict the area where the transition between the neighboring regions occurs.".

6- the spatial analyses confirmed previous finding where the level of pro inflammatory go to non inflammation when the cells are at further distance of the center of 'infection'.

We agree with the reviewer that current notion is a gradual transition from pro-inflammatory sites to non-inflammation. We show in our manuscript that this view needs refinement, since 1) different pro-inflammatory regions can be distinguished depending on their immune cell content and, 2) more importantly, that a specialized anti-inflammatory region separates the non-inflammatory regions from the pro-inflammatory regions. This is a new concept in the sense that pro- and anti-inflammatory regions coexist throughout an ongoing inflammation. Therefore, our data refines the idea of a temporal transition of pro-inflammation to anti-inflammation and resolution by showing a spatial separation of coexisting opposing inflammatory processes.

7-The FACS data from all paw reproduce the regions analysed, but it is difficult to understand since the cells are pooled in the paw analyses.

The finding that the results in the MELC analysis are repeated in the FACS analysis of the total paw tissue, supports and strengthen the point that the majority of M1-like and M2-like macrophages are indeed confounded to the specific inflammatory regions described in the manuscript. It should be noted that the zymosan-containing region is only partly covered by the MELC images but that these images are representative for the regional structure outside the chosen field of visions.
We added a clarification of this point to the "Results" and "Discussion" sections (page 6 and 13) of the revised manuscript.

8- the authors show that siglec F cells are present in the 3 regions defined, but they seem absent in the first region. In the figure3D the region analysed could be part of the region 2.
As suggested by the reviewer we changed the magnified site in Figure 3D to a region deeper in the core region, which shows the presence of eosinophils in this region.

9-the same is true for the IL4-siglecF co expression, their quantification should be done.
As suggested by the reviewer we changed the magnified site in Figure 3D to a region deeper in the core region. We also quantified the number of IL-4 expressing cells in the three regions and confirmed that eosinophils in the core region do nor express IL-4.
The data have been added to the revised manuscript as Figures 3E and F.

10- the deletion of 50% of eosinophils is not a deletion model, a genetic models with 100% of eosinophils deficiency should be used.
Please see the answer to comment B above (Comments on Novelty/Model System for Author).

11-The results of the rescue by IL4 administration are not convincing based
1) on the macrophage markers used,
Please see the response to comment 4 for the explanations of the FACS gating strategy for macrophage populations and the marker use for identifying cell clusters representing macrophages in MELC analyses.

2) on the known factor as resolvins which could play an identical role.
We investigated a possible role of special proresolving mediators (SPMs) such as resolvins (Rv) and lipoxins (LX) in the resolution of inflammation of our model but were unable to detect any SPMs in the paw tissue. The SPMs were determined by LC-MS/MS using established methods (Mainka et al., 2022; Toewe et al., 2018) and included Maresin 1, RvD1/AT-RvD1, RvD2, LXA4/15-epi-LXA4, 6-epi-LXA4, LXA5, PD1 and PDx. Since detection levels were as low as 8 pg/ml, a major role of SPMs in the zymosan-induced inflammation model under the described experimental parameters seems unlikely, but may play a significant role in other inflammation models.

Reference

Mainka M, George S, Angioni C, Ebert R, Goebel T, Kampschulte N, Krommes A, Weigert A, Thomas D, Schebb NH, Steinhilber D, Kahnt AS. On the biosynthesis of specialized pro-resolving mediators in human neutrophils and the influence of cell integrity. Biochim Biophys Acta Mol Cell Biol Lipids. 2022 Mar;1867(3):159093. doi: 10.1016/j.bbalip.2021.159093

Toewe A, Balas L, Durand T, Geisslinger G, Ferreirós N. Anal Chim Acta. Simultaneous determination of PUFA-derived pro-resolving metabolites and pathway markers using chiral chromatography and tandem mass spectrometry. 2018 Nov 15;1031:185-194. doi: 10.1016/j.aca.2018.05.020.

29th Nov 2022

Dear Prof. Scholich,

Thank you for the submission of your revised manuscript to EMBO Molecular Medicine. I am pleased to inform you that we will be able to accept your manuscript pending the following final amendments:

1) Authors: E-mail correspondence to Joschua Friedel could not be delivered. Please update his e-mail address and make sure to enter correct e-mail addresses for all authors in our submission system.
2) In the main manuscript file, please do the following:
- Correct/answer the track changes suggested by our data editors by working from the attached document.
- Remove text highlight color.
- Remove the list of suppl. material.
- Correct callouts for "Suppl. Table" to "Appendix Table S1".
- In M&M, a statistical paragraph should reflect all information that you have filled in the Authors Checklist, especially regarding randomization, blinding, replication.
- Please rename "Competing Interest" to "Disclosure Statement & Competing Interests" and move it after the "Acknowledgements". We updated our journal's competing interests policy in January 2022 and request authors to consider both actual and perceived competing interests. Please review the policy https://www.embopress.org/competing-interests and update your competing interests if necessary.
- Author contributions: Please remove it from the manuscript and specify author contributions in our submission system. CRediT has replaced the traditional author contributions section because it offers a systematic machine-readable author contributions format that allows for more effective research assessment. You are encouraged to use the free text boxes beneath each contributing author's name to add specific details on the author's contribution. More information is available in our guide to authors:
https://www.embopress.org/page/journal/17574684/authorguide#authorshipguidelines
- In data availability statement, if no data are deposited in public repositories, please add the sentence: "This study includes no data deposited in external repositories". Please place the statement after M&M section.
3) Appendix: Please renumber all the figures to Appendix Figure S1-7.
4) The Paper Explained: Please move it to the main manuscript text.
5) Synopsis:
- Synopsis image: Please resize the image to 550 px-wide x (250-400)-px high and submit it as a high-resolution jpeg file.
- Synopsis text: Please submit it as a separate .doc file.
- Please check your synopsis text and image before submission with your revised manuscript. Please be aware that in the proof stage minor corrections only are allowed (e.g., typos).
6) For more information: This space should be used to list relevant web links for further consultation by our readers. Could you identify some relevant ones and provide such information as well? Some examples are patient associations, relevant databases, OMIM/proteins/genes links, author's websites, etc...
7) As part of the EMBO Publications transparent editorial process initiative (see our Editorial at http://embomolmed.embopress.org/content/2/9/329), EMBO Molecular Medicine will publish online a Review Process File (RPF) to accompany accepted manuscripts. This file will be published in conjunction with your paper and will include the anonymous referee reports, your point-by-point response and all pertinent correspondence relating to the manuscript. Let us know whether you agree with the publication of the RPF and as here, if you want to remove or not any figures from it prior to publication. Please note that the Authors checklist will be published at the end of the RPF.
8) Please provide a point-by-point letter INCLUDING my comments as well as the reviewer's reports and your detailed responses (as Word file).

I look forward to reading a new revised version of your manuscript as soon as possible.

Yours sincerely,

Zeljko Durdevic

Zeljko Durdevic
Editor
EMBO Molecular Medicine

***** Reviewer's comments *****

Referee #1 (Comments on Novelty/Model System for Author):

n this study the authors aimed to characterize the development of inflammatory microenvironments during a zymosan-induced paw inflammation model that mimics toll-like receptor 2-mediated inflammation. The overall aim of this study is novel especially in view of the methodology, which was used and implemented to assess the spatial and temporal appearance of cells. The methodology and analyses, which were used are novel and the findings are interesting

Referee #1 (Remarks for Author):

No comments.
The authors addressed all of the queries

Referee #3 (Comments on Novelty/Model System for Author):

The authors have explained clearer the use of this specific model in the revised version

Referee #3 (Remarks for Author):

The authors have substantially added explanations and new experiments to support their findings. I have no further comments

The authors performed the requested editorial changes.

We are pleased to inform you that your manuscript is accepted for publication and is now being sent to our publisher to be included in the next available issue of EMBO Molecular Medicine.

EMBO Press Author Checklist

Corresponding Author Name: Klaus Scholich
EMBO Mol Med
Manuscript Number: EMM-2022-16796-V2

USEFUL LINKS FOR COMPLETING THIS FORM
The EMBO Journal - Author Guidelines
EMBO Reports - Author Guidelines
Molecular Systems Biology - Author Guidelines
EMBO Molecular Medicine - Author Guidelines

Reporting Checklist for Life Science Articles (updated January 2022)
This checklist is adapted from Materials Design Analysis Reporting (MDAR) Checklist for Authors. MDAR establishes a minimum set of requirements in transparent reporting in the life sciences (see Statement of Task: 10.31222/osf.io/9sm4x). Please follow the journal's guidelines in preparing your manuscript.
Please note that a copy of this checklist will be published alongside your article.

Abridged guidelines for figures
1. Data
The data shown in figures should satisfy the following conditions:
- the data were obtained and processed according to the field's best practice and are presented to reflect the results of the experiments in an accurate and unbiased manner.
- ideally, figure panels should include only measurements that are directly comparable to each other and obtained with the same assay.
- plots include clearly labeled error bars for independent experiments and sample sizes. Unless justified, error bars should not be shown for technical replicates.
- if n<5, the individual data points from each experiment should be plotted. Any statistical test employed should be justified.
- Source Data should be included to report the data underlying figures according to the guidelines set out in the authorship guidelines on Data Presentation.

2. Captions
Each figure caption should contain the following information, for each panel where they are relevant:
- a specification of the experimental system investigated (eg cell line, species name).
- the assay(s) and method(s) used to carry out the reported observations and measurements.
- an explicit mention of the biological and chemical entity(ies) that are being measured.
- an explicit mention of the biological and chemical entity(ies) that are altered/varied/perturbed in a controlled manner.
- the exact sample size (n) for each experimental group/condition, given as a number, not a range;
- a description of the sample collection allowing the reader to understand whether the samples represent technical or biological replicates (including how many animals, litters, cultures, etc.).
- a statement of how many times the experiment shown was independently replicated in the laboratory.
- definitions of statistical methods and measures:
  - common tests, such as t-test (please specify whether paired vs. unpaired), simple χ2 tests, Wilcoxon and Mann-Whitney tests, can be unambiguously identified by name only, but more complex techniques should be described in the methods section;
  - are tests one-sided or two-sided?
  - are there adjustments for multiple comparisons?
  - exact statistical test results, e.g., P values = x but not P values < x;
  - definition of 'center values' as median or average;
  - definition of error bars as s.d. or s.e.m.

Please complete ALL of the questions below.
Select "Not Applicable" only when the requested information is not relevant for your study.

Materials

Newly Created Materials	Information included in the manuscript?	In which section is the information available? (Reagents and Tools Table, Materials and Methods, Figures, Data Availability Section)
New materials and reagents need to be available; do any restrictions apply?	Yes	Material and Methods

Antibodies	Information included in the manuscript?	In which section is the information available? (Reagents and Tools Table, Materials and Methods, Figures, Data Availability Section)
For **antibodies** provide the following information:<br>- Commercial antibodies: RRID (if possible) or supplier name, catalogue number and or/clone number<br>- Non-commercial: RRID or citation	Yes	Appendix

DNA and RNA sequences	Information included in the manuscript?	In which section is the information available? (Reagents and Tools Table, Materials and Methods, Figures, Data Availability Section)
**Short novel DNA or RNA including primers, probes**: provide the sequences.	Not Applicable	

Cell materials	Information included in the manuscript?	In which section is the information available? (Reagents and Tools Table, Materials and Methods, Figures, Data Availability Section)
**Cell lines:** Provide species information, strain. Provide accession number in repository **OR** supplier name, catalog number, clone number, and/**OR** RRID.	Not Applicable	
**Primary cultures:** Provide species, strain, sex of origin, genetic modification status.	Not Applicable	
Report if the cell lines were recently **authenticated** (e.g., by STR profiling) and tested for mycoplasma contamination.	Not Applicable	

Experimental animals	Information included in the manuscript?	In which section is the information available? (Reagents and Tools Table, Materials and Methods, Figures, Data Availability Section)
**Laboratory animals or Model organisms:** Provide species, strain, sex, age, genetic modification status. Provide accession number in repository **OR** supplier name, catalog number, clone number, **OR** RRID.	Yes	Material and Methods
**Animal observed in or captured from the field:** Provide species, sex, and age where possible.	Not Applicable	
Please detail **housing and husbandry conditions**.	Yes	Material and Methods

Plants and microbes	Information included in the manuscript?	In which section is the information available? (Reagents and Tools Table, Materials and Methods, Figures, Data Availability Section)
**Plants:** provide species and strain, ecotype and cultivar where relevant, unique accession number if available, and source (including location for collected wild specimens).	Not Applicable	
**Microbes:** provide species and strain, unique accession number if available, and source.	Not Applicable	

Human research participants	Information included in the manuscript?	In which section is the information available? (Reagents and Tools Table, Materials and Methods, Figures, Data Availability Section)
If collected and within the bounds of privacy constraints report on age, sex and gender or ethnicity for all study participants.	Not Applicable	

Core facilities	Information included in the manuscript?	In which section is the information available? (Reagents and Tools Table, Materials and Methods, Figures, Data Availability Section)
If your work benefited from core facilities, was their service mentioned in the acknowledgments section?	Not Applicable	

Design

**Study protocol**	**Information included in the manuscript?**	**In which section is the information available?** (Reagents and Tools Table, Materials and Methods, Figures, Data Availability Section)
If study protocol has been **pre-registered, provide DOI in the manuscript**. For clinical trials, provide the trial registration number **OR** cite DOI.	Not Applicable	
Report the **clinical trial registration number** (at ClinicalTrials.gov or equivalent), where applicable.	Not Applicable	

**Laboratory protocol**	**Information included in the manuscript?**	**In which section is the information available?** (Reagents and Tools Table, Materials and Methods, Figures, Data Availability Section)
Provide DOI OR other citation details if **external detailed step-by-step protocols** are available.	Not Applicable	

**Experimental study design and statistics**	**Information included in the manuscript?**	**In which section is the information available?** (Reagents and Tools Table, Materials and Methods, Figures, Data Availability Section)
Include a statement about **sample size** estimate even if no statistical methods were used.	Not Applicable	
Were any steps taken to minimize the effects of subjective bias when allocating animals/samples to treatment (e.g. **randomization procedure**)? If yes, have they been described?	Yes	The mice were same age and same sex. They were provided by one commercial source and were seperated at the arrival. The mice were randomized for the experiment
Include a statement about **blinding** even if no blinding was done.	Yes	The researcher was blinded when performing behavioral tests or edema measurements.
Describe **inclusion/exclusion criteria** if samples or animals were excluded from the analysis. Were the criteria pre-established?<br><br>If sample or data points were omitted from analysis, report if this was due to attrition or intentional exclusion and provide justification.	Not Applicable	
For every figure, are **statistical tests** justified as appropriate? Do the data meet the assumptions of the tests (e.g., normal distribution)? Describe any methods used to assess it. Is there an estimate of variation within each group of data? Is the variance similar between the groups that are being statistically compared?	Yes	Material and Methods, Figure legends

**Sample definition and in-laboratory replication**	**Information included in the manuscript?**	**In which section is the information available?** (Reagents and Tools Table, Materials and Methods, Figures, Data Availability Section)
In the figure legends: state number of times the experiment was **replicated** in laboratory.	Yes	Figure legends
In the figure legends: define whether data describe **technical or biological replicates**.	Yes	n-numbers describe bilogical replicates. Figure legends, Material and Methods

Ethics

**Ethics**	**Information included in the manuscript?**	**In which section is the information available?** (Reagents and Tools Table, Materials and Methods, Figures, Data Availability Section)
Studies involving **human participants**: State details of **authority granting ethics approval** (IRB or equivalent committee(s), provide reference number for approval.	Not Applicable	
Studies involving **human participants**: Include a statement confirming that **informed consent** was obtained from all subjects and that the experiments conformed to the principles set out in the WMA Declaration of Helsinki and the Department of Health and Human Services Belmont Report.	Not Applicable	
Studies involving **human participants:** For publication of **patient photos**, include a statement confirming that consent to publish was obtained.	Not Applicable	
Studies involving experimental **animals**: State details of **authority granting ethics approval** (IRB or equivalent committee(s), provide reference number for approval. Include a statement of compliance with ethical regulations.	Yes	Material and Methods
Studies involving **specimen and field samples:** State if relevant **permits** obtained, provide details of authority approving study; if none were required, explain why.	Not Applicable	

**Dual Use Research of Concern (DURC)**	**Information included in the manuscript?**	**In which section is the information available?** (Reagents and Tools Table, Materials and Methods, Figures, Data Availability Section)
Could your study fall under dual use research restrictions? Please check biosecurity documents and list of **select agents and toxins** (CDC): https://www.selectagents.gov/sat/list.htm	Not Applicable	
If you used a select agent, is the security level of the lab appropriate and reported in the manuscript?	Not Applicable	
If a study is subject to dual use research of concern regulations, is the name of the **authority granting approval and reference number** for the regulatory approval provided in the manuscript?	Not Applicable	

Reporting

The MDAR framework recommends adoption of discipline-specific guidelines, established and endorsed through community initiatives. Journals have their own policy about requiring specific guidelines and recommendations to complement MDAR.

**Adherence to community standards**	**Information included in the manuscript?**	**In which section is the information available?** (Reagents and Tools Table, Materials and Methods, Figures, Data Availability Section)
State if relevant guidelines or checklists (e.g., **ICMJE, MIBBI, ARRIVE, PRISMA**) have been followed or provided.	Not Applicable	
For **tumor marker prognostic studies,** we recommend that you follow the **REMARK** reporting guidelines (see link list at top right). See author guidelines, under 'Reporting Guidelines'. Please confirm you have followed these guidelines.	Not Applicable	
For **phase II and III randomized controlled trials**, please refer to the **CONSORT** flow diagram (see link list at top right) and submit the CONSORT checklist (see link list at top right) with your submission. See author guidelines, under 'Reporting Guidelines'. Please confirm you have submitted this list.	Not Applicable	

Data Availability

**Data availability**	**Information included in the manuscript?**	**In which section is the information available?** (Reagents and Tools Table, Materials and Methods, Figures, Data Availability Section)
Have **primary datasets** been deposited according to the journal's guidelines (see 'Data Deposition' section) and the respective accession numbers provided in the Data Availability Section?	Not Applicable	
Were **human clinical and genomic datasets** deposited in a public access-controlled repository in accordance to ethical obligations to the patients and to the applicable consent agreement?	Not Applicable	
Are **computational models** that are central and integral to a study available without restrictions in a machine-readable form? Were the relevant accession numbers or links provided?	Yes	Material and Methods
If publicly available data were reused, provide the respective **data citations in the reference list**.	Not Applicable	
